# Controlled Lattice Distortion and Lattice‐Strain‐Engineered CoCuIr Alloy toward Preferential Chlorine Tolerant Direct Seawater Electrolysis

**DOI:** 10.1002/advs.77531

**Published:** 2026-08-31

**Authors:** Ankur Kumar, Abhinav Yadav, Saloni Latiyan, Palash J. Thakuria, Ankur K. Guha, Sasanka Deka

**Affiliations:** ^1^ Department of Chemistry North Campus University of Delhi Delhi India; ^2^ Advanced Computational Chemistry Centre Cotton University Guwahati India

**Keywords:** direct seawater electrolysis, H_2_ evolution, induced strain, lattice distortion, lattice‐strain‐engineering, O_2_ evolution, trimetallic alloy

## Abstract

Direct seawater electrolysis (DSE) is a highly promising process for hydrogen production; however, the competing chlorine chemistry in seawater hinders the O_2_ evolution reaction, and the corrosive environment poses major catalytic challenges. Herein, we meticulously design a lattice‐strain‐engineered Co–Cu–Ir trimetallic alloy nanoparticle (NP) electrocatalyst for efficient seawater electrolysis by carefully selecting and tuning the concentration of larger Ir into the smaller CoCu lattice. This insertion induces pronounced lattice distortion and compressive strain by shortening Cu─Co bond distances, generating rich active sites. Optimized Co_0.28_Cu_0.67_Ir_0.05_ exhibits best performance, enabling DSE in untreated alkaline seawater with low cell voltage (<1.5 V at 10 mA cm^‒2^), sustained ampere‐level current densities (>2.0 A cm^‒2^, 1.85 V), and long‐term stability at 500 mA cm^‒2^. Strain mapping suggests the coexistence of localized compressive and tensile strain. H_2_ evolution occurs at the strain‐induced active sites (Ir/Cu), while O_2_ evolution occurs at the Ir‐centered Ir/Co sites. Computational studies reveal Ir‐induced d‐band modulation that optimizes hydrogen adsorption and enhances both hydrogen and oxygen evolution. This work demonstrates that lattice‐strain engineering, coupled with particle‐size reduction, increased surface area, abundant active sites, and electronic modulation in trimetallic alloys, enables preferential chlorine‐tolerant seawater electrolysis.

## Graphical abstract

A lattice‐strain‐engineered Co_0.28_Cu_0.67_Ir_0.05_ trimetallic alloy nanoparticle electrocatalyst is rationally designed for efficient alkaline seawater electrolysis. Larger Ir atoms in the CoCu lattice modulate the local coordination environment, generating compressive lattice strain and highly active catalytic sites. It exhibited superior activity with less chlorine affinity, delivering overall water splitting at low cell voltages (<1.5 V), ampere‐level current densities (>2.0 A cm^−^^2^), and long‐term stability.

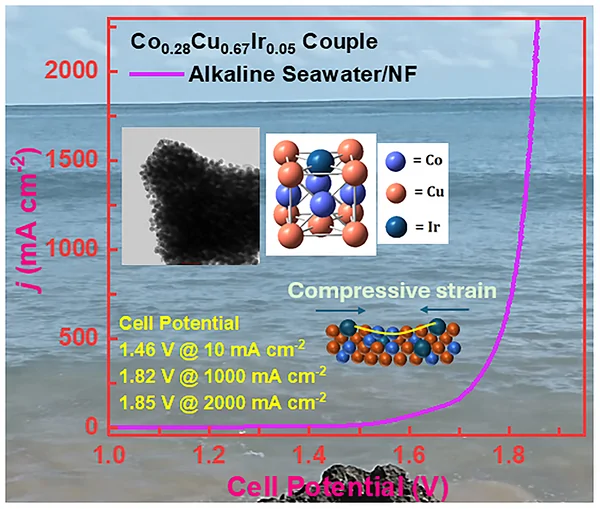

## 1 Introduction

Hydrogen, with its high energy density and eco‐friendly nature, is a strong candidate for green energy and a sustainable alternative to fossil fuels [1, 2, 3, 4]. Electrocatalytic water splitting, a cutting‐edge technology, offers a promising route for hydrogen production in acidic or alkaline media [5, 6, 7]. However, the large‐scale deployment of water electrolysis is challenged by increasing freshwater demand. Direct seawater electrolysis (DSE) offers a viable solution by utilizing abundant seawater resources, but its high chloride concentration leads to competing chlorine evolution reactions (ClER) at the anode rather than the oxygen evolution reaction (OER) [8, 9, 10]. The 2e^−^ Cl^−^ oxidation reaction (ClOR) competes with the 4e^−^ OER, leading to the formation of either Cl_2_ in acidic media or hypochlorite (ClO^−^) in alkaline media (pH>7.5), instead of O_2_. This leads to significant electrode corrosion, reducing cell efficiency [11, 12]. At high pH, the ClER has a higher thermodynamic potential than the OER, with a maximum fixed gap of ∼480–540 mV (Cl^−^ + 2OH^−^ → ClO^−^ + H_2_O + 2e^−^, E^0^ = 1.72 V vs RHE). This difference creates a “safe zone” where OER can proceed while the potential remains below that required for ClO^−^ formation, thereby thermodynamically suppressing ClER [13, 14, 15, 16, 17, 18]. However, under alkaline conditions, Mg^2+^ and Ca^2+^ can form metal hydroxide precipitates that block catalyst active sites, membrane pores, and key electrolyzer components. Although indirect seawater electrolysis (ISE) employs pretreatment to remove dissolved salts, organics, microplastics, etc., it is more expensive than DSE, despite the latter's greater technical challenges.

Over the past decade, extensive efforts in catalyst design, driven by deeper mechanistic insights, have guided interface engineering, doping, surface reconstruction, single‐atom sites, chloride‐repelling secondary layers, selective adsorption strategies, etc., thereby accelerating progress in DSE by suppressing ClER [16, 19, 20, 21, 22, 23, 24]. However, most studies suffer from intrinsic OER selectivity over ClER and lower OER Faradaic efficiency, long‐term stability at industrial current density (∼500–1000 mA cm^−^^2^), mechanistic understanding in real seawater, and electrode‐electrolyte interface control [11, 25]. These critical problems highlight the need to better understand the critical role of a new efficient electrocatalyst's surface in this harsh environment. Another Cl‐related major issue is that Cl^−^–OH^−^ formation progressively transforms the surface metal into metal hydroxides, leading to active‐site deactivation under oxidative potentials. All these challenges and gaps raise a fundamental question: can a new electrocatalyst be designed to selectively favor OER over ClER without requiring a secondary Cl^−^ tolerance process and to be equally useful for the hydrogen evolution reaction (HER) at the cathode?

This work aims to develop a lattice‐strain‐engineered CoCuIr alloy electrocatalyst that selectively drives both OER and HER in alkaline seawater electrolysis. By leveraging controlled lattice distortion to enable self‐selective OH^−^ adsorption over Cl^−^, the catalyst is designed to deliver high current densities of 2.0 A cm^−^^2^ at a low cell voltage. We first synthesized bimetallic CoCu alloy NPs, where both Co and Cu have an *fcc* structure with similar atomic radii and lattice parameters, resulting in minimal lattice mismatch, stress/strain, and limiting active site formation. Controlled lattice‐strain engineering induces defects that enhance electrolyte adsorption, tune the electronic structure, and optimize adsorption energies, thereby improving reaction kinetics [26, 27, 28]. We developed a strategy to engineer lattice mismatch in the CoCu alloy by reducing Co─Cu bond distances and NP size. We chose heavy Ir because its OER activity trend is Au ≤ Pt < Ir < Ru ≤ Os and stability trend is Au ≥ Pt > Ir > Ru ≥ Os to enhance activity, as well as it reduces the binding energy difference of the ^*^O and ^*^OOH intermediates during OER, thus a CoCuIr is expected to work well in a seawater environment [29, 30, 31]. We designed optimized Co_0.28_Cu_0.67_Ir_0.05_ alloy NPs by controlling the metal ratios, thereby optimizing lattice mismatch for optimal output. It delivers superior overall water splitting (OWS) in both freshwater and seawater, achieving <1.5 V at 2.0 A cm^−^^2^ with excellent long‐term stability. Bifunctional activity is minimal without Ir (no strain) or with excess Ir (excessive strain). During electrolysis, pre‐existing Co^2+^/Co^3+^ and Cu^2+^ hard Lewis‐acidic sites at the surface exhibit a significantly higher affinity for OH^−^ (a hard base) compared to Cl^−^ (a softer base). Density functional theory (DFT) calculations reveal that Ir, in synergy with Cu, lowers the HER adsorption free energy and modulates the d‐band near the Fermi level, while its interaction with Co optimizes OER activity.

## 2 Result and Discussion

### 2.1 Evolution of Lattice‐Strain‐Engineered CoCuIr Trimetallic Alloy NPs

**FIGURE 1:**
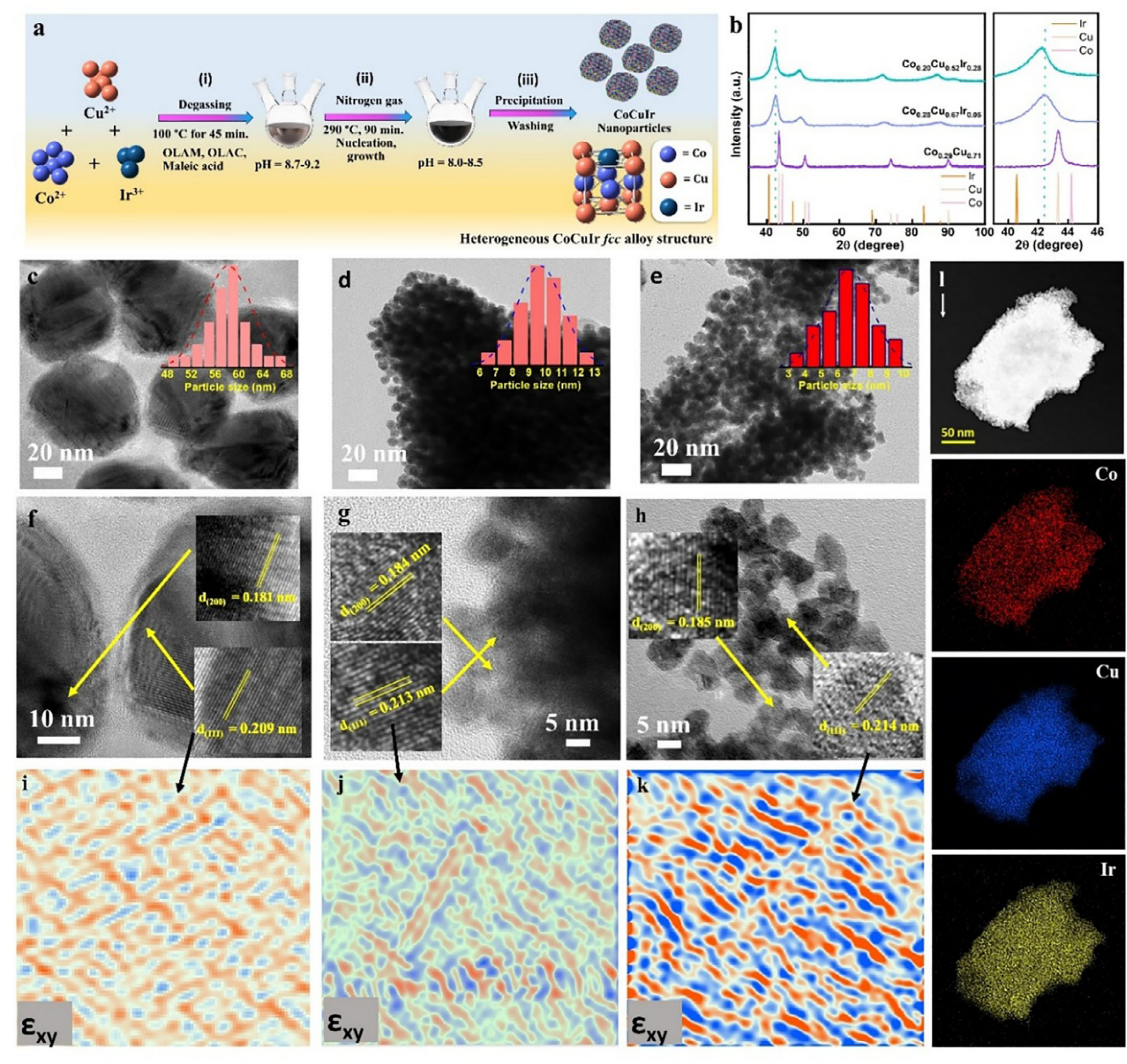
(a) Schematic diagram showing the formation of *fcc*‐CoCuIr heterogeneous alloy NPs via colloidal approach. (b) Powder XRD patterns of all the samples and of the enlarged region. Low‐magnification TEM images (c–e), HRTEM images (f–h), and strain mapping along ε_xy_ direction evaluated using *d*‐spacing HRTEM images (i–k) of Co_0.29_Cu_0.71_, Co_0.28_Cu_0.67_Ir_0.05_, and Co_0.20_Cu_0.52_Ir_0.28_, respectively. (l) STEM‐HAADF image of Co_0.28_Cu_0.67_Ir_0.05_ NPs and HAADF‐STEM elemental mapping of Co, Cu, and Ir.

Trimetallic CoCuIr NPs were meticulously designed and synthesized by a high‐temperature colloidal method (Figure 1a) using OLAM, OLAC, and maleic acid as reducing agents, solvents, and surfactants, respectively, which also act as capping agents. After degassing (i), the mixture was heated under N_2_ at an optimized 290°C for 90 min to mix alloying elements properly, allow nucleation and crystal growth (ii), and then cooled to solidify the homogeneous metallic solution (iii). The rationale for developing trimetallic CoCuIr NPs, including the selection of Ir as the third metal, surfactants, reaction time, and temperature, was derived from preliminary controlled studies. The electrochemical performance of bimetallic Co_0.29_Cu_0.71_ alloy NPs (discussed in a later section) suggests the need for a more active and stable component to enhance performance. Thus, we introduced Ir into the CoCu alloy in low content (Co_0.28_Cu_0.67_Ir_0.05_) and high content (Co_0.20_Cu_0.52_Ir_0.28_). The stoichiometries were confirmed by the inductively coupled plasma mass spectrometry (ICP‐MS) analysis (Table S1). The controlled studies on the Co_0.28_Cu_0.67_Ir_0.05_ sample are described in the supporting file. In brief, synthesis at low temperature (Co_0.28_Cu_0.67_Ir_0.05_‐200°C), without maleic acid (Co_0.28_Cu_0.67_Ir_0.05_‐No MA) and without OLAM (Co_0.28_Cu_0.67_Ir_0.05_‐No OA) does not give either monodispersed smaller alloy NPs or pure phase (Figures S1 and S2).

The powder X‐ray diffraction (XRD) patterns of Co_0.28_Cu_0.67_Ir_0.05_ and Co_0.20_Cu_0.52_Ir_0.28_ show four peaks between those of *fcc*‐Ir and *fcc*‐Cu, suggesting solid‐solution formation (Figure 1b). They exhibit peaks corresponding to (111), (200), (220), and (311) planes, consistent with *fcc* Fm3̅m symmetry. At the same time, Co_0.29_Cu_0.71_ shows pure *fcc*‐Cu with a slightly expanded lattice (a_0_ = 3.6157 Å) as compared to standard Cu (3.6077 Å) [32, 33]. Selective addition of Ir causes peak shifts to lower 2*θ*, Co_0.20_Cu_0.52_Ir_0.28_ shifts toward *fcc*‐Ir due to higher Ir content, whereas Co_0.28_Cu_0.67_Ir_0.05_ remains closer to Cu (enlarged XRD patterns). Lattice parameters derived from Bragg's law increase with Ir: 3.693 Å for Co_0.28_Cu_0.67_Ir_0.05_ and 3.7095 Å for Co_0.20_Cu_0.52_Ir_0.28_, both below that of pure Ir (3.8394 Å), reflecting intermediate alloying (Table S2) [34].

The variation in lattice constants indicates lattice mismatch in CoCuIr NPs induced by selective Ir incorporation. Lattice stress (σ) and strain (ε*_θ_*) are proportional to the lattice spacing under stress (*d_θ_*) and in the absence of stress (*d*_o_). Thus, a properly engineered alloy NP with controlled *a, d_θ_*, and *d*_o_ will directly modify *ε_θ_* and σ, and hence active sites. Hence, alloying CoCu alloy with a small amount of larger Ir atoms is expected to introduce significant lattice distortion in it due to size mismatch, generating dislocations and defects while suppressing atomic diffusion. The Co_0.29_Cu_0.71_ sample shows a minimal mismatch (0.221%) relative to standard *fcc*‐Cu, indicating retention of the Cu lattice. In contrast, Co_0.28_Cu_0.67_Ir_0.05_ exhibits a higher mismatch (2.31%), while Co_0.20_Cu_0.52_Ir_0.28_ shows the highest mismatch (2.74%) (Table S3). This lattice mismatch generates strain and stress, leading to defects and dislocations that affect catalytic activity [26]. Co_0.20_Cu_0.52_Ir_0.28_ exhibits substantial tensile ε*_θ_* and σ, whereas Co_0.29_Cu_0.71_ shows negligible ε*_θ_* relative to *fcc*‐Cu (Tables S4–S6) [35, 36]. Conversely, compressive strain (2.2%) and stress (2.1 N m^−2^) are dominant in Co_0.28_Cu_0.67_Ir_0.05_ relative to Cu. Transmission electron microscopy (TEM) and field emission scanning electron microscopy (FESEM) analyses reveal a morphological transition from smooth, spherical Co_0.29_Cu_0.71_ particles (∼56–60 nm) to smaller, rough‐surfaced particles in Co_0.28_Cu_0.67_Ir_0.05_ (∼9–11 nm) and Co_0.20_Cu_0.52_Ir_0.28_ (∼6–8 nm), attributed to strain‐induced structural evolution (Figure 1c–e, inset histograms; Figures S3–S5).

High‐resolution TEM (HRTEM) images reveal clear lattice fringes with d‐spacings of 0.209 and 0.181 nm for Co_0.29_Cu_0.71_, 0.213 and 0.184 nm for Co_0.28_Cu_0.67_Ir_0.05_, and 0.214 and 0.185 nm for Co_0.20_Cu_0.52_Ir_0.28_, corresponding to the (111) and (200) planes, respectively (Figure 1f–h). These values align well with the XRD results, suggesting the Ir‐induced lattice modification in Co–Cu–Ir alloys. The strain and distortion mapping were performed in conjunction with matching *d*‐spacing trends from the marked HRTEM images using Strain++ software [37]. This provides significant insights into lattice strain and its effect on catalytic activity (Figure 1i–k). Co_0.29_Cu_0.71_ shows a uniform *ε*_xy_ strain map, indicating minimal shear strain and a compressed, strain‐saturated lattice with few distortion‐induced active sites. In contrast, Co_0.28_Cu_0.67_Ir_0.05_ exhibits alternating red‐blue *ε*_xy_ features, signifying localized tensile and compressive strain that can enhance catalytic activity by increasing active sites and tuning the d‐band center [38, 39]. Co_0.20_Cu_0.52_Ir_0.28_ displays the most intense strain contrast, reflecting excessive lattice distortion, which may compromise structural stability and catalytic efficiency (over‐binding of intermediates) despite higher strain. High‐angle annular dark‐field scanning TEM (HAADF–STEM) and FESEM–EDS mapping further indicate the homogeneous distribution of Co, Cu, and Ir in Co_0.28_Cu_0.67_Ir_0.05_ (Figure 1l and Figure S6). Additional HAADF‐STEM elemental mapping and particle‐size distribution analyses across multiple representative regions further indicate the homogeneous elemental distribution and excellent morphological uniformity of the Co_0.28_Cu_0.67_Ir_0.05_ nanostructures (Figure S7). The multipoint N_2_ adsorption/desorption isotherm reveals a higher surface area of 23.54 m^2^ g^−1^ for Co_0.28_Cu_0.67_Ir_0.05,_ with an average pore size of 1.9 nm, compared to the other catalysts (Figure S8). The surface areas of the remaining alloys are provided in Table S7.

**FIGURE 2:**
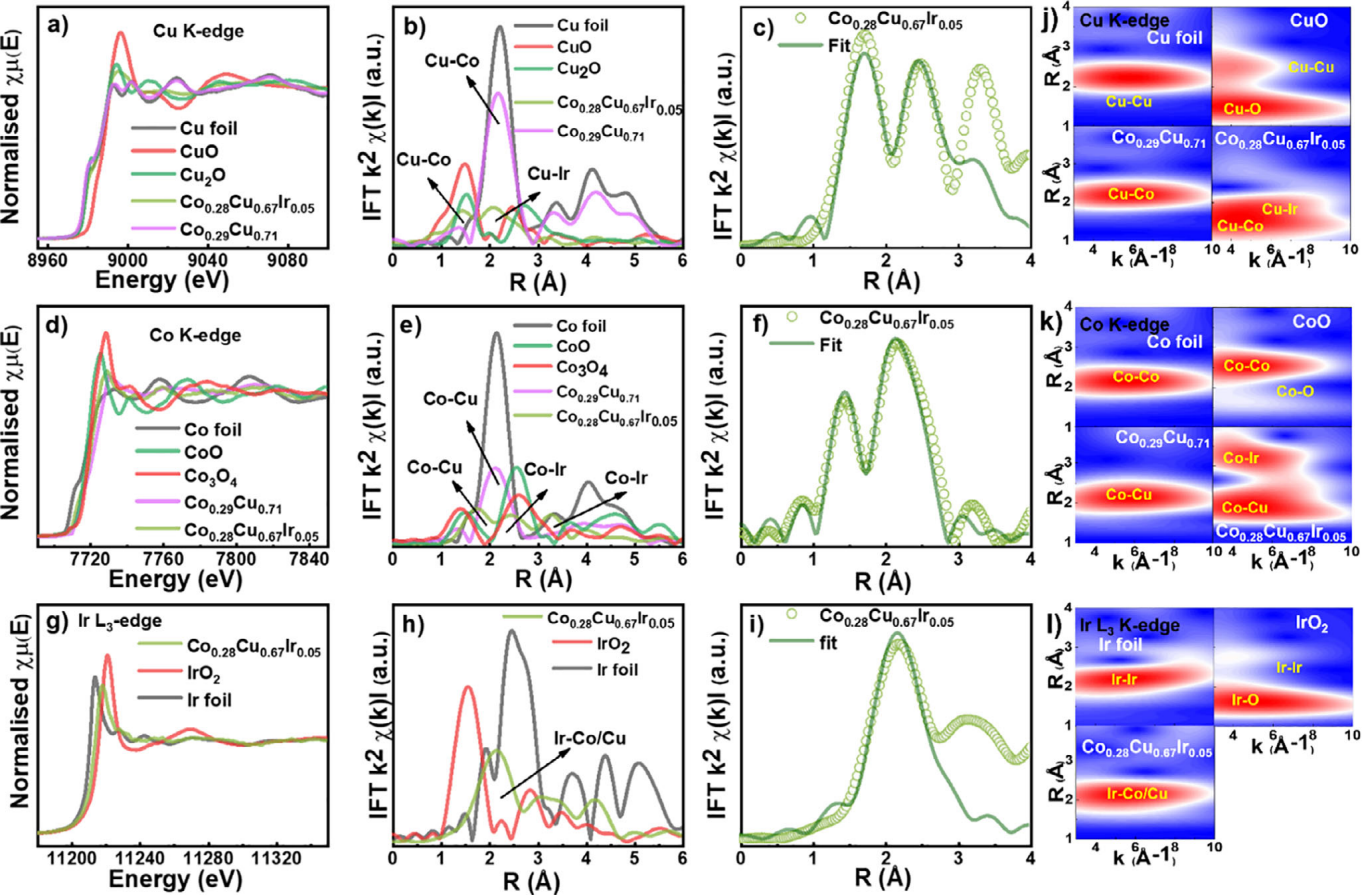
(a,d,g) Cu, Co K‐edge and Ir L_3_‐edge XANES spectra of Co_0.29_Cu_0.71_, Co_0.28_Cu_0.67_Ir_0.05_ and references, respectively. (b,e,h) FT k^2^‐weighted Cu K‐edge, Co K‐edge and Ir L_3_‐edge EXAFS spectra, respectively. (c,f,i) FT‐EXAFS fitting curves plot of Co_0.28_Cu_0.67_Ir_0.05_ at Cu K‐edge, Co K‐edge, and Ir L_3_‐edge, respectively. (j–l) WT‐EXAFS plots Co_0.29_Cu_0.71_, Co_0.28_Cu_0.67_Ir_0.05_, and references as indicated.

X‐ray absorption spectroscopy (XAS) at the Cu K‐, Co K‐, and Ir L_3_‐edges was performed to probe the effects of Ir incorporation on Co–Cu alloys. Cu K‐edge X‐ray absorption near‐edge structure (XANES) shows Co_0.29_Cu_0.71_ has an absorption edge at 8979.88 eV, between Cu^0^ (8978.88 eV) and Cu^2^^+^ (8990.08 eV), while Co_0.28_Cu_0.67_Ir_0.05_ shifts slightly to 8979.70 eV, closer to Cu^+^ (8979.57 eV), indicating mixed Cu^0^/Cu^+^ states with increased Cu^+^ upon Ir doping (Figure 2a) [40]. Co K‐edge XANES (Figure 2d) reveals a shift from 7719.00 eV in Co_0.29_Cu_0.71_ to 7720.64 eV in Co_0.28_Cu_0.67_Ir_0.05_, suggesting a higher Co oxidation state [41]. Ir L_3_‐edge XANES (Figure 2g) shows an edge at 11214.80 eV, near metallic Ir, suggesting Ir^0^ [42]. The corresponding first‐order derivative spectra for Cu, Co, and Ir edges are given in Figures S9a and S10a, and S11a, respectively. These electronic features are fully consistent with the X‐ray photoelectron spectroscopy (XPS) results (discussed in a later section).

Extended X‐ray absorption fine structure (EXAFS) spectroscopy analysis further reveals the structural evolution induced by Ir. As shown in Figure 2b, Cu K‐edge EXAFS fitting for Co_0.29_Cu_0.71_ indicates a dominant Cu–Co coordination shell with a coordination number (CN) of 5.56 and a bond distance of 2.55 Å. In Co_0.28_Cu_0.67_Ir_0.05_, Cu exhibits two coordination paths, Cu–Co (CN = 3.6) and Cu–Ir (CN = 2.0), accompanied by a pronounced contraction of the Cu─Co bond from 2.55 to 1.89 Å, reflecting substantial local lattice distortion [43]. The corresponding FT‐EXAFS fitted curves are shown in Figure 2c and Figure S9c. At the Co K‐edge, Co_0.29_Cu_0.71_ presents Co–Cu coordination (CN = 4.04) with a bond length of 2.50 Å. After Ir doping, Co_0.28_Cu_0.67_Ir_0.05_ shows reduced Co–Cu coordination (CN = 2.12) together with Co–Ir coordination (CN = 1.26), and the Co─Cu bond contracts to 2.12 Å (Figure 2e). The fitted FT curves are shown in Figure 2f and Figure S10c. At the Ir L_3_‐edge (Figure 2h), Ir–Cu (CN = 1.11, 2.11 Å) and Ir–Co (CN = 2.22, 2.56 Å) coordination shells are observed, confirming the successful incorporation of Ir into the alloy matrix (Figure 2i). All fitting parameters and k‐space fittings are summarized in Table S8 and Figures S9–S11.

Wavelet transform (WT) maps (Figure 2j–l) show mixed Cu–Co/Cu–Ir, Co–Cu/Co–Ir, and Ir–M (M = Cu, Co) scattering. The reduction of Cu–Co/Co–Cu CNs, the emergence of Cu─Ir/Co─Ir bonds, and bond contractions demonstrate Ir substitution into the Co–Cu framework, generating compressive lattice strain, consistent with XRD and HRTEM. Overall, XANES, XPS, EXAFS, WT, and XRD indicate that Ir incorporation rebuilds local coordination, induces compressive strain, and subtly modulates Cu and Co electronic states, enhancing seawater electrocatalysis

### 2.2 Electrocatalytic HER and OER Performances in 1.0 m KOH

**FIGURE 3:**
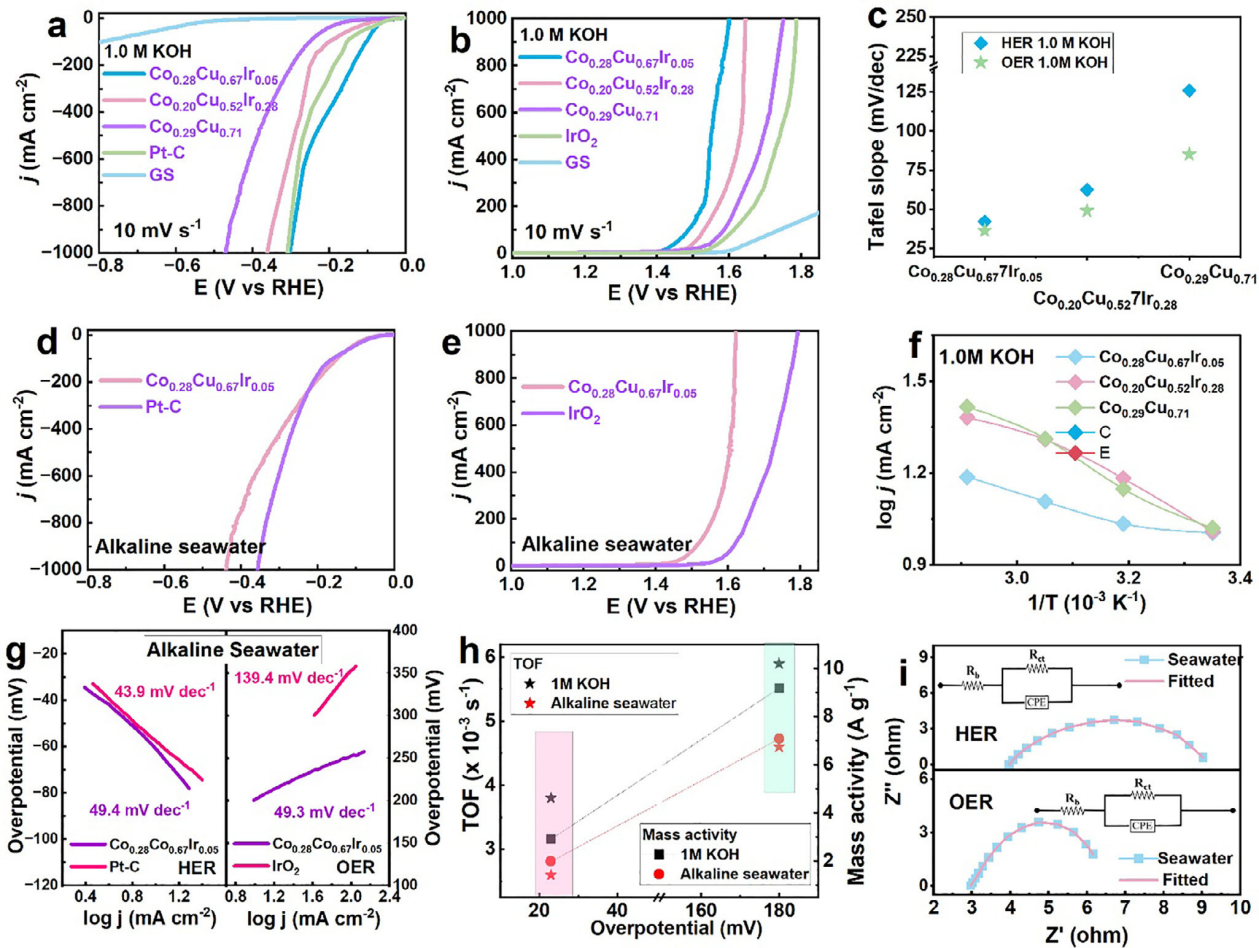
(a,d) *iR*‐corrected LSV curves for HER in 1.0 m KOH and alkaline natural seawater, respectively. (b,e) LSV curves for OER in 1.0 m KOH and alkaline natural seawater, respectively. (c) HER and OER Tafel slope values for the catalysts. (f) Arrhenius plots of log *j* vs. the inverse of temperature measured in KOH. (g) Tafel slopes corresponding to HER and OER of Co_0.28_Cu_0.67_Ir_0.05_ with commercial standards in alkaline seawater, respectively. (h) TOF and Mass activity Co_0.28_Cu_0.67_Ir_0.05_ at HER and OER overpotentials. (i) Nyquist plot along with fitted curve and circuit diagram of Co_0.28_Cu_0.67_Ir_0.05_ in alkaline seawater for HER and OER.

The electrochemical HER and OER performances of the alloy NPs were initially examined using a graphite sheet as the substrate electrode in 1.0 m KOH. The HER linear sweep voltammetry (LSV) plots and the corresponding Tafel plots for each sample are shown in Figure 3a and Figure S12a. Among all, optimized Co_0.28_Cu_0.67_Ir_0.05_ required the lowest *η* of 58 mV to achieve a current density of 10 mA cm^−2^. Moreover, the Co_0.20_Cu_0.52_Ir_0.28_ and Co_0.29_Cu_0.71_ exhibited a Tafel slope of 62.5 and 125.8 mV dec^−1^, which are considerably higher than Co_0.28_Cu_0.67_Ir_0.05_ (42.4 mV dec^−1^) (Figure 3c and Table S9). Based on the Tafel slope, both theoretical and experimental studies earlier propose two possible HER pathways in alkaline media: the Volmer–Tafel mechanism, characterized by a slope close to 30 mV dec^−^^1^, and the Volmer–Heyrovsky mechanism, in which the slope exceeds 40 mV dec^−^^1^ [44]. In the present case, the Co_0.28_Cu_0.67_Ir_0.05_ catalyst follows the Volmer–Heyrovsky pathway [45]. Because HER in alkaline solution requires additional energy to generate protons via water dissociation through cleavage of the H─O─H bond before H^*^ adsorption, the relatively low Tafel slope observed for Co_0.28_Cu_0.67_Ir_0.05_ indicates a high surface coverage of adsorbed hydrogen intermediates and accelerated HER kinetics [46, 47, 48]. Accordingly, the rate‐determining step can be attributed to the coverage and dynamics of adsorbed species, which are strongly influenced by defect sites and Ir‐induced stress–strain effects. These features are corroborated by EXAFS, XPS, and DFT analyses, as discussed in a later section. The high H^*^ coverage on Co_0.28_Cu_0.67_Ir_0.05_ arises from synergistic metal interactions and optimized composition, which tune the electronic structure for optimal intermediate adsorption–desorption and enhanced HER. The OER LSV curve depicts the lowest *η*_10_ = 180 mV for Co_0.28_Cu_0.67_Ir_0.05_ catalyst with a Tafel slope of 36.5 mV dec^−1^, suggesting its faster reaction kinetics as shown in Figure 3b,c and Figure S12b. The *η*_10_ = 298 mV and Tafel slope = 113.3 mV dec^−1^ for commercial IrO_2_. A low Tafel slope value in Co_0.28_Cu_0.67_Ir_0.05_ indicates the higher coverage of M−^*^OH at the catalyst surface and faster OER kinetics [46, 48]. Additionally, the electrocatalyst achieved a high current density of 1000 mA cm^−2^ at low *η* in the alkaline medium (Table S10). Achieving this industrial‐level current density on a graphite sheet is unprecedented compared to Ni or Cu foam substrates.

The electron migration ability, represented by the exchange current density (*i*_ex_) of all the prepared samples, was further analyzed using charge transfer resistance (*R*_ct_), which occurs at the electrode‐electrolyte interface and influences the reaction kinetics. The *R*_ct_ values for both HER and OER were determined using Nyquist plots coupled with circuit fitting (Figures S13 and S14). The observed *R*_ct_ and *i*_ex_ for both water reduction and water oxidation reactions, the bulk resistance (*R*_b_), and other fitting parameters are given in Tables S11 and S12. Among all compositions, Co_0.28_Cu_0.67_Ir_0.05_ has the lowest *R*_ct_ (4.86 Ω for HER and 2.96 Ω for OER) and high *i*_ex_ (29.35 mA cm^−2^ for HER and 24.09 mA cm^−2^ for OER), implying excellent charge transfer capability and electrocatalytic functionality.

The electrochemically active surface area (ECSA) of all alloy NPs was further studied to understand the surface‐active sites responsible for their high catalytic performance (Figure S15a–d). Here, Co_0.28_Cu_0.67_Ir_0.05_ exhibited the highest ECSA (112.25 cm^2^) and roughness factor value (1247), demonstrating the presence of abundant electrochemically active sites (Table S13). The apparent activation energy (*E*_a_) related to sluggish OER was measured in the alkaline electrolyte (Figure S16). The Arrhenius plots for all the catalysts are compared in Figure 3f, which shows that Co_0.28_Cu_0.67_Ir_0.05_ possesses the lowest *E*_a_ (8.02 kJ mol^−1^), implying better thermodynamic feasibility (Table S14). A lower *E*_a_ value further elucidates the ease of the multistep OER at the surface of the optimized Co_0.28_Cu_0.67_Ir_0.05_ due to the availability of numerous defect‐mediated active sites, and maximum coverage by the active species (^*^O, ^*^OH, ^*^OOH). Notably, Co_0.28_Cu_0.67_Ir_0.05_ exhibited the lowest onset potential and the highest ECSA‐normalized current density at a given potential among the four electrocatalysts, indicating its superior intrinsic OER activity (Figure S15e,f). Co_0.28_Cu_0.67_Ir_0.05_ demonstrated the highest turnover frequency (TOF) and mass activity toward both HER and OER (Figure 3h and Table S15). This functionality can be attributed to the presence of optimized Ir and Co, which provide the appropriate energy for the adsorption and subsequent desorption of surface‐active species (^*^OH, ^*^O, and ^*^OOH) during the OER. Furthermore, to examine the possible influence of residual organic ligands on chlorine tolerance, Fourier Transform infrared spectroscopy (FTIR) analysis was performed before and after annealing Co_0.28_Cu_0.67_Ir_0.05_ at 500°C (Figure S17), indicating complete removal of the surface organic layer. The annealed catalyst exhibited overlapping OER activity and chlorine tolerance, indicating that the observed chlorine resistance is intrinsic to the catalyst rather than a consequence of organic‐ligand passivation.

### 2.3 Correlation Between Lattice Strain/Stress and Electrocatalytic Activities

Ir incorporation induces lattice mismatch and strain in the CoCu framework, increasing the mismatch from 0.221% in Co_0.29_Cu_0.71_ to 2.31% in Co_0.28_Cu_0.67_Ir_0.05_, while reducing the particle size from ∼56–60 to ∼9–11 nm. The resulting lattice distortions and defect‐rich regions increase the density of catalytically accessible sites, as evidenced by the highest Brunauer‐Emmett‐Teller (BET) surface area, ECSA, and roughness factor. This optimum strain state (lattice strain ≈ 2.2%, stress ≈ 2.1 N m^−^^2^) delivers the lowest *E*_a_ (8.02 kJ mol^−^^1^), low *R*_ct_ (4.86 Ω for HER and 2.96 Ω for OER), and high *i*_ex_ (29.35 and 24.09 mA cm^−^^2^ for HER and OER, respectively).

Further increasing the lattice mismatch to 2.74% in Co_0.20_Cu_0.52_Ir_0.28_ and reducing the particle size to 6–8 nm causes excessive lattice distortion, strengthening intermediate adsorption and increasing HER and OER Tafel slopes from 42.4 to 62.5 and from 36.5 to 49.1 mV dec^−^^1^, respectively. These results establish a clear correlation between strain magnitude, active‐site density (BET/ECSA), charge‐transfer properties, and HER/OER kinetics, highlighting the importance of an optimal strain state.

To further verify the optimal Ir doping level, we synthesized and evaluated two additional Co–Cu–Ir alloy compositions with different Ir contents, as supported by ICP–MS analysis (Table S16). The electrochemical measurements revealed HER overpotentials of 62 and 103 mV at 10 mA cm^−^^2^ for Co_0.22_Cu_0.70_Ir_0.08_ (lattice strain ≈ 2.1% and stress ≈ 2.04 N m^−^^2^) and Co_0.14_Cu_0.49_Ir_0.37_ (lattice strain ≈ 2.7% and stress ≈ 2.57 N m^−^^2^), respectively, while the corresponding OER overpotentials were 190 and 225 mV (Figure S18). Both compositions exhibit lower activity than the Co_0.28_Cu_0.67_Ir_0.05_ catalyst, indicating that the current minimum Ir incorporation is beneficial for achieving the best bifunctional catalytic performance.

### 2.4 Direct Seawater (Alkaline) Electrolysis and Overall Water‐Splitting Performances

Co_0.28_Cu_0.67_Ir_0.05_ shows the best electrocatalytic performance among the three samples and also matches most reported bifunctional catalysts [5, 15, 16, 19, 20, 21]. Its superior activity makes it the preferred choice for DSE in alkaline conditions. Water reduction LSV and Tafel slope analyses in DSE (Figure 3d,g and Table S17) show that the Co_0.28_Cu_0.67_Ir_0.05_ electrocatalyst requires an *ɳ* of 62 mV to reach 10 mA cm^−^^2^, with a Tafel slope of 49.4 mV dec^−^^1^, slightly higher than Pt/C. For OER, it achieves 10 mA cm^−^^2^ at *ɳ* = 203 mV with a Tafel slope of 49.3 mV dec^−^^1^, outperforming standard IrO_2_ (Figure 3e,g). Nyquist plots (Figure 3i) and fitted equivalent circuits results (Table S18) reveal a low *R*_ct_ of 5.06 Ω for HER and 3.19 Ω for OER, alongside high *i*_ex_ of 28.19 mA cm^−^^2^ (HER) and 22.36 mA cm^−^^2^ (OER), indicating efficient electron transport and superior electrocatalytic activity induced by tensile and compressive lattice strains in Co_0.28_Cu_0.67_Ir_0.05_. In DSE, too, a Volmer–Heyrovsky pathway is followed, as the optimized lattice mismatch‐induced strain creates ample active sites for maximized coverage by the active species.

**FIGURE 4:**
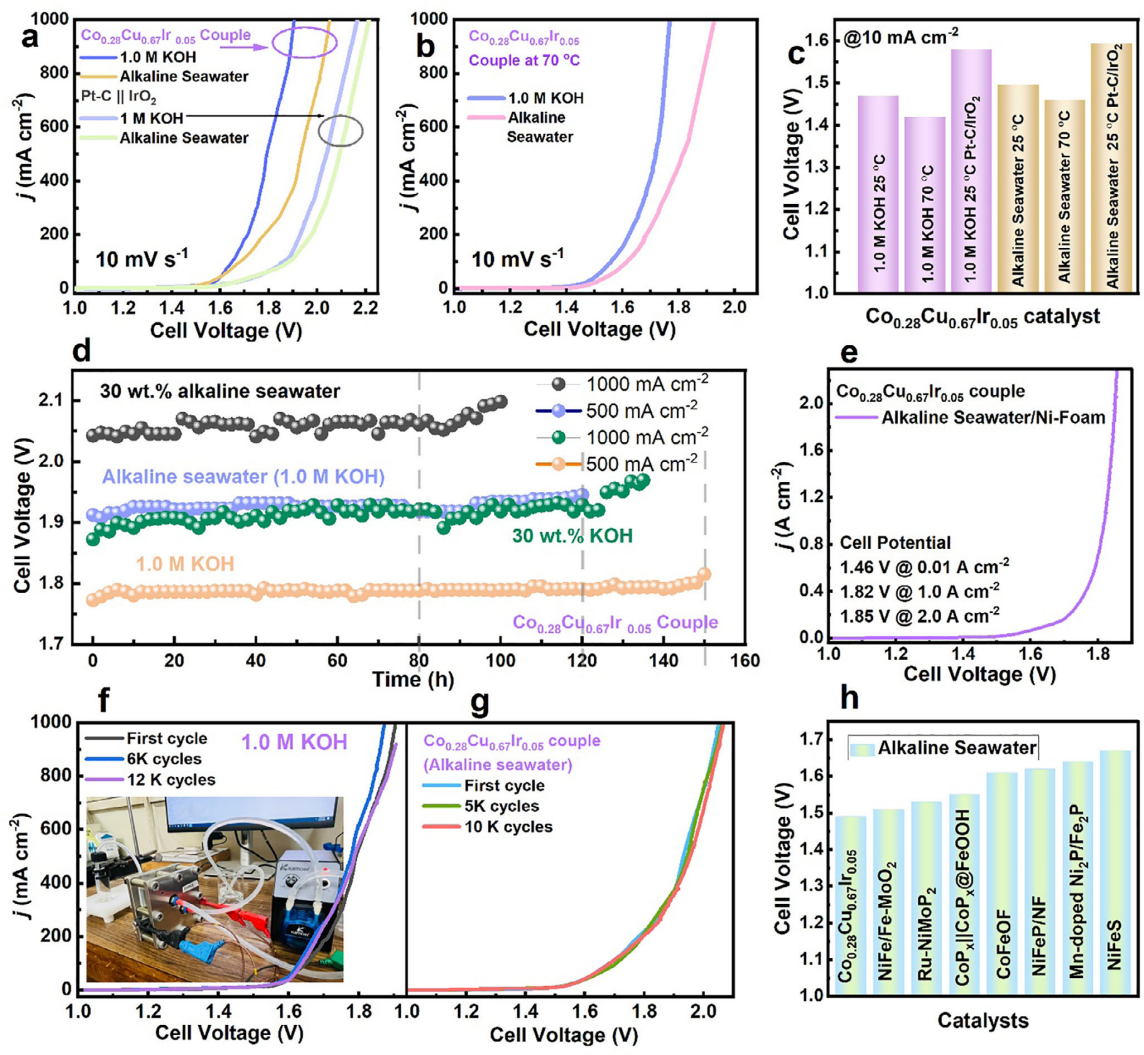
Two‐electrode OWS performance in 1.0 m KOH and alkaline seawater conditions. (a) LSV plots for water electrolysis at 25°C. (b) Potential sweep performance of Co_0.28_Cu_0.67_Ir_0.05_ couple at 70°C. (c) Cell potential comparison of Co_0.28_Cu_0.67_Ir_0.05_ couple catalyst with commercial Pt‐C||IrO_2_. (d) Chronopotentiometry durability analyses of Co_0.28_Cu_0.67_Ir_0.05_ couple under different conditions. (e) Ampere‐level OWS performance for DSE on Ni‐foam by Co_0.28_Cu_0.67_Ir_0.05_. (f,g) LSV cycling stability performance of Co_0.28_Cu_0.67_Ir_0.05_. Inset of (f) OWS picture in an electrolyzer cell. (h) Comparison of DSE OWS cell voltage at 10 mA cm^−2^ with literature reports (Ref. Sl. No 2–8, Table S21).

The outstanding HER and OER performance of Co_0.28_Cu_0.67_Ir_0.05_ nanoparticles arise from lattice strain‐induced defects, small particle size, high surface area, ECSA, and optimal Ir incorporation, which synergistically promote efficient adsorption‐desorption of active sites [49]. This motivates our investigation into overall water electrolysis using a two‐electrode set‐up. Figure 4a,c shows that the Co_0.28_Cu_0.67_Ir_0.05_ couple exhibits enhanced performance at 25°C, achieving 10 mA cm^−2^ current density at low cell voltages of 1.47 and 1.49 V in 1.0 m KOH and alkaline seawater, respectively. Notably, the Co_0.28_Cu_0.67_Ir_0.05_ couple exhibits a lower potential than the standard Pt‐C||IrO_2_ system, as well as high current density (1000 mA cm^−2^) at low cell voltage (2.05 V) in DSE (Table S19). To explore the potential for industrial applications, we measured cell voltage at 70°C (Figure 4b). Here, the couple affords cell potentials of 1.42 and 1.46 V in 1.0 m KOH and DSE, respectively, at 10 mA cm^−2^. This reduction in cell potential is attributed to the increased kinetic energy of the electrolytic conditions at higher temperatures. The electrocatalysts demonstrated excellent chronopotentiometry durability at a high current density of 500 mA cm^−2^, exhibiting stability for measured 150 and 120 h in 1.0 m KOH and DSE conditions, respectively, as shown in Figure 4d. To quantitatively assess the catalyst's long‐term durability, the voltage decay rate was calculated from chronopotentiometric measurements as ΔV/Δt. The Co_0.28_Cu_0.67_Ir_0.05_ electrode exhibited low voltage decay rates of 0.286 mV h^−^^1^ and 0.275 mV h^−^^1^ during continuous operation in 1.0 m KOH and in alkaline seawater electrolyte, respectively, indicating excellent electrochemical stability.

All our primary studies were done on a graphite sheet substrate. We have now tested our material on the Ni‐foam (NF) substrate. Here, the performance of our catalyst is benchmarked against nearly all catalysts, reaching a current density of 2.3 A cm^−2^ at low cell voltage (∼1.85 V) in untreated seawater, as shown in Figure 4e (Table S19). A detailed comparison with previously reported electrocatalysts is summarized in Tables S20 and S21, indicating the superior functionality of our present catalyst for DSE. We conducted LSV cycling stability tests for the Co_0.28_Cu_0.67_Ir_0.05_ couple. The maximum measured cycles in KOH and alkaline seawater were 12 000 and 10 000, respectively, where the first and the final LSV curves are almost overlapped (Figure 4f,g). A photo of the two‐electrode commercial electrolyzer cell is shown in the inset (Figure 4f).

To evaluate practical applicability, overall water‐splitting performance was investigated in concentrated alkaline and alkaline seawater electrolytes, as well as at higher current density. The Co_0.28_Cu_0.67_Ir_0.05_ electrolyzer required cell voltages of 1.47, 1.52, 1.49, and 1.55 V to achieve 10 mA cm^−^^2^ in 1.0 m KOH, 30 wt.% KOH, alkaline seawater, and 30 wt.% alkaline seawater, respectively (Figures S19 and S20). Cycling tests demonstrated excellent durability over 10 000 cycles in 30 wt.% KOH and 8000 cycles in 30 wt.% alkaline seawater with negligible degradation (Figures S19 and S20). Furthermore, stable operation at 1.0 A cm^−^^2^ was maintained for 135 h and 100 h in 30 wt.% KOH and 30 wt.% alkaline seawater, respectively, with only minor voltage decay (Figure 4d). The Faradaic efficiencies of the Co_0.28_Cu_0.67_Ir_0.05_ catalyst were evaluated at 100 mA cm^−^^2^ for 120 min using the inverted burette method and gas chromatography. The catalyst delivered Faradaic efficiencies of 96.34% for HER (Figure S21a) and 95.12% for OER (Figure S21b), demonstrating efficient charge utilization and high selectivity for alkaline seawater electrolysis. Finally, the performance of our catalyst is compared with some of the robust and benchmarked electrocatalysts in the literature for DSE at the same current density of 10 mA cm^−2^ (Figure 4h and Table S21). These comparisons highlight the competitive performance of our material among recently reported catalysts.

### 2.5 Mechanistic Insights from DFT, Quasi‐Operando Raman, and Ex Situ Post‐Catalytic XPS and XRD Analyses

**FIGURE 5:**
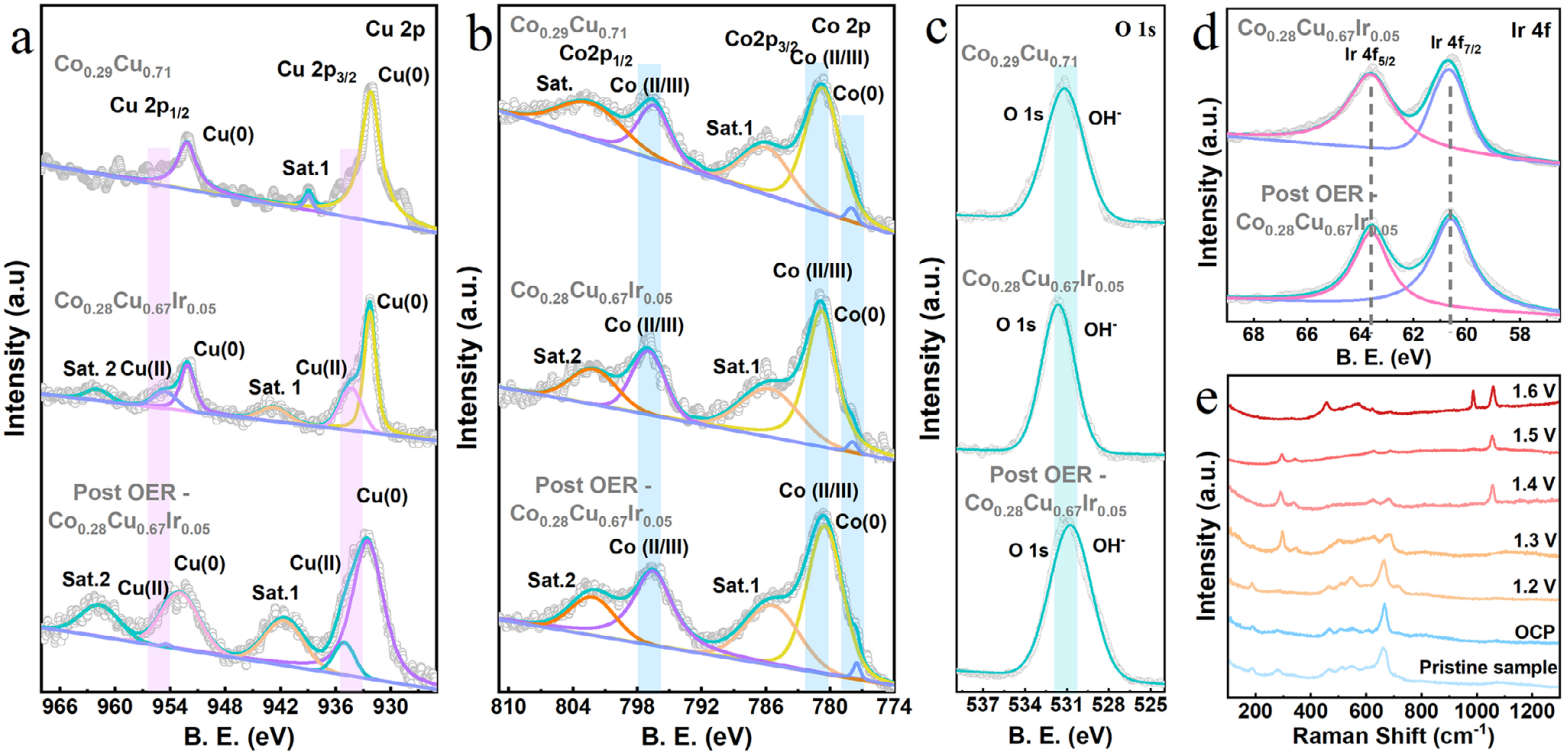
High‐resolution XPS spectra of Co_0.29_Cu_0.71_, fresh Co_0.28_Cu_0.67_Ir_0.05_, and post‐OER Co_0.28_Cu_0.67_Ir_0.05_ (after OWS in alkaline seawater) for (a) Cu 2p, (b) Co 2p, (c) O 1s, and (d) Ir 4f. (e) Quasi‐operando Raman spectra under anodic conditions with increasing applied potential.

All electrocatalytic metrics, including ECSA and roughness, low Tafel slope, and high i_e__x_, unambiguously indicate a high density of active sites on Co_0.28_Cu_0.67_Ir_0.05_, arising from controlled lattice distortion and strain that enable efficient adsorption of reaction intermediates for both HER and OER. To elucidate the surface chemical states, XPS measurements were conducted on Co_0.29_Cu_0.71_, fresh Co_0.28_Cu_0.67_Ir_0.05_, and post‐OER Co_0.28_Cu_0.67_Ir_0.05_ after 120 h of DSE. Survey spectra indicate the presence of Cu, Co, O, and Ir in all samples (Figure S22). High‐resolution Cu 2p spectra (Figure 5a) display Cu^0^ 2p_3/2_ peaks at 932.0 eV (Co_0.29_Cu_0.71_), 932.3 eV (Co_0.28_Cu_0.67_Ir_0.05_), and 932.7 eV (post‐OER Co_0.28_Cu_0.67_Ir_0.05_), along with Cu^2^^+^ features at 934.4 eV for the fresh and 934.9 eV for the post‐OER Ir‐containing catalyst (Δ ≈ 20.5 eV) [47, 50]. In the Co 2p_3/2_ region (Figure 5b), weak Co^0^ shoulders appear at 777.9, 777.7, and 777.6 eV, accompanied by dominant Co^2^^+^/Co^3^^+^ peaks at 780.7, 780.8, and 780.5 eV for Co_0.29_Cu_0.71_, fresh and post‐OER Co_0.28_Cu_0.67_Ir_0.05_, respectively (Δ ≈ 15.7 eV) [51]. The presence of Cu(II) and Co(II/III) species on both fresh and spent catalyst surfaces suggests that the fresh CoCuIr undergoes only superficial oxidation upon air exposure. As shown in Figure 5a,b, the relative amounts of oxide species and metallic (0) states remain nearly unchanged after prolonged electrolysis. O 1s spectra reveal peaks at 531.2, 531.6, and 530.9 eV, indicating hydroxide formation (Figure 5c). Notably, Ir 4f spectra retain metallic Ir^0^ features at 60.6/63.6 eV (4f_7/2_/4f_5/2_) before and after OER (Figure 5d), underscoring the noble character of Ir supporting the XANES result, and its stabilizing role. The systematic shifts between Co_0.29_Cu_0.71_ and Co_0.28_Cu_0.67_Ir_0.05_ highlight Ir‐induced lattice strain and electronic modulation, which optimize ^*^O/^*^OH/^*^OOH adsorption–desorption kinetics on stress‐generated active sites, thereby balancing activity and durability during the uphill OER.

To elucidate the structural evolution of Co_0.28_Cu_0.67_Ir_0.05_ during alkaline seawater OER, quasi‐operando Raman spectroscopy was performed at progressively increasing anodic potentials (Figure 5e). The pristine catalyst exhibits Raman features at ∼191 cm^−1^ (cobalt oxide), 450–470 cm^−1^ (Co(OH)_2_ *E*_g_), 510–520 cm^−1^ (Co(OH)_2_ *A*_1g_), 590–610 cm^−1^ (CoOOH), and 658–670 cm^−1^ (native oxide/hydroxide/oxyhydroxide surface layer) [52, 53]. These bands remain largely unchanged at open circuit and 1.2 V, indicating limited surface hydroxylation. At 1.3–1.4 V, Co–O/Co(OH)_2_ features diminish, while CoOOH‐related bands at 503 and 682 cm^−1^ become stronger [54], together with a band at 620 cm^−1^ attributable to Co─O vibrations involving octahedrally coordinated Co^3+^ rather than irreversible phase transformation [52]. The CuO *A*_g_ and *B*_g_ modes at ∼290–299 and 340–345 cm^−1^ become prominent at 1.3–1.5 V [55]. Weak bands at 1000–1100 cm^−1^ at 1.5–1.6 V are assigned to adsorbed oxygenated species and/or electrolyte‐derived oxyanions. The persistence/reappearance of low‐frequency features at higher potential indicates retention of the alloy framework during OER.

This structural stability is consistent with ex situ XPS after 120 h, which shows nearly unchanged Cu 2p, Co 2p, O 1s, and Ir 4f spectra, including preserved Co^2+^/Co^3+^ and Co^0^ ratios and metallic Ir^0^, with no significant growth of oxide/hydroxide/oxyhydroxide components. Thus, enhanced OER activity is primarily expected to arise from defect sites, reversible electronic modulation of the native oxide/hydroxide surface, and optimized adsorption of oxygenated intermediates, rather than from the formation of a new oxyhydroxide phase. Nevertheless, limited additional surface reconstruction cannot be entirely ruled out. No Raman signatures of chlorine‐containing species were detected, suggesting suppressed chlorine oxidation and high OER selectivity in alkaline seawater.

This direct evidence of adsorption and activation of ^*^O/^*^OH/^*^OOH species supports an adsorbate evolution mechanism (AEM) in which oxygenated intermediates are sequentially bound to the catalyst surface during OER. Thus, it is evident that the pre‐existing hydroxides and oxyhydroxide layers from the initial stage (fresh sample) selectively resist chloride‐induced corrosion and structural degradation. To validate this, the survey XPS spectra are enlarged in the 0–370 eV region (Figure S22). Surprisingly, we do not find any peak at 199–201 eV related to Cl 2p. This extraordinary Cl^−^ tolerance can be ascribed to self‐driven or auto‐generated Co^2+^/Co^3+^ and Cu^2+^ ions at the surface, as evidenced by XPS. Based on hard‐soft acid‐base (HSAB) theory, Co^2+^/Co^3+^/Cu^2+^ are hard Lewis acids. They prefer to bond with hard Lewis bases, OH^−,^ and have very little affinity for softer or borderline bases Cl^−^ [56]. Hence, lattice strain‐induced defects contribute to efficient HER+OER, and surface hard Lewis acidic sites simultaneously prevent Cl^−^ adsorption. Additionally, tiny peaks at 347 and 49.8 eV related to Ca 2p and Mg 2p, respectively, are observed, which are attributed to minor Mg(OH)_2_ and Ca(OH)_2_ phases. These additional phases are observed in the XRD pattern of the post‐DSE sample in minute percentages (Figure S23). However, no peaks are detected related to Cu(OH)_2_, Co(OH)_2_, and CoOOH, corroborating adsorption and activation of ^*^O/^*^OH/^*^OOH species only on the defect sites. Post‐DSE TEM and FESEM analyses show a slight reduction in particle size, whereas the overall morphology remains essentially unchanged compared to the fresh catalyst. (Figure S24). Furthermore, as shown in Figure S25, TEM‐EDS elemental mapping indicates the uniform distribution of Co, Cu, and Ir in the Co_0.28_Cu_0.67_Ir_0.05_ anode after 50 h of chronopotentiometric electrolysis at 2.0 A cm^−2^, demonstrating the preservation of its elemental distribution. The corresponding energy dispersive X‐ray spectroscopy (EDS/EDX) spectrum (Figure S26) suggested the presence of the constituent elements, while quantitative EDX surface analysis (Table S22) shows no detectable Cl (N.D.), indicating negligible chloride accumulation on the catalyst surface after prolonged electrolysis.

The possible formation of any chlorine‐derived byproducts including Cl_2_ gas during alkaline seawater electrolysis was further investigated colorimetrically [57]. Methyl orange (MO) was employed as a colorimetric probe to detect ClO^−^. As shown in Figure S27a, increasing ClO^−^ concentrations (1–100 mm) caused progressive bleaching of MO and a marked decrease in the characteristic absorption band at 510 nm, indicating the method's sensitivity. In contrast, the UV‐Vis spectrum of the electrolyte after 1 h electrolysis at 100 mA cm^−^^2^ overlapped with that of pristine MO, and no visible color change was observed (Figure S27b), indicating negligible ClO^−^ formation. Furthermore, gas collected from the anode was passed through a MO solution, which showed no spectral or visual changes before and after electrolysis (Figure S27c,d). This suggested that chlorine‐derived oxidizing species were below the detection limit or absent. These results suggest highly selective oxygen evolution with effective suppression of chlorine evolution.

Chronopotentiometry tests at 1.0 A cm^−^^2^ using NF as the substrate showed that performance degradation after 120 h in alkaline seawater (Figure S28) mainly resulted from catalyst layer detachment caused by continuous gas evolution and corrosion of the NF support. ICP–MS analysis (Table S23) revealed reduced Cu and Ir contents after testing, indicating partial dissolution or redistribution of active species during prolonged operation. ICP–MS analyses on the spent sample detected only trace Ca species and negligible Mg accumulation, consistent with TEM‐EDAX and suggesting minimal inorganic scaling and that Mg/Ca‐induced passivation was not a significant deactivation pathway. Chlorine polarization measurements (Figure S29) showed that Co_0.28_Cu_0.67_Ir_0.05_ possesses superior chloride corrosion resistance compared with bare NF, highlighting the role of Ir in improving electrode durability. The membrane retained its integrity and transparency, with only minor surface deposits after long‐term operation (Figure S30).

**FIGURE 6:**
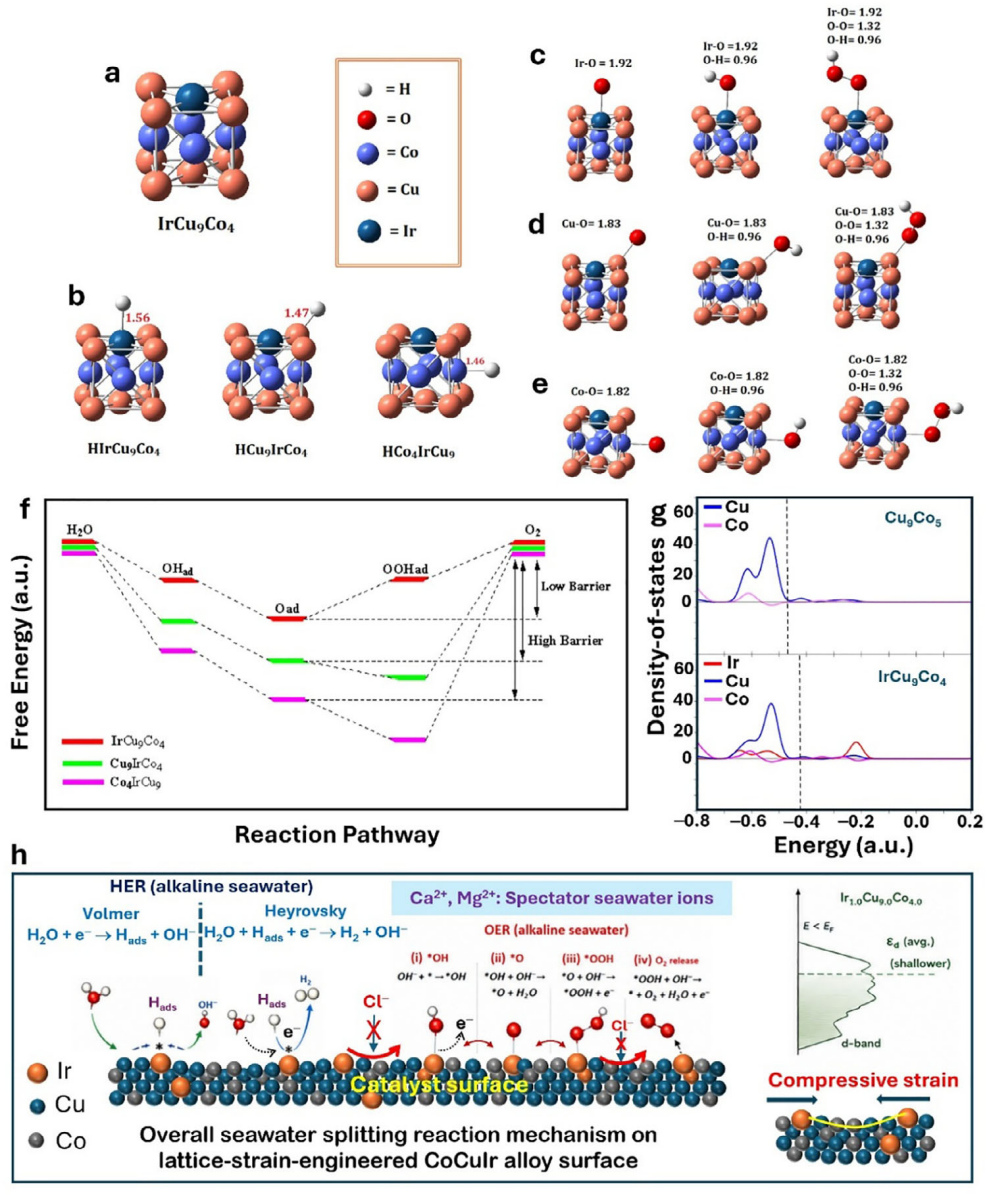
(a) Minimum energy structures of the Co_4.0_Cu_9.0_Ir_1.0_ unit cell. (b) H‐adsorbed structures of Co_4.0_Cu_9.0_Ir_1.0_ unit. Bond lengths are in Å. (c–e) Optimized geometries of the O‐bound intermediates with possible absorption sites for OER steps on Co_4.0_Cu_9.0_Ir_1.0_ unit cell. Bond lengths are given in Å. (f) Schematic illustration for the OER on Co_4.0_Cu_9.0_Ir_1.0_ unit cell. (g) Partial density of states plots of bimetallic Co_5.0_Cu_9.0_ and Co_4.0_Cu_9.0_Ir_1.0_ unit cell showing more favorable active site for OER at Ir in comparison to Cu and Co. (h) Schematic diagram showing the overall seawater splitting reaction mechanism on the lattice‐strain‐engineered CoCuIr alloy surface and d‐band modulation.

For our theoretical investigation, we considered a model *fcc* unit cell with the composition Co_4.0_Cu_9.0_Ir_1.0_ (approx. equivalent to Co_0.28_Cu_0.67_Ir_0.05_ in a 14‐atom *fcc* unit cell), and the geometries were optimized as described in the SI file. Moreover, we made two additional sets of models in which the unit cell is coated with hydroxide/oxyhydroxide species at the Co and Cu sites, as XPS results identified oxidized Co^2+^/Co^3+^ and Cu^2+^ species on the surface of both fresh and spent catalysts. For Co_0.29_Cu_0.71_, the model *fcc* unit cell is taken as Co_5.0_Cu_9.0_. Notably, for Co_0.20_Cu_0.52_Ir_0.28_ (Co_3.0_Cu_7.0_Ir_4.0_), the structure breaks down due to overburden by heavier Ir metals. This corroborates our earlier statement that excessive strain can also cause structural instability, electronic disorder, or over‐binding of intermediates, which can actually reduce catalytic efficiency. The minimum energy structures of pristine Co_4.0_Cu_9.0_Ir_1.0_, and with surface OH^−^ and OOH^−^ species, are depicted in Figure 6a and Figures S31a and S32a, respectively. The optimized geometries of the H‐adsorbed Co_4.0_Cu_9.0_Ir_1.0_ unit cell, which is taken into consideration for the HER activity, are depicted in Figure 6b. To evaluate the HER activity, we attached H atoms at different locations, such as at Ir, Cu, and Co. The computed Ir–H, Cu–H, and Co–H distances in the H‐adsorbed Co_4.0_Cu_9.0_Ir_1.0_ unit cell are 1.56, 1.47, and 1.46 Å, respectively.

To investigate the origin of OER and HER activity in the Co_4.0_Cu_9.0_Ir_1.0_ unit cell, DFT calculations were performed. Previous studies have shown that HER activity can be reliably predicted by evaluating the hydrogen adsorption free energy *H*(*G*(H*)) on different catalytic materials [58]. A catalyst with a near‐zero adsorption free energy (ΔG(H*) ≈ 0) is generally considered optimal for HER [59]. The calculated ΔG(H*) values for the Co_4.0_Cu_9.0_Ir_1.0_ and the hydroxide/oxyhydroxide‐coated unit cell are presented in Table 1. Although the model systems used for DFT calculations are smaller and more simplified than those observed experimentally, they capture the essential effects governing catalytic behavior by including hydroxide/oxyhydroxide species at the surface during the calculations. Table 1 shows that the Ir center has the most favorable H‐atom adsorption, with the lowest ΔG(H^*^) values of 0.30 and 0.31 eV, whereas the Co center exhibits the least favorable adsorption, with the highest ΔG(H^*^) values of 1.52 and 1.56 eV, without and with surface hydroxide/oxyhydroxide layers, respectively. Notably, the ΔG values are nearly identical for the hydroxide‐ and oxyhydroxide‐coated samples, with differences emerging only beyond the third decimal place. Therefore, the effects of OH^−^ and OOH^−^ in the reactants and products remain almost the same, resulting in very similar ΔG values. These results suggest that the Ir center is the most favorable active site for HER, followed by the Cu center in both cases.

**TABLE 1:** Adsorption free energy *H*[Δ*G*(H*)] (in eV) for hetero‐nanostructures Co_4.0_Cu_9.0_Ir_1.0_ unit cell.

Structure	Adsorption Site	Δ*G*(H*)
Ir_1.0_Cu_9.0_Co_4.0_	Ir	0.30
	Cu	1.13
	Co	1.52
OH^−^/OOH^−^‐coated Ir_1.0_Cu_9.0_Co_4.0_	Ir	0.31
	Cu	1.21
	Co	1.56

For efficient OER catalysis, intermediates must not bind too strongly, as excessive adsorption raises the desorption barrier [48]. To assess this, the free energies of oxygen‐based intermediates (^*^O, ^*^OH, and ^*^OOH) were calculated for individual Ir, Cu, and Co centers on the pristine sample (Figure 6c–e) and with a hydroxide/oxyhydroxide layer‐coating (Figures S31b–e and S32b–e). The adsorption energy profile (Figure 6f and Figures S31f and S32f) reveals that the Ir center in pristine Co_4.0_Cu_9.0_Ir_1.0_ and hydroxide/oxyhydroxide‐coated Co_4.0_Cu_9.0_Ir_1.0_ exhibits the lowest adsorption energies and thus the lowest energy barrier, making it the most effective active site for OER. This weaker Ir─O interaction arises from the larger atomic radius of Ir compared to Co and Cu. In contrast, Co and Cu centers bind oxygen intermediates more strongly, leading to higher barriers. To further probe the origin of this enhanced activity, the PDOS of bimetallic Co_5.0_Cu_9.0_ and trimetallic Co_4.0_Cu_9.0_Ir_1.0_ unit cells were calculated and compared in Figure 6g. The PDOS analysis shows that the Fermi level of Co_4.0_Cu_9.0_Ir_1.0_ shifts to the right relative to Co_5.0_Cu_9.0_, with Ir contributing alongside Cu at the Fermi level. This shift, induced by Ir incorporation, enhances the electronic activity toward OER. Moreover, the PDOS of hydroxide‐ and oxyhydroxide‐coated Co_4.0_Cu_9.0_Ir_1.0_ unit cells were calculated and presented in Figures S31g and S32g, respectively. Compared to the pristine and hydroxide‐coated Co_4.0_Cu_9.0_Ir_1.0_ unit cells, the oxyhydroxide‐coated Co_4.0_Cu_9.0_Ir_1.0_ unit cell performs slightly better, as evident from the little shifting of the Fermi level (Figure S32g). However, this right‐shifting is less prominent than that reported in the literature for true, active, and stable phase cobalt‐based (CoOOH) electrocatalysts for OER in an alkaline medium [53, 54]. DFT results corroborate the quasi‐operando Raman findings, indicating that OER activity primarily originates from intrinsic defects and/or reversible electronic modulation of the native oxide/hydroxide surface, rather than a new oxyhydroxide phase, although limited surface reconstruction cannot be excluded.

Overall, these results demonstrate that Co_4.0_Cu_9.0_Ir_1.0_ provides highly active catalytic sites, rendering the unit cell an efficient and robust bifunctional catalyst for both HER and OER, thereby promoting efficient overall water splitting. Meanwhile, Ir also induced stress in the CuCu *fcc* lattice through lattice mismatch, shortened the bonds, and created sufficient active sites for enhanced DSE, as suggested by EXAFS, stress‐strain, and XPS studies. Corroborating all the experimental and computational evidence, the OWS reaction mechanism that occurs at the surface of the lattice‐strain‐engineered CoCuIr alloy is shown in Figure 6h. The HER occurs at the compressed strain‐induced active sites (Ir/Cu), while OER occurs at the Ir/Co sites. The partial oxidation of the surface layer of CoCuIr and the formation of minor Co/Cu‐hydroxide and oxyhydroxide layers from the beginning (fresh sample) resists the chloride‐induced corrosion and structural degradation. Moreover, Ir enhances seawater electrolysis by stabilizing OER intermediates (^*^OH, ^*^O, ^*^OOH) more strongly than chloride intermediates (^*^Cl). This preferential adsorption pathway thermodynamically favors oxygen evolution over chlorine formation, resulting in high OER selectivity in DSE [60].

Finally, the optimized catalyst demonstrates promising performance by sustaining industrially relevant current densities above 2.0 A cm^−^^2^ at 1.85 V (NF substrate) and ∼1.0 A cm^−^^2^ (graphite sheet substrate) under alkaline seawater conditions. It also maintained long stable operation for >120 h at 1.0 A cm^−^^2^ in alkaline seawater and in industrial condition (30 wt.% alkaline seawater). The use of earth‐abundant Co and Cu significantly lowers material costs relative to conventional noble‐metal electrocatalysts. Although Ir is scarce and expensive, its loading is limited to only 5 at.%, reducing precious metal usage while retaining high bifunctional activity for both HER and OER. Nevertheless, further optimization of Ir loading, catalyst utilization, and scalable synthesis routes will be essential to improve economic viability for large‐scale deployment.

## 3 Conclusions

In conclusion, we demonstrated that controlled incorporation of a larger Ir atom into a CoCu alloy effectively tailored the local coordination environment and electronic structure, resulting in compressive lattice strain and highly active catalytic sites for DSE. The marked decrease in Cu–Co/Co–Cu coordination, together with the formation of Cu─Ir and Co─Ir bonds and accompanying bond contraction, evidences strain‐driven structural reorganization and particle size reduction. The optimized Co_0.28_Cu_0.67_Ir_0.05_ catalyst benefits from these combined effects, which collectively maximize surface energy and defect density, enabling synergistic enhancement of bifunctional electrocatalytic activity. As a result, the catalyst delivers low operating voltages to reach a benchmark current density of 2.0 A cm^−2^ at ambient conditions. DFT calculations corroborate these findings by revealing optimized *H*(*G*(H*)) for accelerated HER kinetics alongside reduced OER adsorption energies and kinetic barriers. Pre‐existing Cu^2+^/Co^2+^/Co^3+^ hard Lewis acid sites formed through surface oxidation of CoCuIr NPs weaken Cl^−^ adsorption, effectively suppressing chloride‐induced corrosion. This reduced structural degradation while preferentially stabilizing OER intermediates over Cl^−^, thereby enabling highly selective oxygen evolution in seawater electrolysis. No noticeable surface structural changes are observed under quasi‐operando Raman spectroscopy measurement. This work demonstrates that lattice‐strain engineering with nanoscale control, including particle‐size reduction, increased surface area, abundant active sites, and electronic modulation in trimetallic alloys, is an effective strategy for efficient and durable seawater electrolysis toward sustainable green hydrogen production.

## Author Contributions

S.D. directed the project, provided funding. A.K. performed materials synthesis, characterizations and electrochemical experiments. A.Y. analyzed the results of strain mapping, electrochemical experiments. S.L. performed XAS data analysis. P.J.T. and A.K.G. carried out and analyzed the DFT calculations. All authors wrote the paper and agreed to the submitted version of the manuscript.

## Conflicts of Interest

The authors declare no conflicts of interest.

## Supporting information




**Supporting File**: advs77531‐sup‐0001‐SuppMat.pdf.

## Data Availability

The data that support the findings of this study are available from the corresponding author upon reasonable request.
