# Supplementary material for: Controlled Lattice Distortion and Lattice‐Strain‐Engineered CoCuIr Alloy toward Preferential Chlorine Tolerant Direct Seawater Electrolysis
Source: Adv Sci (Weinh). 2026 Aug 31:e77531. Online ahead of print. doi: 10.1002/advs.77531 (PMC13530019; doi:10.1002/advs.77531)
Supplement: Supplementary file 1 [file ADVS-9999-e77531-s001.pdf]

Supporting Information

Controlled Lattice Distortion and Lattice-Strain-Engineered CoCuIr Alloy towards Preferential Chlorine Tolerant Direct Seawater Electrolysis

Ankur Kumar,[a,#] Abhinav Yadav,[a,#] Saloni Latiyan,[a] Palash J. Thakuria,[b] Ankur K. Guha,[b] Sasanka Deka*,[a]

[a]Department of Chemistry, University of Delhi, North Campus, Delhi, 110007, India

[b]Advanced Computational Chemistry Centre, Cotton University, Panbazar, Guwahati, 781001, India

*email: sdeka@chemistry.du.ac.in

[#]Both A.K. and A.Y. contributed equally to the work.

Materials and reagents

Iridium(III) chloride hydrate [$IrCl_3.xH_2O$, 99.9%], cobalt acetate tetrahydrate [$Co(CH_3COO)_2.4H_2O$, 98.0 %], oleic acid [OLAC, $C_{18}H_{34}O_2$ 90%], oleylamine [OLAM, $C_{18}H_{37}N$, 70%], nafion [5% in a mixture of lower aliphatic alcohol and water], platinum carbon [Pt/C, 20 wt.%] and iridium oxide [IrO_2, 99.9%] were purchased from Sigma-Aldrich, USA. Copper acetate monohydrate [$Cu(CH_3COO)_2.H_2O$, 99 %], maleic Acid [$C_4H_4O_4$, 99%], and sulfuric acid [H_2SO_4, 98 %] were obtained from Thomas-Baker, India. Potassium hydroxide [KOH] was acquired from SRL, India. The chemicals were employed as received, without any further modification.

Preparation of CoCuIr alloy nanostructures:

The development of $Co_{0.28}Cu_{0.67}Ir_{0.05}$ nanostructures was executed through a colloidal synthesis method. Firstly, $Co(ac)_2.4H_2O$ (0.67 mmol), $Cu(ac)_2.H_2O$ (0.62 mmol), $IrCl_3.xH_2O$ (0.21 mmol), maleic acid (0.9 mmol), OLAM (15 mL), and OLAC (15 mL) were taken in a 100 mL three-neck round-bottom flask. The flask was then connected with a condenser, a thermocouple attached to the temperature controller and a magnetic stirrer in a Schlenk line. The reaction mixture was subjected to degassing at 100 °C for 45 min. Thereafter, the reaction mixture was maintained with a constant N_2 flow for an additional 90 min at 290 °C. After completion of the reaction, the reaction mixture was allowed to reach room temperature under a continuous nitrogen atmosphere. Toluene was added to the solution, and the mixture was precipitated with

ethanol. Finally, the mixture was subjected to centrifugation 5 times at 5000 rpm for 5 min, utilizing the same solvents, and dried at 60 °C for 24 hours. A similar procedure was utilized for the other compositions ($Co_{0.20}Cu_{0.52}Ir_{0.28}$ and $Co_{0.29}Cu_{0.71}$) with a different molar concentration of metal salt precursor.

<u>Control Synthesis</u>

Preparation of $Co_{0.28}Cu_{0.67}Ir_{0.05}$-200 °C nanostructures: A similar synthetic method was deployed for the controlled synthesis of $Co_{0.28}Cu_{0.67}Ir_{0.05}$-200 °C as for CoCuIr nanostructures, but with the temperature of 200 °C under inert conditions for 90 min.

Preparation of $Co_{0.28}Cu_{0.67}Ir_{0.05}$-No OA nanostructures: The controlled synthesis of $Co_{0.28}Cu_{0.67}Ir_{0.05}$-No OA was implemented in a similar process as sustained for CoCuIr nanostructures, but in the absence of OLAC.

Preparation of $Co_{0.28}Cu_{0.67}Ir_{0.05}$-No MA nanostructures: The controlled synthesis of $Co_{0.28}Cu_{0.67}Ir_{0.05}$-No MA was achieved using a similar technique as utilized for CoCuIr nanostructures without the addition of maleic acid.

Experimental Procedure for ClO^- Detection Using Methyl Orange

To evaluate the formation of hypochlorite (ClO^-) during alkaline seawater electrolysis, methyl orange (MO) was employed as a colorimetric probe. A stock MO solution (20 mg L^{-1}) was prepared in deionized water. Aliquots of the MO solution were prepared and 1, 10, 50 and 100 mM standard NaClO solutions were added separately and allowed to react for 5 min. The resulting UV–Vis spectra were recorded in the wavelength range of 450–575 nm to establish the characteristic bleaching behavior of MO in the presence of ClO^-.

For electrolysis testing, alkaline seawater electrolysis was conducted at a constant current density of 100 mA cm^{-2} for 1 h using the $Co_{0.28}Cu_{0.67}Ir_{0.05}$ electrode. After electrolysis, a fixed volume of electrolyte was mixed with the MO solution under identical conditions and the UV–Vis spectrum was recorded. The obtained spectrum was compared with those of the pristine MO solution and the ClO^- standards. Photographs of the MO solution before and after electrolysis were also collected to provide visual evidence of any color change associated with hypochlorite formation.

Electrochemical Measurements:

All electrochemical studies were employed in a Metrohm Autolab B. V. (NOVA software) instrument at a temperature of 25 °C. The electrochemical HER and OER were conducted in a typical three-electrode arrangement, where a graphite sheet substrate was used as the working electrode and a graphite rod served as the counter electrode. A Hg/HgO reference electrode was employed for pure alkaline (1.0 M KOH) and alkaline seawater (1:1/1.0 M KOH:natural seawater) environments. A homogeneous uniform slurry was developed with 10 mg of each prepared electrocatalyst in 1 mL of ethanol and 20 μL Nafion binder, sonicated in an ultrasonic sonicator for 30 minutes. The as-prepared 10 μL (mass loading 1.09 mg cm^{-2}) suspension was then drop-casted into a graphite sheet on a surface area 9 mm^2 (3 mm x 3 mm) and dried overnight for further electrochemical analyses. All cathodic and anodic LSV sweeps were measured after 90% *iR*-correction at a scan rate of 10 mV s^{-1}. The electrochemical potentials were converted to the reversible hydrogen electrode (RHE) according to Nernst Equation:

$$E_{RHE} = E_{Hg/HgO} + 0.059 * pH + E^{\circ}_{Hg/HgO} \text{ (for 1.0 M KOH, DSE)} \qquad S1$$

$$(E^{\circ}_{Hg/HgO} = +0.098 \text{ V at 25 °C, pH} = 13.6)$$

The HER overpotential (η) was calculated at different current densities (x) according to the equations (S1):

$$\eta_x = (E_{RHE} - 0 \text{ (vs RHE)}) \times 1000 \text{ in mV} \qquad S2$$

The OER overpotential (η) was estimated using the equations (S1):

$$\eta_x = (E_{RHE} - 1.23 \text{ (vs RHE)}) \times 1000 \text{ in mV} \qquad S3$$

The Tafel slope (*b*) was determined using the Tafel equation as:

$$\eta = b \log j + c \qquad S4$$

where j is the current density, b is the slope, η is the overpotential, and c is a constant.

The OWS performance was conducted using two electrodes in a commercial electrolyzer cell with a Nafion membrane as a separator. The electrochemically active double-layer capacitance was determined via cyclic voltammetry curves performed in a non-faradaic region at different scan rates to evaluate the ECSA in alkaline conditions. The EIS plots were noted to estimate the charge transfer resistance from 100 KHz to 0.01 Hz to an AC voltage of 10 mV amplitude.

For the preparation of standard Pt/C and IrO_2 electrodes, 5 mg of catalyst powder (Pt/C or IrO_2) was dispersed in 500 μL of ethanol containing 10 μL of Nafion solution as a binder to form a homogeneous catalyst ink. The ink was ultrasonicated to ensure uniform dispersion of the catalyst particles. Subsequently, 10 μL of the catalyst ink was drop-cast onto a graphite sheet substrate with a geometric area of 0.09 cm^2 and allowed to dry under ambient conditions. This deposition corresponded to a catalyst mass of 0.098 mg on the electrode surface, resulting in a catalyst loading of 1.09 mg cm^{-2}. Similar 90% *iR*-corrections were done for standard measurements, too.

Instrumentation:

Powder X-ray diffraction (XRD): The XRD pattern of the prepared samples was examined using a Cu Kα radiation (λ = 1.54056 Å) source using a Rigaku Miniflex600 X-Ray diffractometer.

Transmission electron microscopy (TEM): The TEM imaging, the high-angle annular dark field scanning transmission electron microscopy imaging (HAADF-STEM) and the selected area electron diffraction (SAED) pattern were analysed and recorded using a Thermo Scientific Talos S Cryo TEM with 200 kV FEG Transmission. The specimen imaging was inspected on a nickel and copper-coated grid.

Field-emission scanning electron microscope (FESEM): FESEM imaging study performed in a JEOL JSM 6610, 20 kV set with an Energy dispersive X-ray spectrometer was used to investigate the 3D morphology and elemental mapping with EDAX estimations obtained with a JOEL JSM 6610 instrument at 20 kV, with a spot size of 35 and a width of 10 mm.

X-ray photoelectron spectroscopy (XPS): XPS measurements were conducted on a PHI 5000 VersaProbe III with an average depth of analysis of approximately 5 nm. Depth profiling and imaging can be performed over the full range of X-ray beam sizes, including the minimum X-ray beam size of less than 10 μm. All spectra were charge-corrected to adventitious carbon (C 1s = 284.8 eV).

Brunauer-Emmett-Teller (BET): The surface area, pore size, and pore volume estimation were accomplished via Nitrogen adsorption-desorption analyses using a BELSORP-maxII from Microtrac BEL Corp analyser.

Inductively Coupled Plasma Mass Spectrometry (ICP-MS): The elemental concentrations were identified in Agilent ICP-MS 7900 ICP-MS using acid digestion of the nanomaterial. The concentration of prepared nanomaterials evaluated in parts per billion (ppb).

X-ray Absorption Spectroscopy (XAS): Room-temperature XANES and EXAFS measurements at the Cu K-edge, Co K-edge, and Ir L_3-edge were performed on the Scanning EXAFS Beamline (BL-9) at the Indus-2 Synchrotron (2.5 GeV) at RRCAT, Indore, India. This beamline provides an energy resolution of about $\Delta E/E \approx 10^{-4}$ at 10 keV and uses a Si(111) double-crystal monochromator,[1] where one crystal is flat and the second crystal bends to achieve horizontal focusing. Vertical beam focusing was done using Rh/Pt-coated cylindrical mirrors under grazing-incidence conditions, and another mirror was used to collimate the beam. Higher-order harmonic radiation was minimized by detuning the monochromator.

Experimental Procedure for Quasi-operando Raman Measurements

Raman spectroscopy: Raman measurements were conducted using a Renishaw InVia II Raman spectrometer equipped with 532 nm laser source. The system offers a spectral resolution of better than 1 cm^{-1} and employs confocal Raman microscopy with a spot size of <10 µm.

Quasi-operando Raman measurements were carried out to investigate the potential-dependent surface evolution of the catalyst during the OER in alkaline seawater. The catalyst-coated on graphite sheet working electrode was polarized by chronoamperometry at selected potentials (OCP, 1.2, 1.3, 1.4, 1.5, and 1.6 V vs. RHE) for 5 min each in alkaline seawater electrolyte. After each polarization step, the electrode was immediately removed from the electrolyte, gently rinsed with deionized water to remove residual electrolyte, and transferred to the Raman spectrometer for spectral acquisition.

Calculations:

1. Strain (ε_θ) :[2,3]

$$\varepsilon_\theta = (d_\theta - d_o)/d_o \qquad \text{S5}$$

where d_θ is the spacing of the planes under stress, and d_o is the spacing of the planes in the absence of stress.

2. Stress (σ) :[2,3]

$$\sigma = \frac{E}{1+\nu} * (d_\theta - d_o)/d_o \qquad \text{S6}$$

where E is the Young's modulus of Cu (127), and Ir (535), while ν is the Poisson's ratio of Cu (0.33), and Ir (0.26).

3. Lattice mismatch (ε) :[4]

Lattice mismatch (%) = $(a_{std.} - a_o)/a_o$ * 100 S7

where $a_{std.}$ is the standard Ir and Cu metal's lattice constant, while a_o is the prepared composition's lattice constant. (a_{std} for Ir and Cu is 3.8394 and 3.6077 Å, respectively, obtained from the Crystallography Open Database).[5,6]

4. Exchange current density:

Exchange current density (i_{ex}) = RT/nFΘA S8

Where R is the universal gas constant (8.314 J K^{-1} mol^{-1}), T is the reaction temperature (298 K), n is the number of electrons, F is the Faraday constant (96485C mol^{-1}), Θ is the charge transfer resistance calculated from EIS, and A is the area of the nickel foam electrode (0.09 cm^2).[7]

(a) For HER in 1.0 M KOH (calculated from 2-electron process)

$$= \frac{8.314 \text{ J K}^{-1} \text{ mol}^{-1} * 298\text{K}}{2 * 96485 \text{ C mol}^{-1} * 4.86\ \Omega * 0.09 \text{ cm}^2}$$

= 29.35 mA cm^{-2}

(b) For OER in 1.0 M KOH (calculated from 4-electron process)

$$= \frac{8.314 \text{ J K}^{-1} \text{ mol}^{-1} * 298\text{K}}{4 * 96485 \text{ C mol}^{-1} * 2.96\ \Omega * 0.09 \text{ cm}^2}$$

= 24.09 mA cm^{-2}

5. Electrochemical active surface area (ECSA):

The Electrochemical active surface area was calculated from the double-layer capacitance of the modified catalyst electrode in 1.0 M KOH. The slope of curve ΔJ ($J_{anodic} - J_{cathodic}$/ mA cm^{-2}) versus Scan rate (mV s^{-1}) give the value of $2C_{dl}$.[8]

$$ECSA = C_{dl}/C_s \quad \text{S9}$$

Where C_{dl} is the measured double layer capacitance and C_s is the specific capacitance of the catalyst (0.04 mF cm^{-2} considered as an assumed value for a flat surface in 1.0 M KOH)[9,10]

$$\text{ECSA (1.0 M KOH)} = \frac{4.49 \text{ mF}}{0.04 \text{ mF cm}^{-2}}$$

= 112.25 cm^2

Roughness factor of $Co_{0.28}Cu_{0.67}Ir_{0.05}$ can be given as:

RF = ECSA/ area of the loaded catalyst on nickel foam (0.09 cm2)

$$= 112.25\ cm^2/\ 0.09\ cm^2$$

$$= 1247$$

6. Activation energy (E_a):

Activation energy (E_a) was calculated using the Arrhenius equation by plotting log j versus 1/T

$$\partial\ (\log j)/\partial\ (1/T) = -\ E_a/2.303R \quad \text{S10}$$ [11]

Where ∂ (log j)/∂ (1/T) is the slope from Figure 3f.

- In case of 1.0 M KOH

∂ (log j)/∂ (1/T) = − 418.74 K

E_a = 418.74 K x 2.303 x 8.314 $J.mol^{-1}\ K^{-1}$

= 8.017.67 $J.mol^{-1}$

= 8.02 $kJ.mol^{-1}$

7. Turn over frequency (TOF) calculation:[12]

$$\text{Turn over frequency (TOF)} = \frac{j \times A}{n \times F \times m} \quad \text{S11}$$

HER and OER TOF was calculated at current density (j) of η = 58 mV and η = 180 mV in 1.0 M KOH solution respectively, *n* is the number of electrons transferred in the reaction (2 for H_2 and 4 for O_2), *F* is the Faraday's constant (96,485 C mol^{-1}) and *m* is the number of moles of the $Co_{0.28}Cu_{0.67}Ir_{0.05}$ in the electrode.

- TOF (for HER in 1.0 M KOH) = $\dfrac{10\ mA\ cm^{-2} \times 0.09\ cm^2}{2 \times 96{,}485\ C\ mol^{-1} \times 3.92 \times 10^{-7}\ mol}$

$$= 0.011\ s^{-1}$$

- TOF (for OER in 1.0 M KOH) = $\dfrac{10\ mA\ cm^{-2} \times 0.09\ cm^2}{4 \times 96{,}485\ C\ mol^{-1} \times 3.92 \times 10^{-7}\ mol}$

$$= 0.005\ s^{-1}$$

8. Mass activity:[13]

Mass activity (A/g) = j/m S12

Where *j* is the current density (mA cm^{-2}), and *m* is mass loading onto the graphite sheet working electrode (mg cm^{-2}) in 1M KOH conditions.

- Mass Activity $= \frac{10\ \text{mA cm}^2}{1.09\ \text{mg cm}^{-2}}$

$= 9.17\ \text{A g}^{-1}$

9. Faradaic efficiency of $Co_{0.28}Cu_{0.67}Ir_{0.05}$ catalyst for HER and OER in Alkaline Seawater

The theoretical amount of expected total gas volume was evaluated using Faraday's law of electrolysis with the ideal gas law:[14]

$$\text{Vtheo} = \frac{\text{RT}}{\text{F p z}}\text{Q}$$

Here, R is the ideal gas constant, T is the absolute temperature, Q is the charge (at an applied current density of 100 mA cm^{-2} for 120 minutes), F is Faraday's constant, p is the pressure and z is the number of electrons involved.

The faradaic efficiency η_F of the water splitting process was determined as the ratio between the expected total gas volume of the measured and theoretical:

$$\eta\text{F} = \frac{\text{Vmeas}}{\text{Vtheo}}$$

The hydrogen gas volume was measured by the inverted glass burette method

$$\eta\text{F} = \frac{7.9\ \text{mL}}{8.2\ \text{mL}} * 100$$

$$\eta\text{F} = 96.34\ \%$$

The oxygen gas volume was measured by the inverted glass burette method

$$\eta\text{F} = \frac{3.9\ \text{mL}}{4.1\ \text{mL}} * 100$$

$$\eta\text{F} = 95.12\ \%$$

Computational Details

We have taken a model *FCC* unit cell with the composition of Co-Cu-Ir for the sake of our theoretical study, and the geometries were optimized at PBE1PBE/def2-TZVP level.[15] To confirm the nature of the stationary states, harmonic vibrational frequency calculations were

performed at the same level. The absence of imaginary frequencies in the Hessian matrix verified that all structures correspond to local minima. The Gibbs free energies of the intermediates were evaluated using the expression:

$$\Delta G(H^*) = \Delta E(H^*) + \Delta ZPE - T\Delta S \qquad S13$$

where ΔE(H*), ΔZPE, and ΔS represent the binding energy, zero-point energy correction, and entropy change upon adsorption, respectively. All computations were carried out using the Gaussian16 software package.[16] Partial density of states (PDOS) were calculated for all the compositions using Multiwfn program code.[17]

Table S1. Summary of the concentration of elements in the samples via ICP-MS.

S.No.	ICP-MS data compositions	Elemental ratio (μg/g)		
		**Co**	**Cu**	**Ir**
1.	$Co_{0.28}Cu_{0.67}Ir_{0.05}$	165822.7273	392439.3939	38142.4242
2.	$Co_{0.20}Cu_{0.52}Ir_{0.28}$	82889.5377	212529.4404	113383.9416
3.	$Co_{0.29}Cu_{0.71}$	107103.2746	259817.3804	--

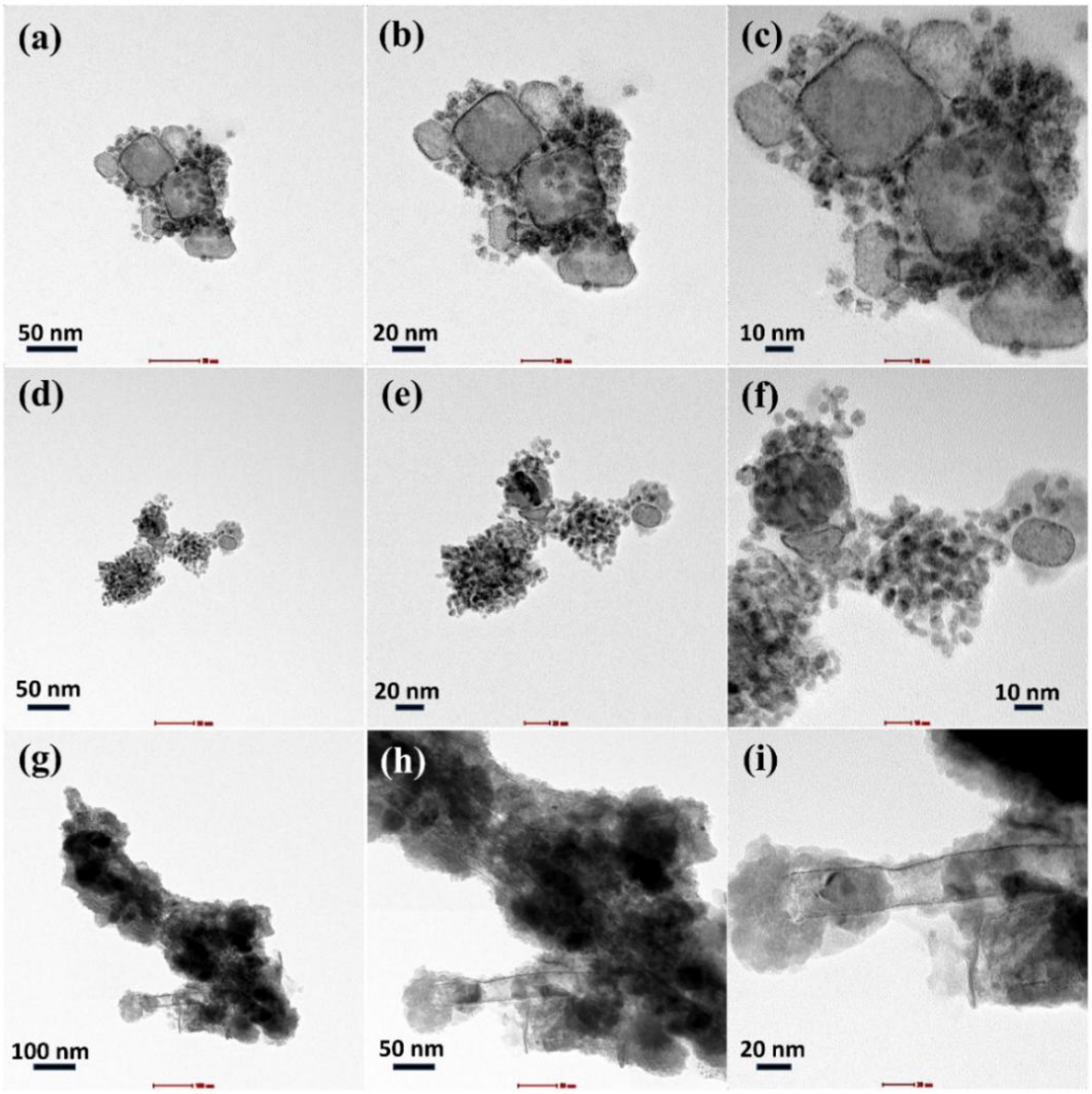

Figure S1. TEM images of controlled study for $Co_{0.28}Cu_{0.67}Ir_{0.05}$ sample (a-c) $Co_{0.28}Cu_{0.67}Ir_{0.05}$-200 °C, (d-f) $Co_{0.28}Cu_{0.67}Ir_{0.05}$-No MA and (g-i) $Co_{0.28}Cu_{0.67}Ir_{0.05}$-No OA.

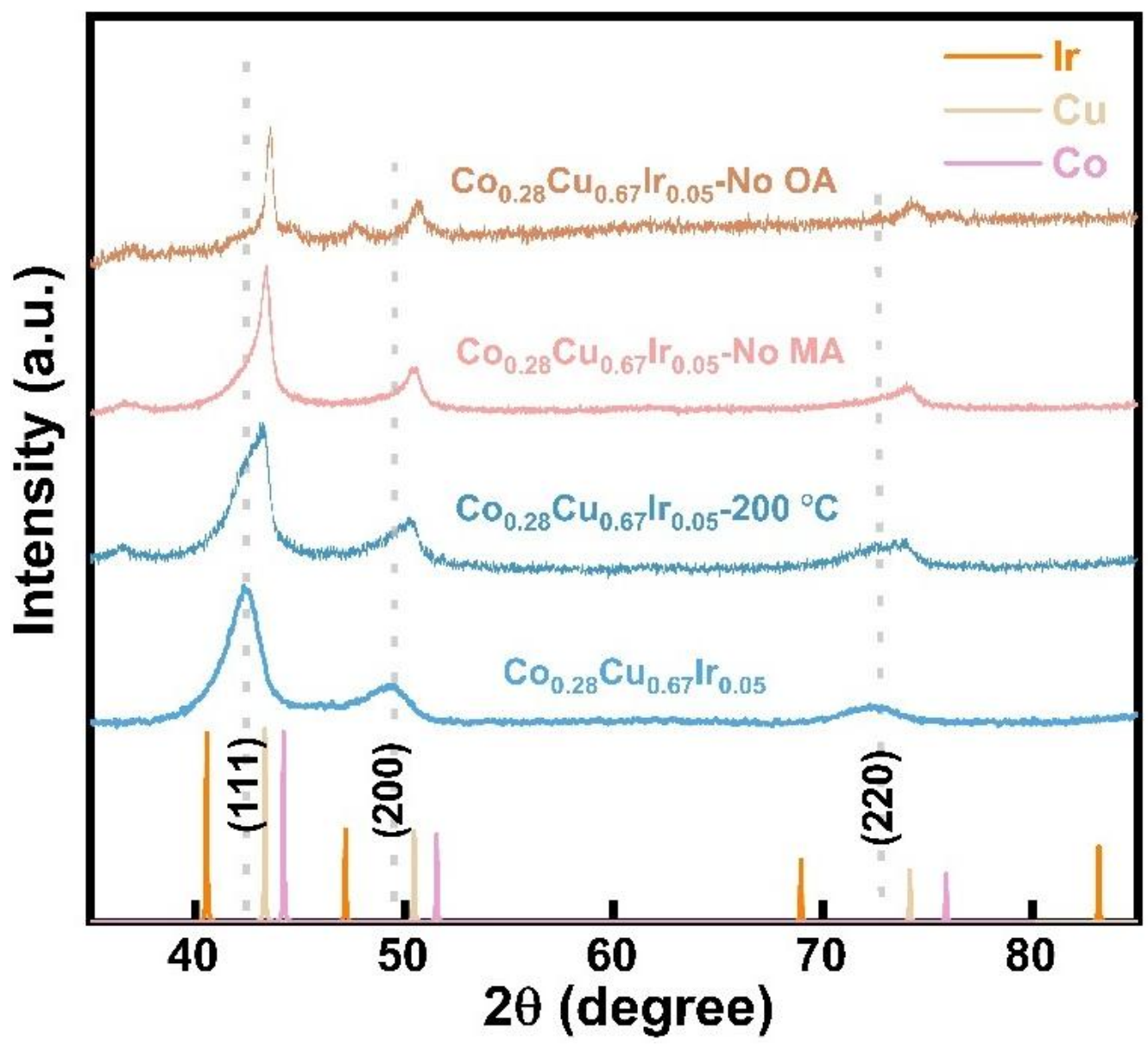

Figure S2. Powder XRD patterns of the controlled study of $Co_{0.28}Cu_{0.67}Ir_{0.05}$ samples.

Table S2. Lattice constant data of $Co_{0.28}Cu_{0.67}Ir_{0.05}$, $Co_{0.20}Cu_{0.52}Ir_{0.28}$, and $Co_{0.29}Cu_{0.71}$ samples.

Samples	Miller indices			Bragg's angle		d-spacing (Å) d_θ	Lattice constant a_o (Å)	Average a (Å)
	h	k	l	2θ	θ			
$Co_{0.28}Cu_{0.67}Ir_{0.05}$	1	1	1	42.45	21.225	2.1277	3.6853	3.6930
	2	0	0	49.32	24.66	1.8462	3.6924	
	2	2	0	72.19	36.095	1.3075	3.6983	
	3	1	1	87.45	43.725	1.1144	3.6962	
$Co_{0.20}Cu_{0.52}Ir_{0.28}$	1	1	1	42.24	21.12	2.1378	3.7028	3.7095
	2	0	0	49.11	24.555	1.8536	3.7072	
	2	2	0	71.87	35.935	1.3126	3.7125	
	3	1	1	86.88	43.44	1.1203	3.7156	
$Co_{0.29}Cu_{0.71}$	1	1	1	43.35	21.675	2.0856	3.6124	3.6157
	2	0	0	50.4	25.2	1.8092	3.6183	
	2	2	0	74.12	37.06	1.2782	3.6153	
	3	1	1	89.88	44.94	1.0905	3.6168	

Table S3. Lattice mismatch calculation data of the prepared compositions to the standard Ir and Cu.

Samples	Lattice constant $a_{std.}$ (Å)	Lattice constant a_o (Å)	Lattice mismatch (%)
$Co_{0.28}Cu_{0.67}Ir_{0.05}$ w.r.t. Std. Cu	3.6077	3.6930	-2.31
$Co_{0.20}Cu_{0.52}Ir_{0.28}$ w.r.t. Std. Cu	3.6077	3.7095	-2.74
$Co_{0.29}Cu_{0.71}$ w.r.t. Std. Cu	3.6077	3.6157	-0.221

Table S4. *d*-spacing calculations of standard Ir for lattice strain and stress calculations.

Samples	Bragg's Angle		d-spacing (Å) d_o
	**2θ**	**θ**	
Std. Ir	40.55	20.276	2.2228
	47.18	23.588	1.9250
	68.93	34.465	1.3612
	83.15	41.574	1.1608

Table S5. *d*-spacing calculations of standard Cu for lattice strain and stress calculations.

**Samples**	**Bragg's Angle**		**d-spacing (Å) d_o**
	**2θ**	**θ**	
Std. Cu	43.32	21.659	2.0871
	50.45	25.225	1.8075
	74.13	37.063	1.2781
	89.94	44.969	1.0900

Table S6. Lattice strain and stress data of $Co_{0.28}Cu_{0.67}Ir_{0.05}$, $Co_{0.20}Cu_{0.52}Ir_{0.28}$, and $Co_{0.29}Cu_{0.71}$ samples concerning the standard Cu crystal structure (and lattice parameter).

**Samples**	**Bragg's Angle**		**d-spacing (Å) d_θ**	**Strain ε_θ**	**Average Strain**	**Stress σ**	**Average Stress (N/m²)**
	2θ	θ					
$Co_{0.28}Cu_{0.67}Ir_{0.05}$	42.45	21.225	2.1277	0.0195	0.0220	1.8588	2.1047
	49.32	24.66	1.8462	0.0214		2.0453	
	72.19	36.095	1.3075	0.0230		2.2001	
	87.26	43.63	1.1164	0.0242		2.3145	
$Co_{0.20}Cu_{0.52}Ir_{0.28}$	42.24	21.12	2.1378	0.0243	0.0261	2.3204	2.4974
	49.11	24.555	1.8536	0.0255		2.4364	
	71.87	35.935	1.3126	0.0270		2.5761	
	86.88	43.44	1.1203	0.0278		2.6565	
$Co_{0.29}Cu_{0.71}$	43.35	21.67	2.0856	-0.0007	0.00002	-0.0679	0.0189
	50.4	25.2	1.8092	0.0009		0.0875	
	74.12	37.06	1.2782	0.00008		0.0077	
	89.88	44.94	1.0905	0.0005		0.0483	

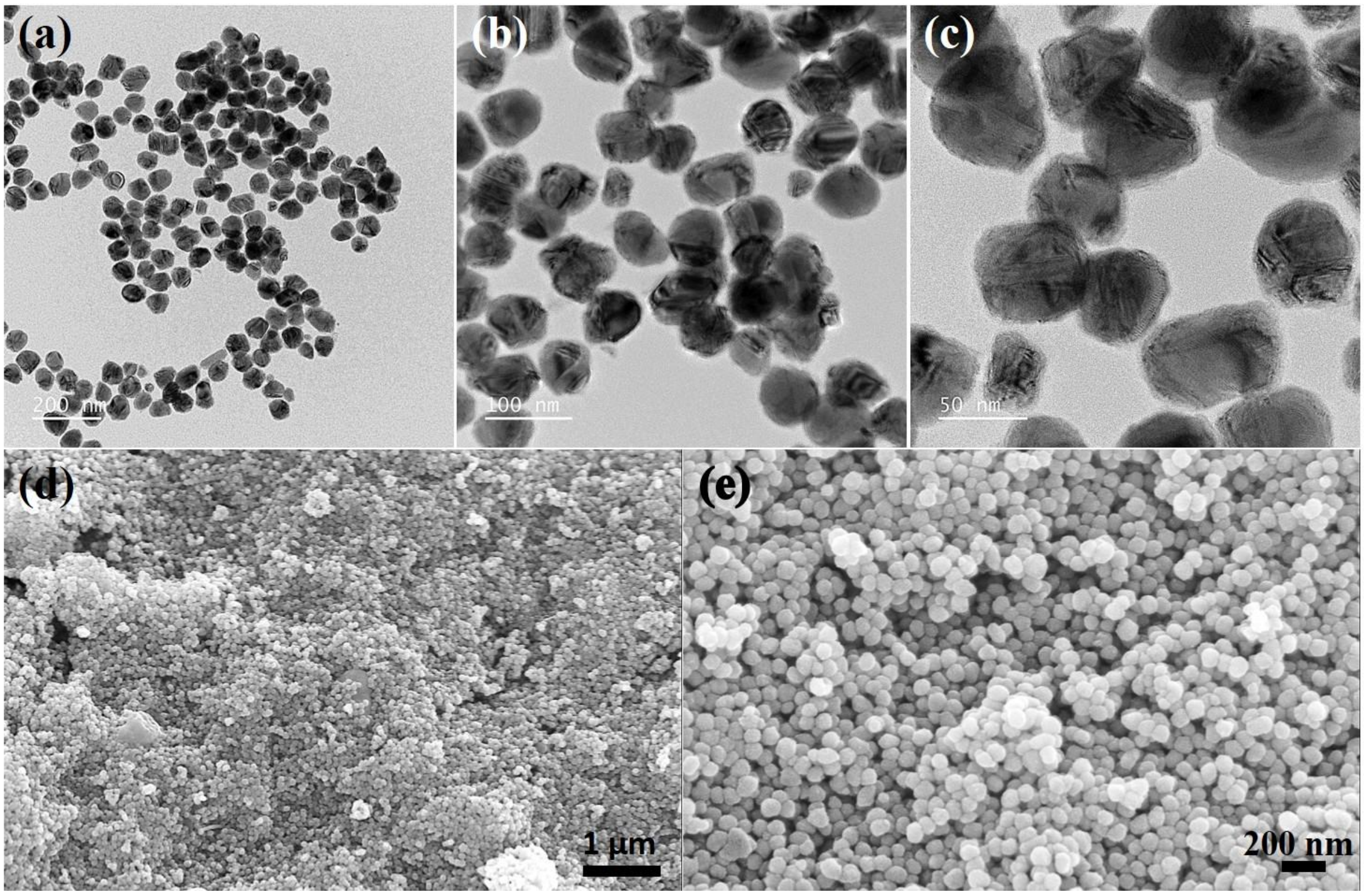

Figure S3. (a-c) TEM and (d, e) FESEM images of $Co_{0.29}Cu_{0.71}$ alloy NPs.

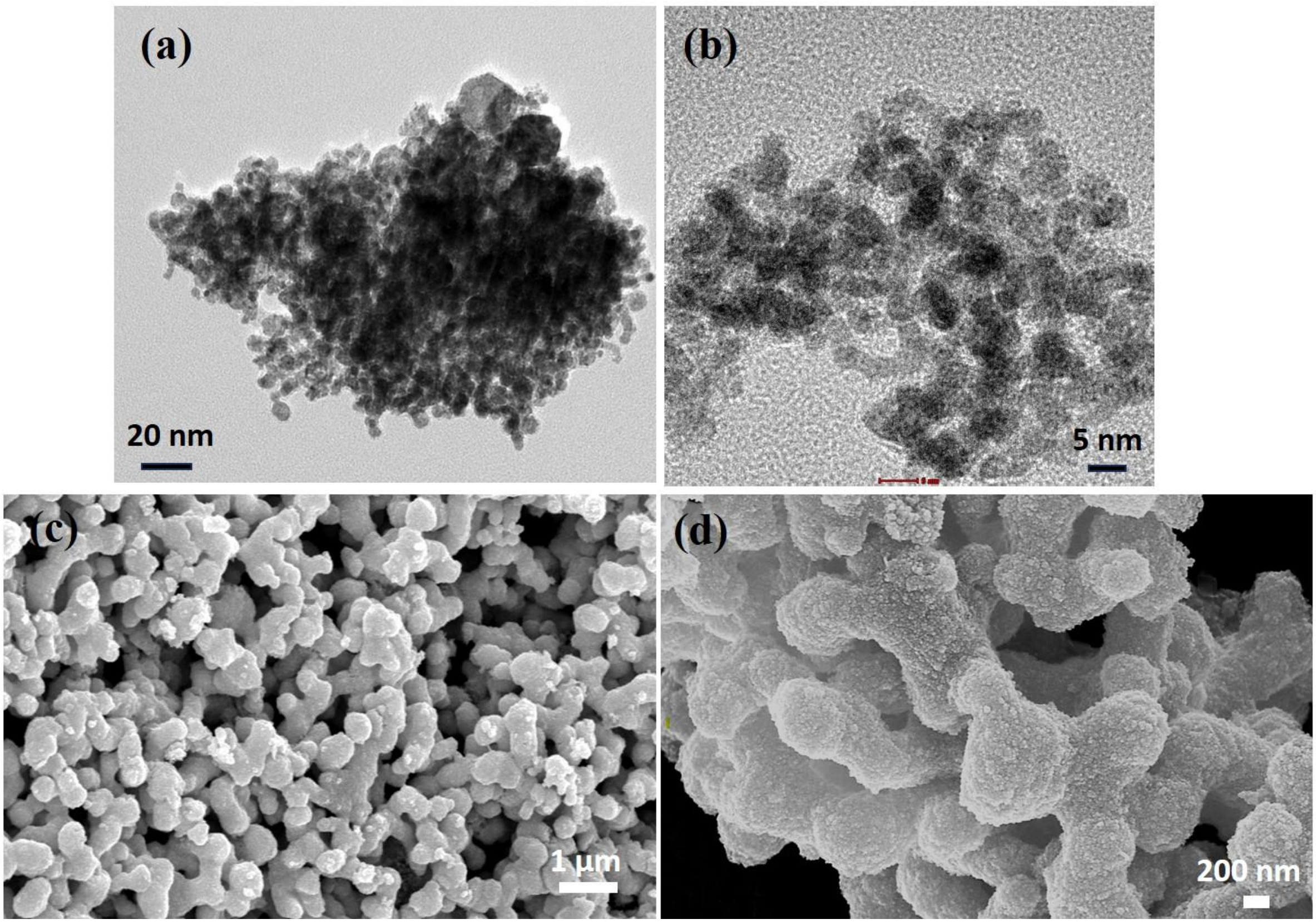

Figure S4. (a, b) TEM and (c, d) FESEM images of $Co_{0.28}Cu_{0.67}Ir_{0.05}$ alloy NPs.

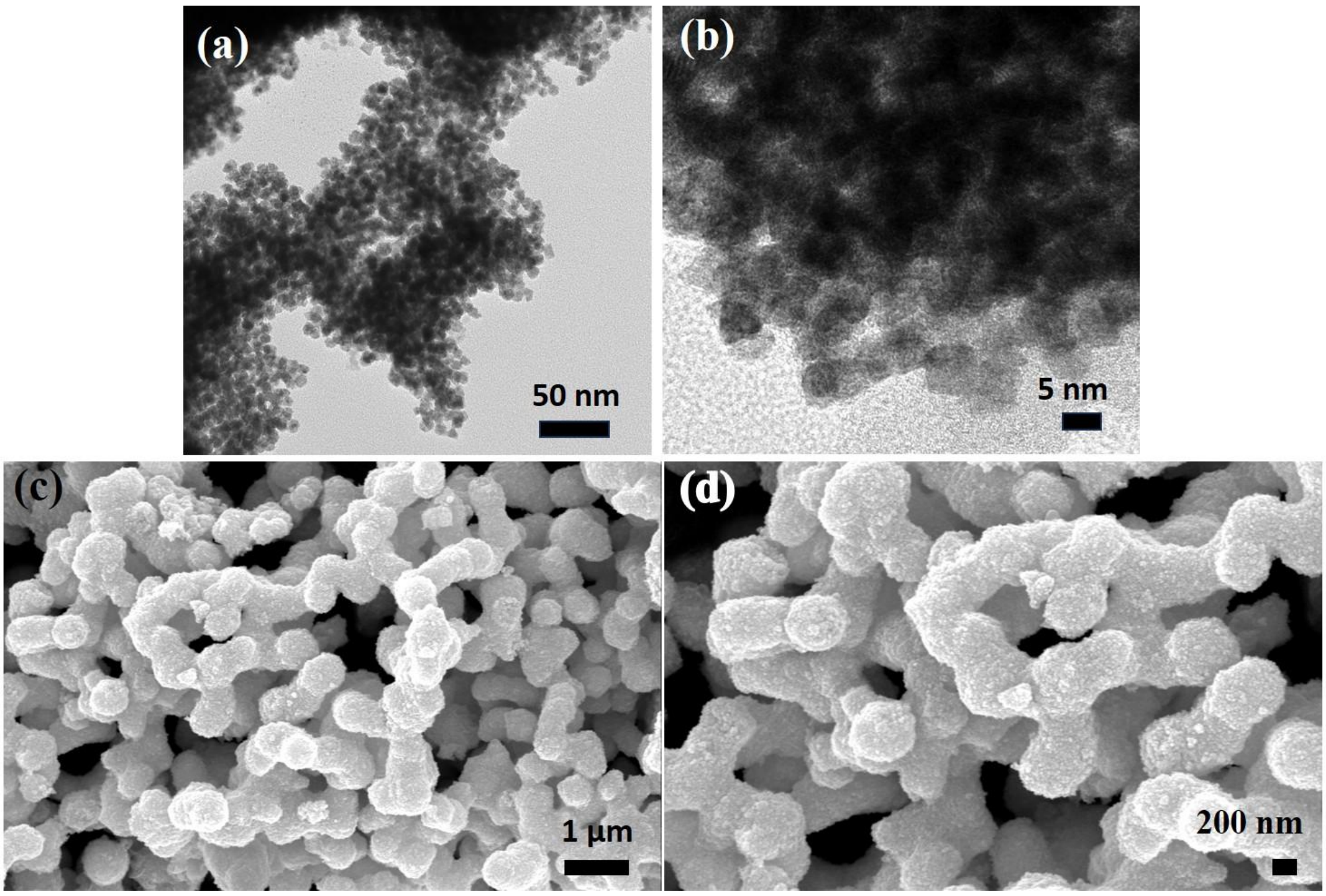

Figure S5. (a, b) TEM and (c, d) FESEM images of $Co_{0.20}Cu_{0.52}Ir_{0.28}$ NPs.

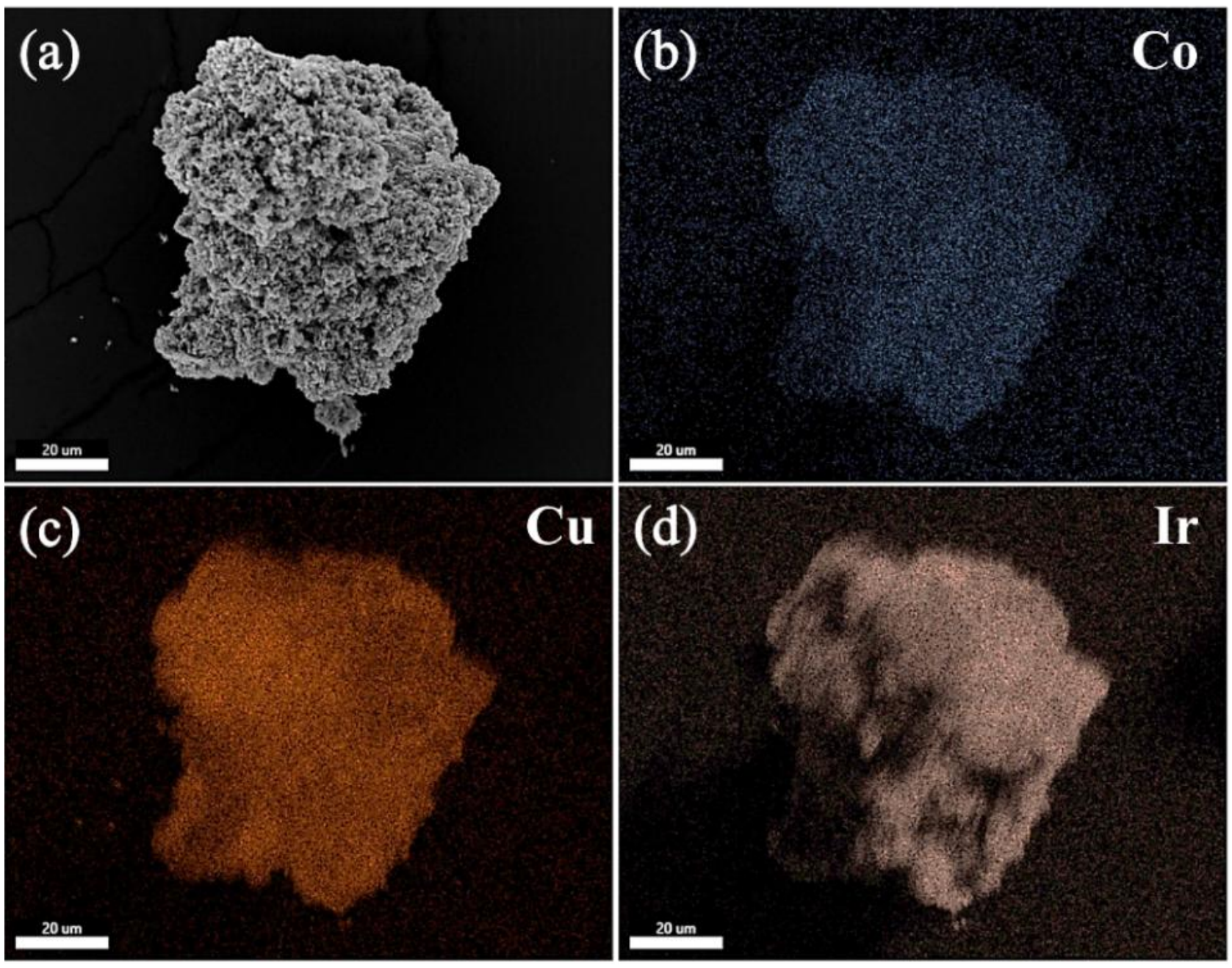

Figure S6. FESEM EDS elemental mapping of $Co_{0.28}Cu_{0.67}Ir_{0.05}$ sample, where (a) FESEM image of the selected region with corresponding mapping of (b) Co, (c) Cu, and (d) Ir metals.

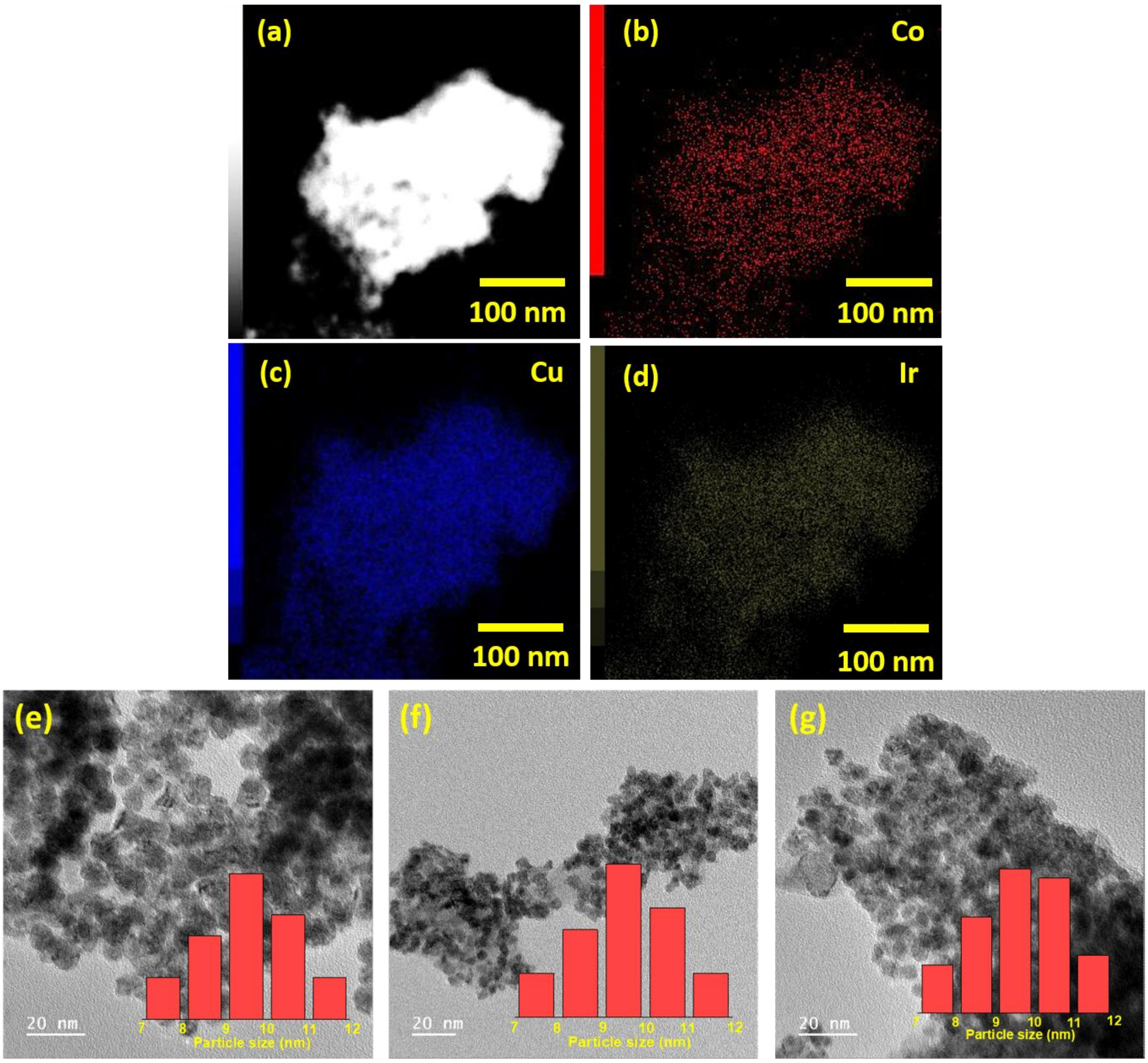

Figure S7. (a-d) HAADF-STEM image and elemental mapping of $Co_{0.28}Cu_{0.67}Ir_{0.05}$ confirming the uniform distribution of Co, Cu and Ir. (e-f) TEM images from three different regions with corresponding particle size distribution histograms reveal homogeneous morphology and consistent particle size throughout the catalyst.

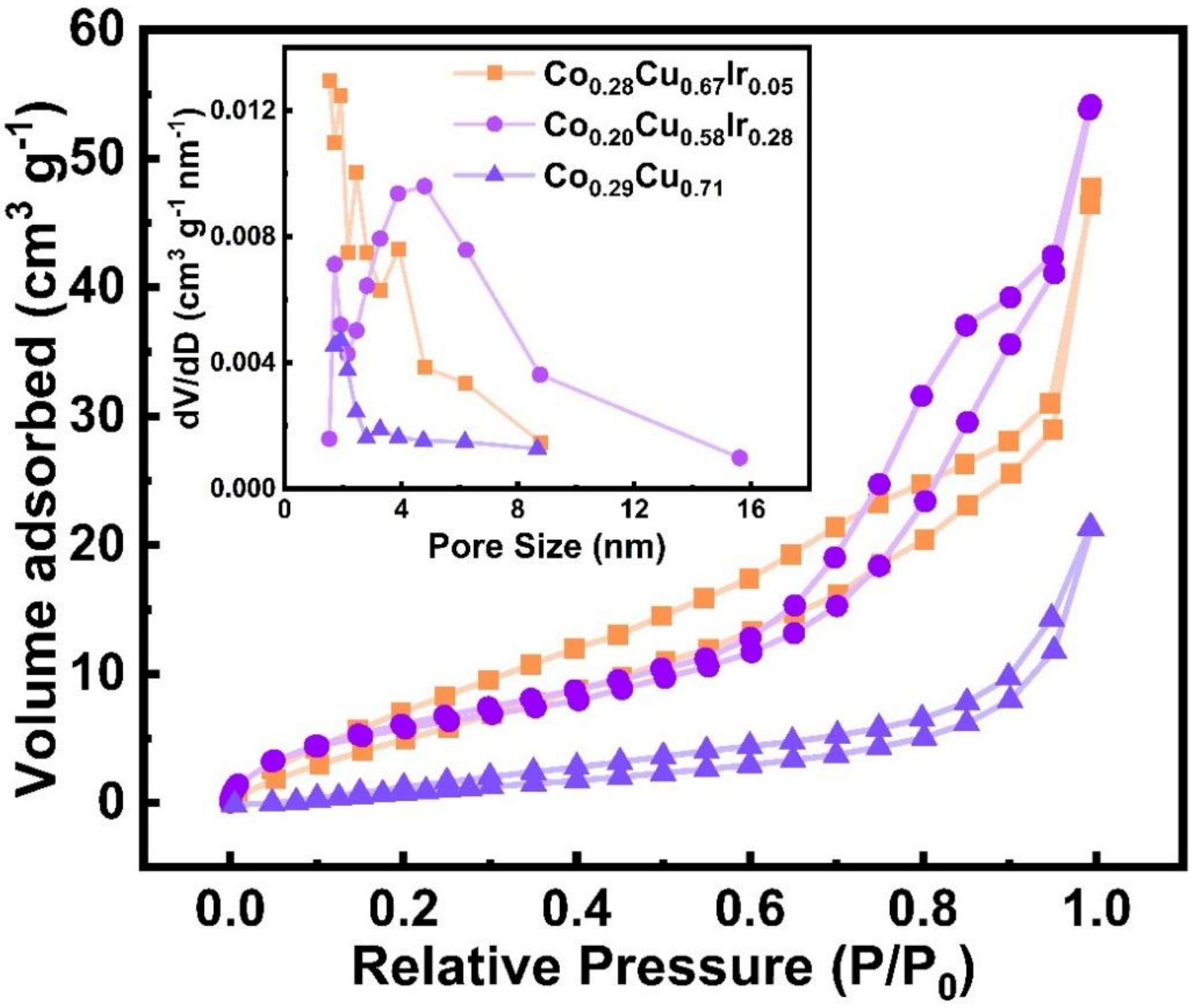

Figure S8. BET N_2 adsorption-desorption isotherms of $Co_{0.28}Cu_{0.67}Ir_{0.05}$, $Co_{0.20}Cu_{0.52}Ir_{0.28}$ and $Co_{0.29}Cu_{0.71}$ NPs of all compositions (Inset: BJH pore size distribution).

Table S7. BET surface area, pore radius, and pore volume distribution data for the studied catalysts.

S.No.	Catalysts	BET surface area (m^2 g^{-1})	Pore Radius (nm)	Pore Volume (cm^3 g^{-1})
1.	$Co_{0.28}Cu_{0.67}Ir_{0.05}$	23.54	1.9	0.037
2.	$Co_{0.20}Cu_{0.52}Ir_{0.28}$	21.76	3.8	0.069
3.	$Co_{0.29}Cu_{0.71}$	7.807	1.916	0.015

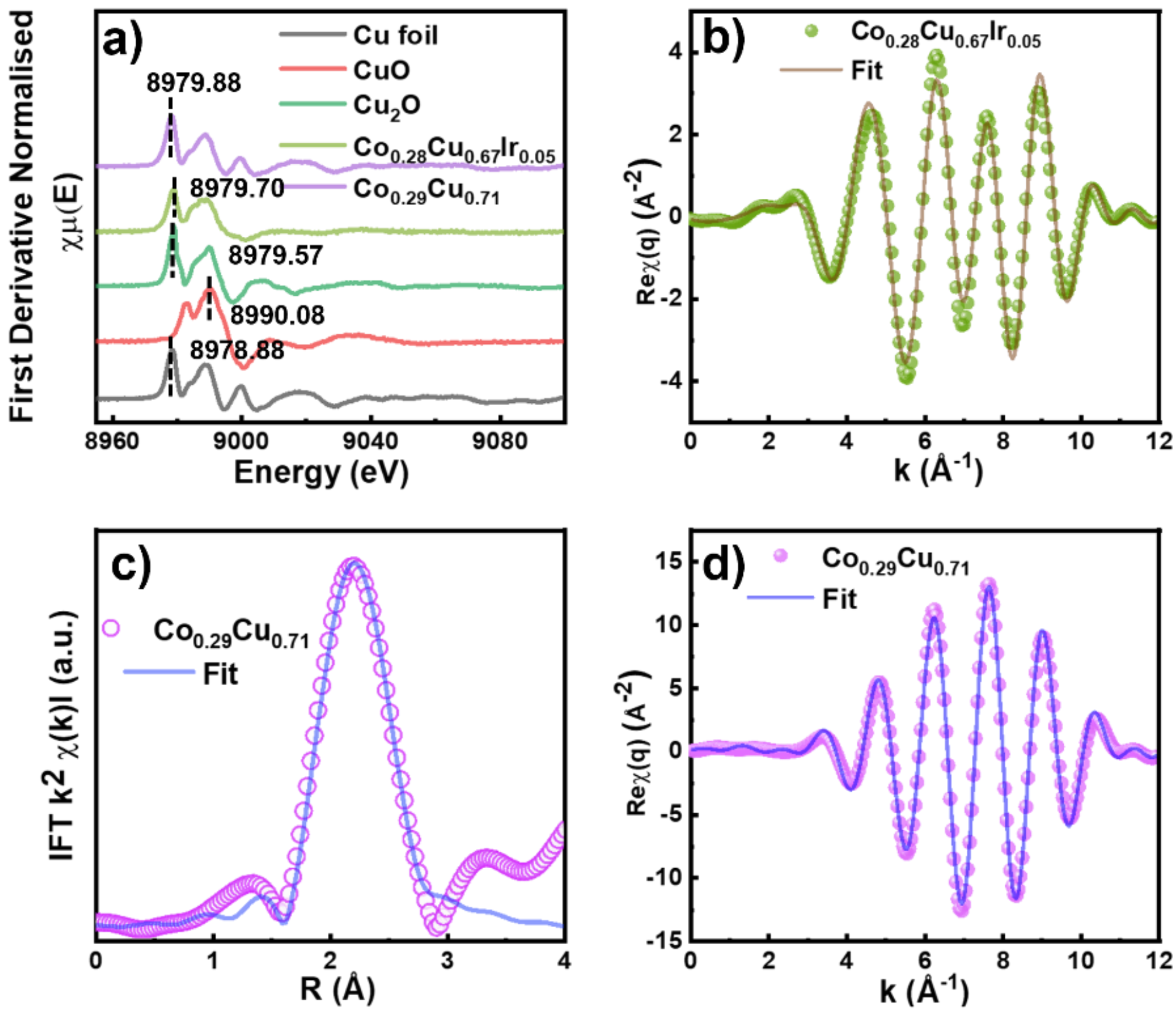

Figure S9. (a) First derivatives of the Cu K-edge, (b) fitting curves of the EXAFS spectra of $Co_{0.28}Cu_{0.67}Ir_{0.05}$ in k space, (c) FT k^2-weighted Cu K-edge EXAFS spectra of $Co_{0.29}Cu_{0.71}$ and (d) fitting curves of the EXAFS spectra of $Co_{0.29}Cu_{0.71}$ in k space.

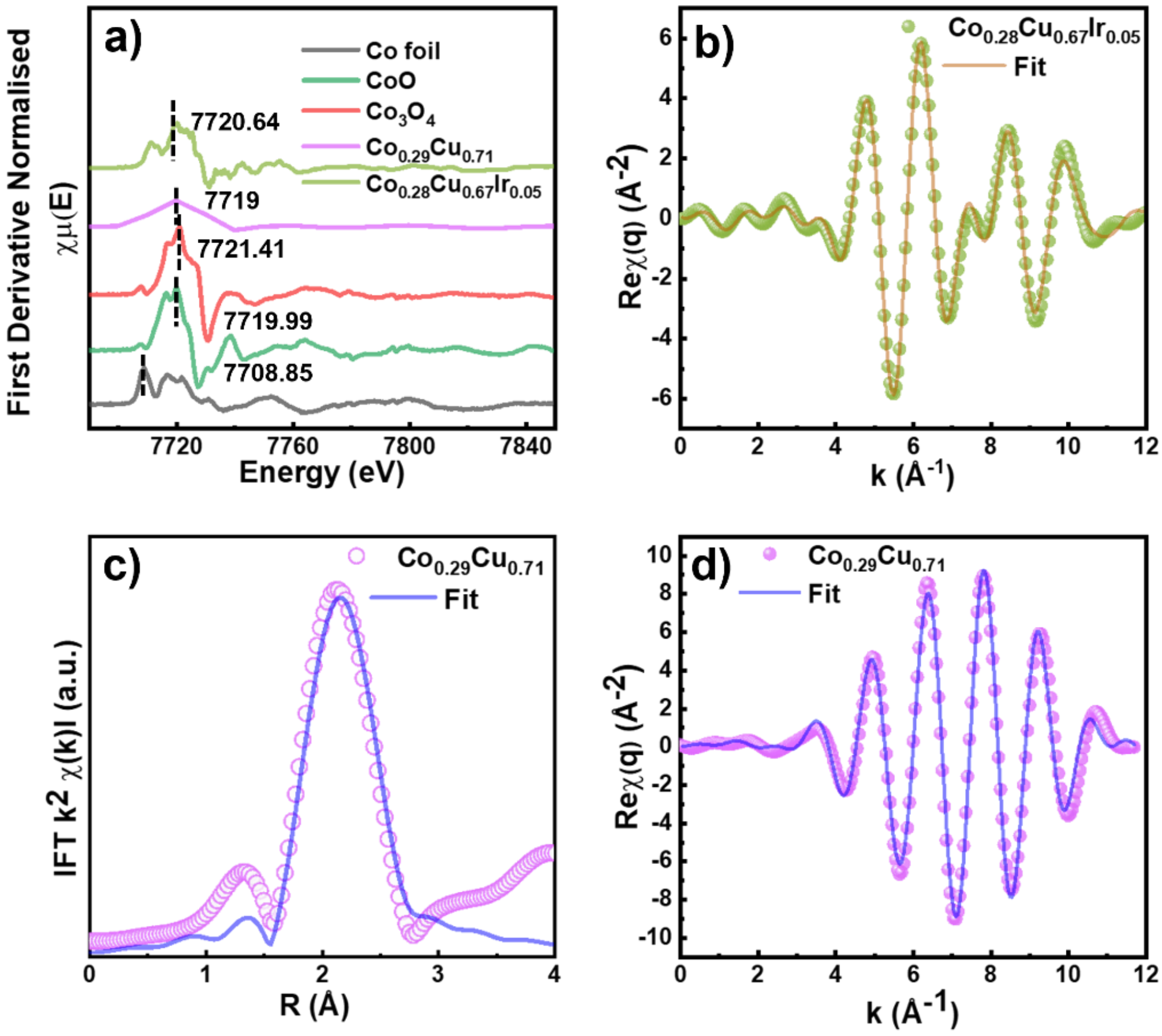

Figure S10. (a) First derivatives of the Co K-edge, (b) fitting curves of the EXAFS spectra of $Co_{0.28}Cu_{0.67}Ir_{0.05}$ in k space, (c) FT k^2-weighted Co K-edge EXAFS spectra of $Co_{0.29}Cu_{0.71}$ and (d) fitting curves of the EXAFS spectra of $Co_{0.29}Cu_{0.71}$ in k space.

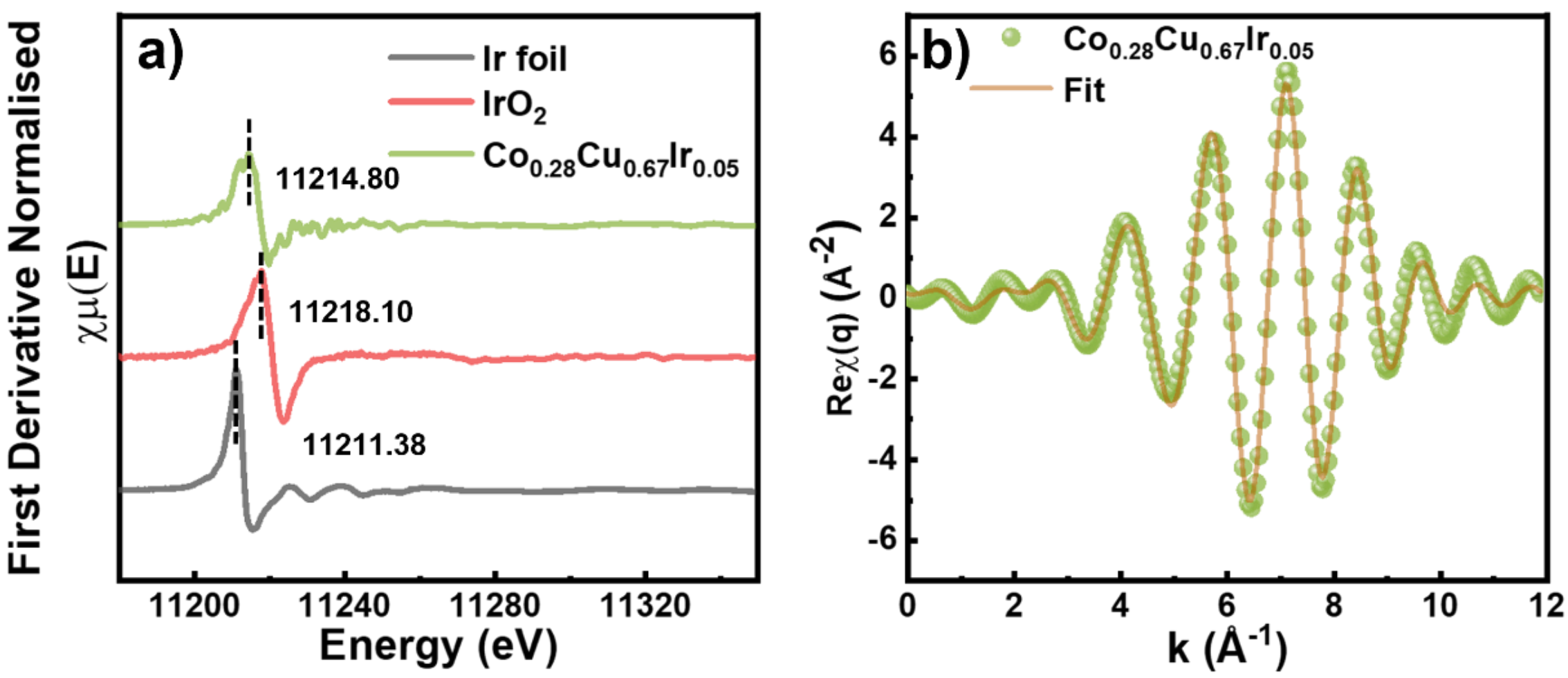

Figure S11. (a) First derivatives of the Ir L_3-edge, (b) fitting curves of the EXAFS spectra of $Co_{0.28}Cu_{0.67}Ir_{0.05}$ in k space.

Table S8. Structural parameters obtained from the Cu K-edge, Co K-edge and Ir L_3-edge EXAFS fitting. The coordination number (CN), R is the bond distance, σ2 is the Debye–Waller factor, and R is the R-factor (goodness of fit).

**Cu K-edge fitting results**					
**Samples**	**Path**	**CN**	**R(Å)**	**σ^2**	**R-factor**
$Co_{0.29}Cu_{0.71}$	1 Cu-Co	5.56 ± 0.02	2.55 ± 0.01	0.005	0.006
$Co_{0.28}Cu_{0.67}Ir_{0.05}$	1 Cu-Co	3.6 ± 0.02	1.89 ± 0.02	0.026	0.030
	1 Cu-Ir	2 ± 0.10	2.52 ± 0.01	0.006	
**Co K-edge fitting results**					
**Samples**	**Path**	**CN**	**R(Å)**	**σ^2**	**R-factor**
$Co_{0.29}Cu_{0.71}$	1 Co-Cu	4.04 ± 0.01	2.50 ± 0.01	0.006	0.019
$Co_{0.28}Cu_{0.67}Ir_{0.05}$	1 Co-Cu	2.12 ± 0.01	2.12 ± 0.02	0.011	0.028
	2 Co-Ir	1.26 ± 0.01	2.62 ± 0.01	0.001	
**Ir L_3-edge fitting results**					
**Samples**	**Path**	**CN**	**R(Å)**	**σ^2**	**R-factor**
$Co_{0.28}Cu_{0.67}Ir_{0.05}$	1 Ir-Cu	1.11 ± 0.07	2.11 ± 0.01	0.006	0.018
	1 Ir-Co	2.22 ± 0.02	2.56 ± 0.02	0.001	

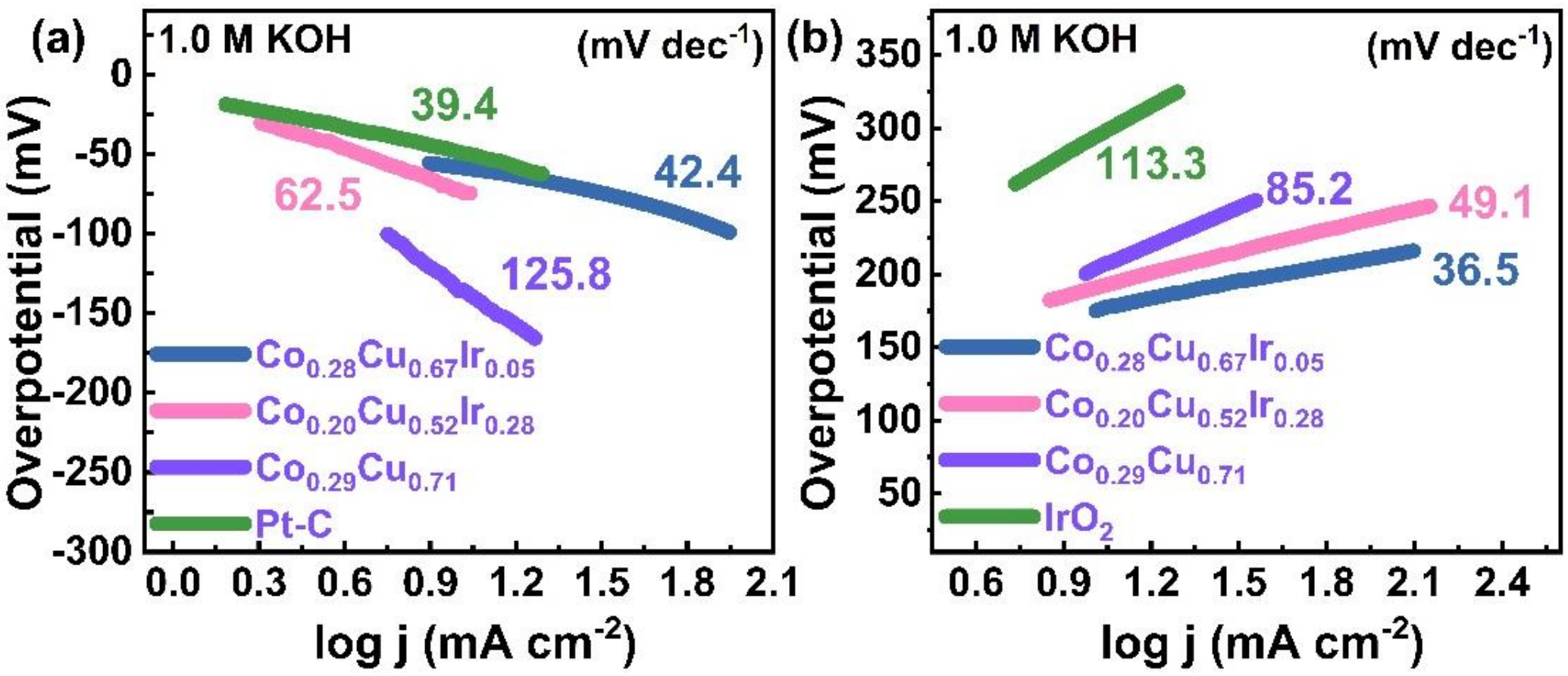

Figure S12. Tafel slope (a) HER and (b) OER of all prepared catalysts with commercial Pt/C (HER) and IrO_2 (OER) in 1.0 M KOH, respectively.

Table S9. Overpotential (mV) and Tafel slope (mV dec^{-1}) comparison of studied electrocatalysts in 1.0 M KOH.

S. No.	Catalysts	HER		OER	
		**Overpotential (mV) at 10 mA cm^{-2}**	**Tafel slope**	**Overpotential (mV) at 10 mA cm^{-2}**	**Tafel slope**
1.	$Co_{0.28}Cu_{0.67}Ir_{0.05}$	58	42.4	180	36.5
3.	$Co_{0.20}Cu_{0.52}Ir_{0.28}$	72	62.5	202	49.1
4.	$Co_{0.29}Cu_{0.71}$	135	125.8	221	85.2
6.	Standard	39 (Pt/C)	39.4	298 (IrO_2)	113.3

Table S10. Overpotential (mV) data of $Co_{0.28}Cu_{0.67}Ir_{0.05}$ catalysts at various high current densities in different electrolytic conditions.

Electrolytes		$Co_{0.28}Cu_{0.67}Ir_{0.05}$		
		**Overpotential at 10 mA cm^{-2}**	**Overpotential at 100 mA cm^{-2}**	**Overpotential at 1000 mA cm^{-2}**
1.0 M KOH	HER	58	103	302
	OER	180	257	370
Alkaline Seawater	HER	62	148	437
	OER	203	290	393

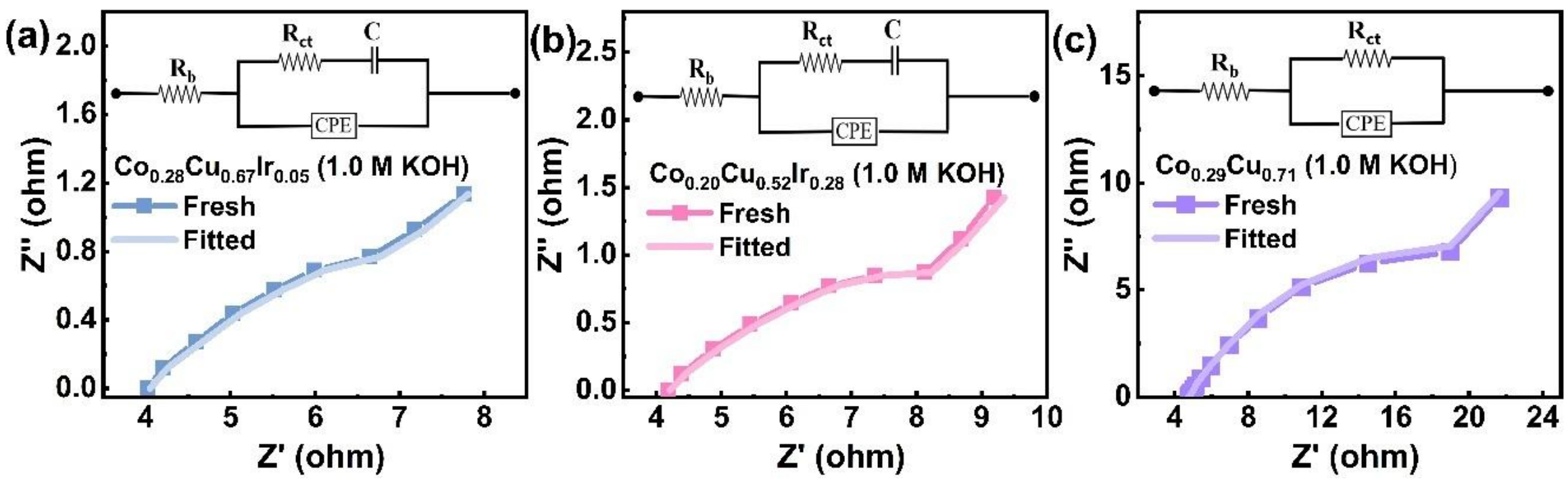

Figure S13. Nyquist plot along with fitted curve and circuit diagram for (a) $Co_{0.28}Cu_{0.67}Ir_{0.05}$, (b) $Co_{0.20}Cu_{0.52}Ir_{0.28}$, and (c) $Co_{0.29}Cu_{0.71}$ samples for HER in 1.0 M KOH.

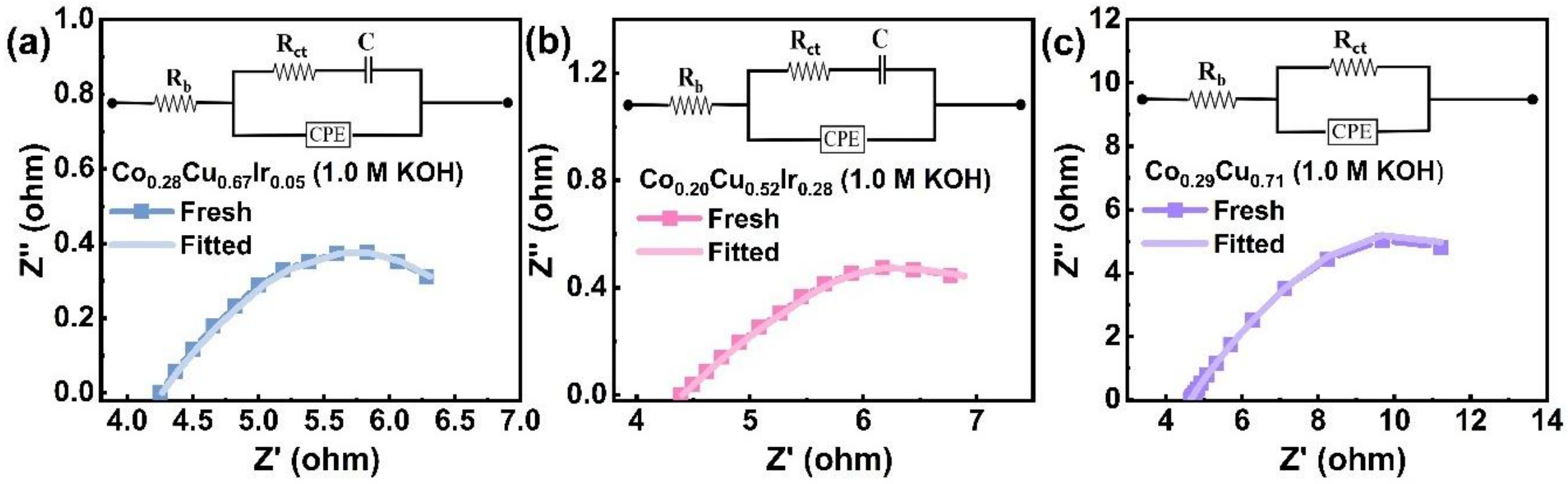

Figure S14. Nyquist plot along with fitted curve and circuit diagram for (a) $Co_{0.28}Cu_{0.67}Ir_{0.05}$, (b) $Co_{0.20}Cu_{0.52}Ir_{0.28}$, and (c) $Co_{0.29}Cu_{0.71}$ samples for OER in 1.0 M KOH.

Table S11. Charge-transfer resistance and Exchange current density results of the examined catalysts in 1.0 M KOH medium.

S. No.	Catalysts (code)	HER (-0.14 V vs RHE)		OER (1.48 V vs RHE)	
		Charge transfer resistance (R_{ct}, ohm)	Exchange current density (i_{ex}/mA cm^{-2})	Charge transfer resistance (R_{ct}, ohm)	Exchange current density (i_{ex}/mA cm^{-2})
1.	$Co_{0.28}Cu_{0.67}Ir_{0.05}$	4.86	29.35	2.96	24.09
2.	$Co_{0.20}Cu_{0.52}Ir_{0.28}$	7.32	19.49	3.55	20.09
3.	$Co_{0.29}Cu_{0.71}$	24.21	5.89	9.54	7.48

Table S12. EIS equivalent circuit fitting parameters of the tested catalysts in 1.0 M KOH electrolyte.

S. No.	Catalysts	HER				OER			
		R_b (Ω)	R_{ct} (Ω)	C (μF)	CPE	R_b (Ω)	R_{ct} (Ω)	C (mF)	CPE
1.	$Co_{0.28}Cu_{0.67}Ir_{0.05}$	4.06	4.86	6.92	56.1 mMho*s^0.14	4.49	2.96	6.53	7.34 mMho*s^0.471
2.	$Co_{0.20}Cu_{0.52}Ir_{0.28}$	4.5	7.32	22	45.2 mMho*s^0.066	4.38	3.55	7.49	66.1 mMho*s^0.191
3.	$Co_{0.29}Cu_{0.71}$	4.75	24.21	-	17.1 mMho*s^0.458	4.66	9.54	-	CPE 49.4 mMho*s^0.654

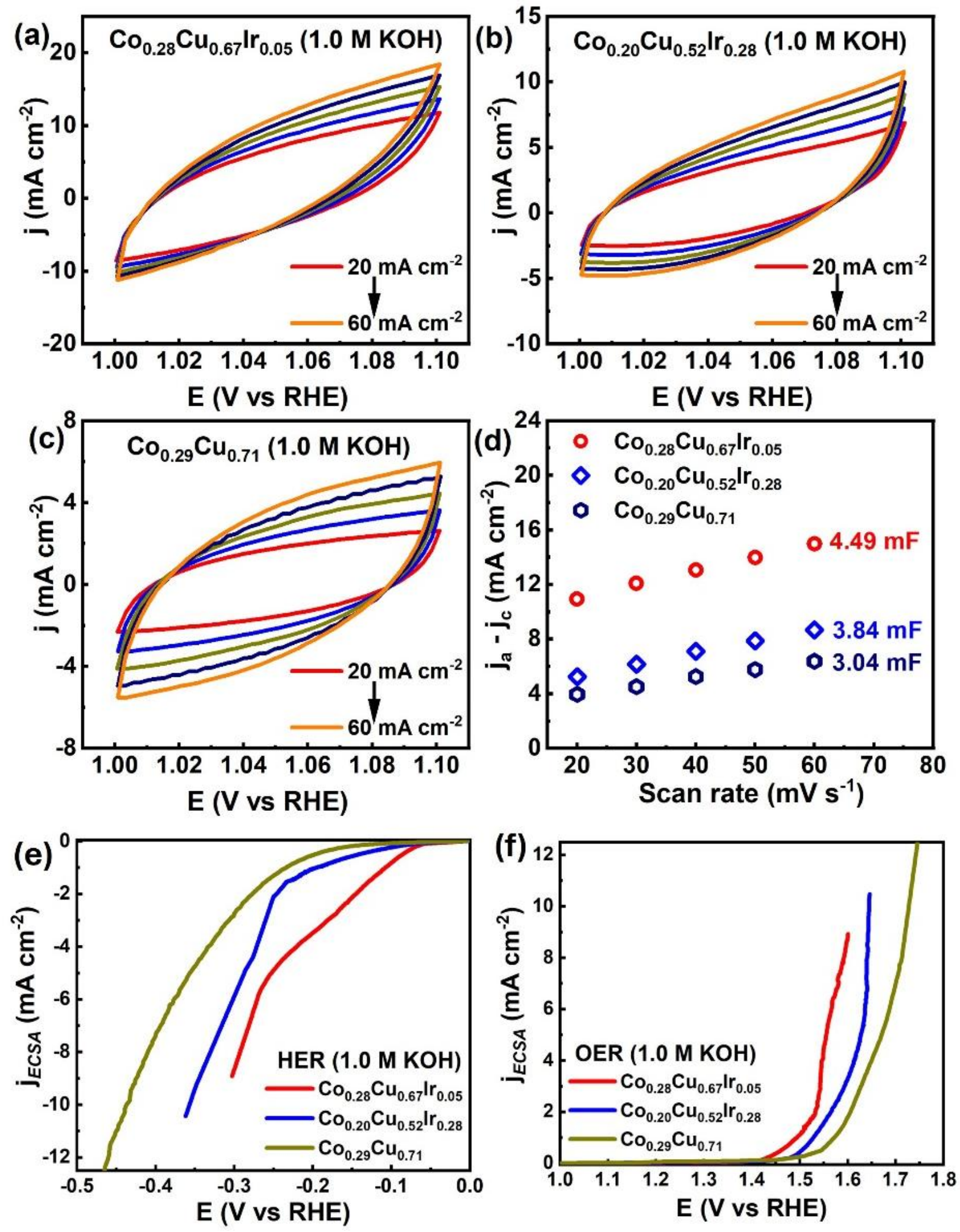

Figure S15. CV curve obtained in a non-faradaic region at various scan rates (a) $Co_{0.28}Cu_{0.67}Ir_{0.05}$, (b) $Co_{0.20}Cu_{0.52}Ir_{0.28}$, and (c) $Co_{0.29}Cu_{0.71}$ and estimation of the (d) C_{dl} (Δj vs. scan rates) in alkaline condition. (e,f) ECSA-normalized LSV curves for all samples

Table S13. ECSA results of the studied catalysts under alkaline environment.

**S. No.**	**Catalysts**	**1.0 M KOH**		
		**Double layer Capacitance (C_{dl}, mF)**	**ECSA (cm^2)**	**Roughness Factor**
1.	$Co_{0.28}Cu_{0.67}Ir_{0.05}$	4.49	112.25	1247
2.	$Co_{0.20}Cu_{0.52}Ir_{0.28}$	3.84	96.0	1066
3.	$Co_{0.29}Cu_{0.71}$	3.04	76.0	844

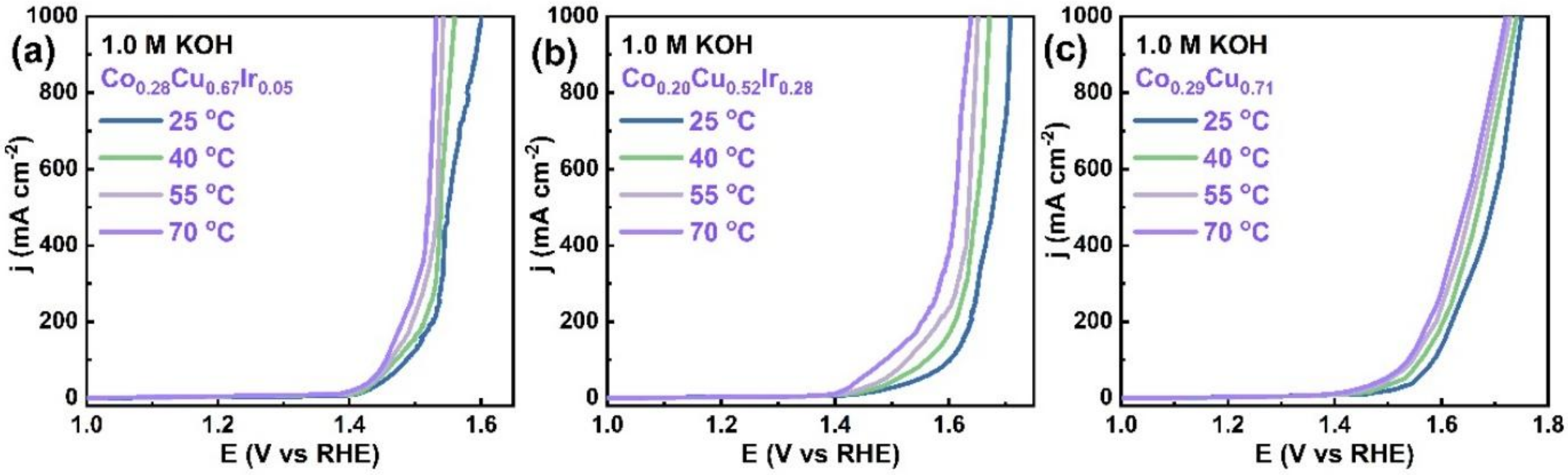

Figure S16. LSV curves of (a) $Co_{0.28}Cu_{0.67}Ir_{0.05}$, (b) $Co_{0.20}Cu_{0.52}Ir_{0.28}$, and (c) $Co_{0.29}Cu_{0.71}$ samples at various temperatures in 1.0 M KOH.

Table S14. Activation energy comparison of the examined catalysts in alkaline environment.

**S. No.**	**Catalysts**	**Activation Energy (E_a/kJ mol^{-1})**
		**1.0 M KOH**
1.	$Co_{0.28}Cu_{0.67}Ir_{0.05}$	8.02
2.	$Co_{0.20}Cu_{0.52}Ir_{0.28}$	16.37
3.	$Co_{0.29}Cu_{0.71}$	17.71

Table S15. Comparison of TOF and mass activity of $Co_{0.28}Cu_{0.67}Ir_{0.05}$ catalysts in different electrolytic conditions.

**Electrolytes**	**Turnover Frequency (TOF/ s^{-1})**		**Mass Activity (A g^{-1})**	
	**HER (η = 23 mV)**	**OER (η = 180 mV)**	**HER (η = 23 mV)**	**OER (η = 180 mV)**
1.0 M KOH	0.0038	0.0059	2.91	9.17
Alkaline Seawater	0.0026	0.0046	1.99	7.08

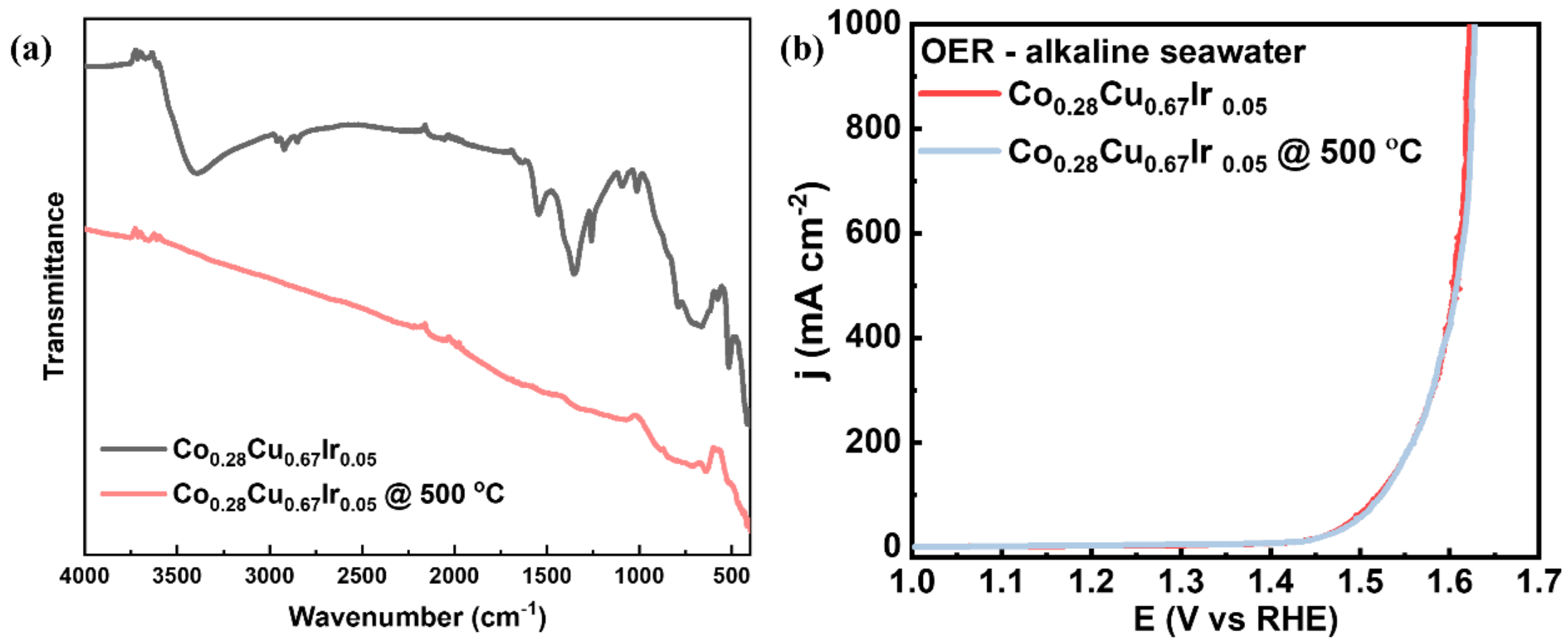

Figure S17. (a) FTIR spectra of $Co_{0.28}Cu_{0.67}Ir_{0.05}$ and $Co_{0.28}Cu_{0.67}Ir_{0.05}$@500 °C catalysts. (b) Comparison of the OER polarization curves of $Co_{0.28}Cu_{0.67}Ir_{0.05}$ and $Co_{0.28}Cu_{0.67}Ir_{0.05}$@500 °C.

Table S16. Summary of the ICP-MS for additional Co–Cu–Ir alloy compositions with low and high optimised Ir content.

S.No.	ICP-MS data compositions	Elemental ratio (μg/g)		
		Co	Cu	Ir
1.	$Co_{0.22}Cu_{0.70}Ir_{0.08}$	156259.1230	509713.1105	56142.9306
2.	$Co_{0.14}Cu_{0.49}Ir_{0.37}$	29531.9728	99888.4354	77447.6190

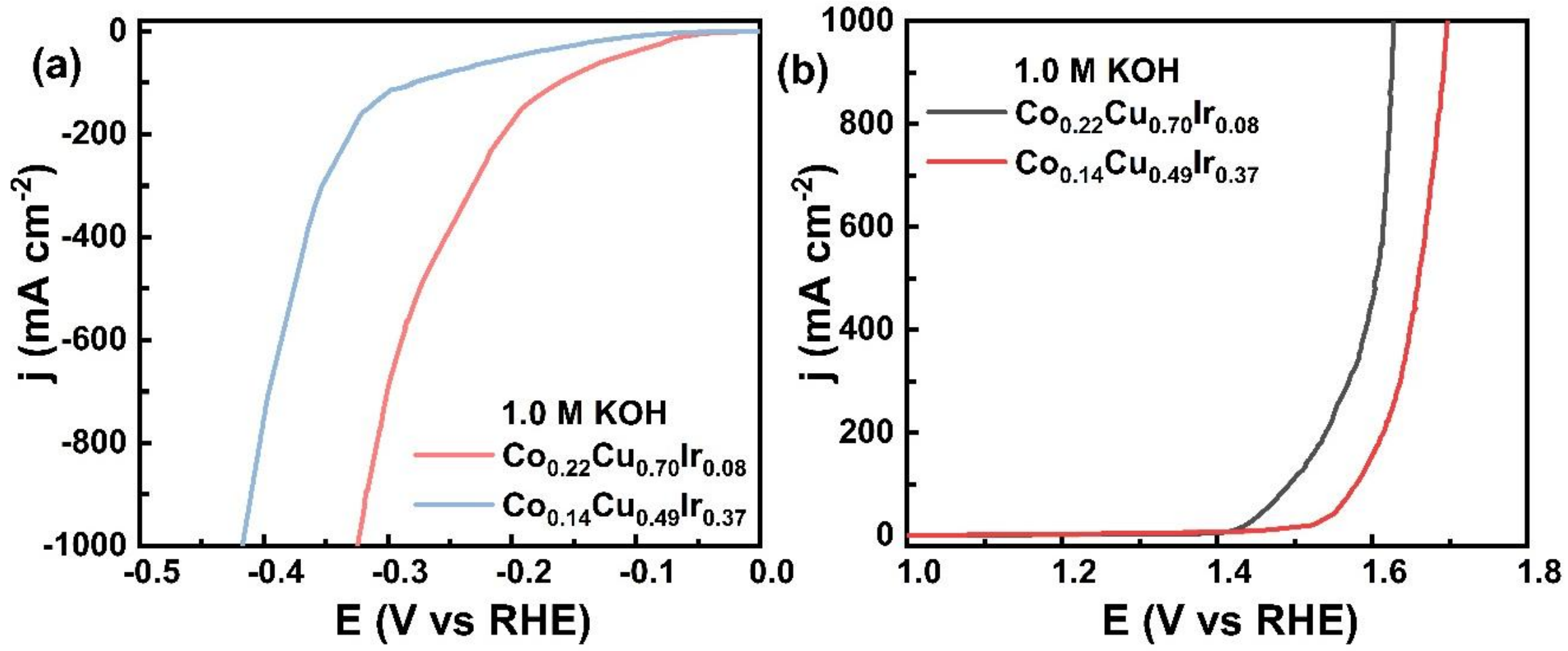

Figure S18. LSV comparison of the (a) HER and (b) OER for additional low- and high-Ir-content compositions of $Co_{0.22}Cu_{0.70}Ir_{0.08}$ and $Co_{0.14}Cu_{0.49}Ir_{0.37}$.

Table S17. Overpotential (mV) and Tafel slope (mV dec^{-1}) results of $Co_{0.28}Cu_{0.67}Ir_{0.05}$ in alkaline seawater (DSE).

S. No.	Catalysts	HER		OER	
		**Overpotential at 10 mA cm^{-2}**	**Tafel slope**	**Overpotential at 10 mA cm^{-2}**	**Tafel slope**
1.	$Co_{0.28}Cu_{0.67}Ir_{0.05}$	62	49.4	203	49.3
2.	Standard	53 (Pt-C)	43.9	310 (IrO_2)	139.4

Table S18. Summary of Exchange current density and fitting parameters of $Co_{0.28}Cu_{0.67}Ir_{0.05}$ catalysts in alkaline seawater conditions.

$Co_{0.28}Cu_{0.67}Ir_{0.05}$	Alkaline Seawater	
	**HER (-0.14 V vs RHE)**	**OER (1.48 V vs RHE)**
R_b (Ω)	3.97	2.97
R_{ct} (Ω)	5.06	3.19
CPE	8.98 mMho*s^0.624	13.3 mMho*s^0.588
i_{ex} (mA cm^{-2})	28.19	22.36

Table S19. Cell potential (V) data of $Co_{0.28}Cu_{0.67}Ir_{0.05}$ catalysts at various high current densities in various electrolytic conditions.

Electrolytes	$Co_{0.28}Cu_{0.67}Ir_{0.05}$ (graphite sheet substrate)		
	**Cell potential (V) at 10 mA cm^{-2}**	**Cell potential (V) at 100 mA cm^{-2}**	**Cell potential (V) at 1000 mA cm^{-2}**
1.0 M KOH	1.47	1.65	1.90
Alkaline Seawater	1.49	1.68	2.05
	**$Co_{0.28}Cu_{0.67}Ir_{0.05}$ (Ni-foam substrate)**		
	**Cell potential (V) at 10 mA cm^{-2}**	**Cell potential (V) at 1000 mA cm^{-2}**	**Cell potential (V) at 2000 mA cm^{-2}**
	1.46	1.81	1.848
	**Cell potential (V) at 2300 mA cm^{-2}** = 1.855		

Table S20. Summary of the OWS activity of the present $Co_{0.28}Cu_{0.67}Ir_{0.05}$ electrocatalysts with other reported electrocatalysts in alkaline condition.

**S. No.**	**Catalysts**	**Substrate**	**E_{cell} (V) at 10 mA cm^{-2}**	**Durability (h)**	**Reference**
1	$Co_{0.28}Cu_{0.67}Ir_{0.05}$	GS	1.47	150	This Work
2.	$Ru_{0.5}Ir_{0.5}$		1.48	100	Chemical Engineering Journal **450**, 137909 (2022)
3.	RuCu NCs	-	1.49	12	*Angew. Chem. Int. Ed.* **58**, 13983-13988 (2019)
4.	RuCo@CDs	-	1.50	12	*J. Mater. Chem. A*, **8**, 9638-9645 (2020)
5.	RuNi nanoplates	CFP	1.48	40	Nano Energy **47**, 1-7 (2018)
6.	RhCu NTs	CP	1.59	30	Adv. Energy Mater. **10**, 1903038 (2020)
7.	NiIr@N−C	NF	1.50	25	Chem. Eur. J. **26**, 14496 – 14501 (2020)
8.	$Ir_{0.5}W_{0.5}$	CC	1.60	16.7	*Nanoscale*, **11**, 8898-8905 (2019)
9.	FeIr	NF	1.48	500	Applied Catalysis B: Environmental **278**, 1193 (2020)
10.	$Ni_{0.93}Ir_{0.07}$/rGO	NF	1.52	24	Journal of Energy Chemistry **49**, 166–173 (2020)

11.	$FeCoMoO_4$	NF	1.54	100	Chemical Eng. J. **520**, 165885 (2025)
12.	10%P-RuO_2 NFs	NF	1.52	100	Adv. Energy Mat. **15**, 2403136 (2026)
13.	FeO_x/Rh	Carbon fiber paper	1.53	25	Adv. Sci. **13**, e11701 (2026)
14.	MoO_2@N/Mo–ReS_2	Carbon cloth	1.54	90	Small **21**, 2505906 (2026)
15.	FeV-NiZnN	NF	1.63	7	Small **22**, e13183 (2026)
16.	Fe-NiMo‖Fe-NiMo	NF	1.49	200	Chemical Eng. J. **536**, 175938 (2026)
17.	FeOOH@Ni_3N/NF	NF	1.49	100	Small **22**, e13136 (2026)
18.	FVNOOH(+)‖FVNP(−)	NF	1.50	300	Appl. Catal. B: Environ. Energy **387**, 126477 (2026)
19.	FO-FP@NS/NF	NF	1.47	--	Energy Environ. Mater. **9**, e70187 (2026)
20.	Co/$Co_2N_{0.67}$/CeO_x/C-PCFs	carbon fibers	1.52	240	Appl. Surf. Sci. **746**, 167570 (2026)

Table S21. Comparison of the OWS performance of $Co_{0.28}Cu_{0.67}Ir_{0.05}$ nanostructure with previously reported electrocatalysts in ***alkaline seawater electrolyte***.

**S. No.**	**Catalysts**	**Max current density (mA cm^{-2})#**	**Catalyst loading (mg cm^{-2})**	**F. E. (%) HER, OER**	**E_{cell} (V) at 10 mA cm^{-2} (substrate)**	**Stability test (h)**	***iR* correction (%)**	**T (ºC)**	**Reference**
1.	$Co_{0.28}Cu_{0.67}Ir_{0.05}$	2300 (NF)	1.09	96.34 95.12	1.49 (GS) 1.46 (NF)	120	90	25	This Work
2.	NiFe/Fe-MoO_2	~160	8.6	100, 100	1.51 (NF)	50	No	25	Small **18**, 2205683 (2022)
3.	Ru-$NiMoP_2$	~160	-	-	1.53 (NF)	10	No	25	Sustainable Energy Fuels**,** **7**, 4677-4686 (2023)
4.	CoPx‖CoPx@FeOOH	800	1.33	98,98	1.549 (NF)	80	-	25	Appl. Catal. B: Environ. **294**, 120256 (2021)
5.	CoFeOF	~750	-	100, 100	1.61 (NF)	140	-	25	Chem. Eng. J **480**, 146545–146545 (2023)
6.	NiFeP/NF	1000	0.1	-,99.5	1.62 (NF)	500	70	25	Appl. Catal., B, **302**, 120862 (2022)
7.	Mn-doped Ni_2P/Fe_2P	~600	-	100, 100	1.64 (NF)	120	No	25	Chem. Eng. J. **454**,

									140061, (2023).
8.	NiFeS	500	-	-	1.67 (NF)	25	No	25	J. Mater. Chem. A **11**, 1116-1122 (2023)
9.	FeNiCoMnRu@CNT*	~300	1	100, 100	1.6	30	90	25	J. Colloid Interface Sci. **646**, 844–854 (2023)
10.	Ru-CoOx	1000	-	100,100	1.86 (NF)@ 100 mA cm^{-2}	-	-	25	Small **17**, 2102777 (2021)
11.	Mo_3Se_4–NiSe	HER specific 500	1.79	100,100	1.71 (NF)	50	No	25	Adv. Mater. 2024, 36, 2305813
12.	S-NiCoP	60	-	97.8, 94.6	1.68 (CC)	200	95	25	Chem. Eng. J. **510**, 161709 (2025)
13.	e-CoNiFeP	~40	-	-	1.59 (NF)	130	90	25	J. Colloid Interface Sci. **698**, 138089 (2025)
14.	$FeCoMoO_4$	~500	-	100, 100	1.45 (NF)@ 100 mA cm^{-2}	100	No	25	Chem. Eng. J. **520**, 165885 (2025)
15.	$CoP@Fe_3N-Ni_4N$	~155	3.2	97.5, 94.6	1.51 (NF)	200	-	25	Chem. Eng. J. **535**,

									175340 (2026)
16.	NiRu-RuO_2/NiO‖NiFe**	~450	6 mg	100, 100	1.45 (TF)	200	95	25	Appl. Catal. B: Environ. Energy. **392**, 126715 (2026)
17.	Fe-Co_3O_4|Vo	~240	7.2	100, 100	1.59 (NF)	100	-	25	ACS Sustainable Chem. Eng. **14**, 3023 (2026)
18.	Si-CoFe-LDH	~350	-	100, 100	1.62 (NF)	55	90	25	Appl. Catal. B: Environ. Energy **391**, 126714 (2026)
19.	Ni_3N@$NiFeHPO_x$	~1100	-	94, 94	1.51 (NF)	36	85	25	J. Colloid Interface Sci. **701**, 138622 (2026)

All substrate nickel foam, except *carbon paper, **Titanium foam, Electrolyte: Alkaline seawater (1.0M KOH + natural seawater), #the maximum current density values are taken from the LSV plots, T is temperature.

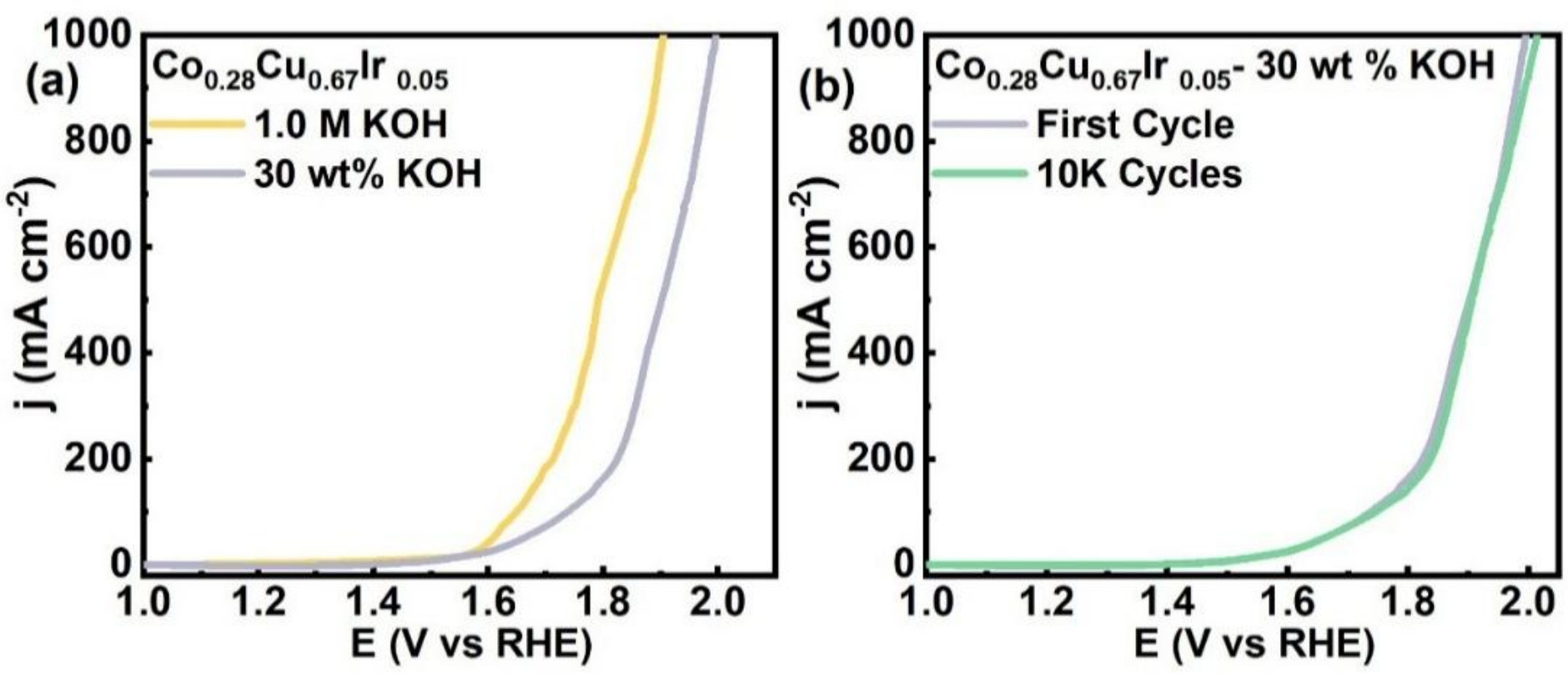

Figure S19. (a) OWS polarization curves of the $Co_{0.28}Cu_{0.67}Ir_{0.05}$ 1.0 M KOH and 30 wt% KOH electrolytes. (b) Cycling stability test of the $Co_{0.28}Cu_{0.67}Ir_{0.05}$ under 30 wt% KOH condition.

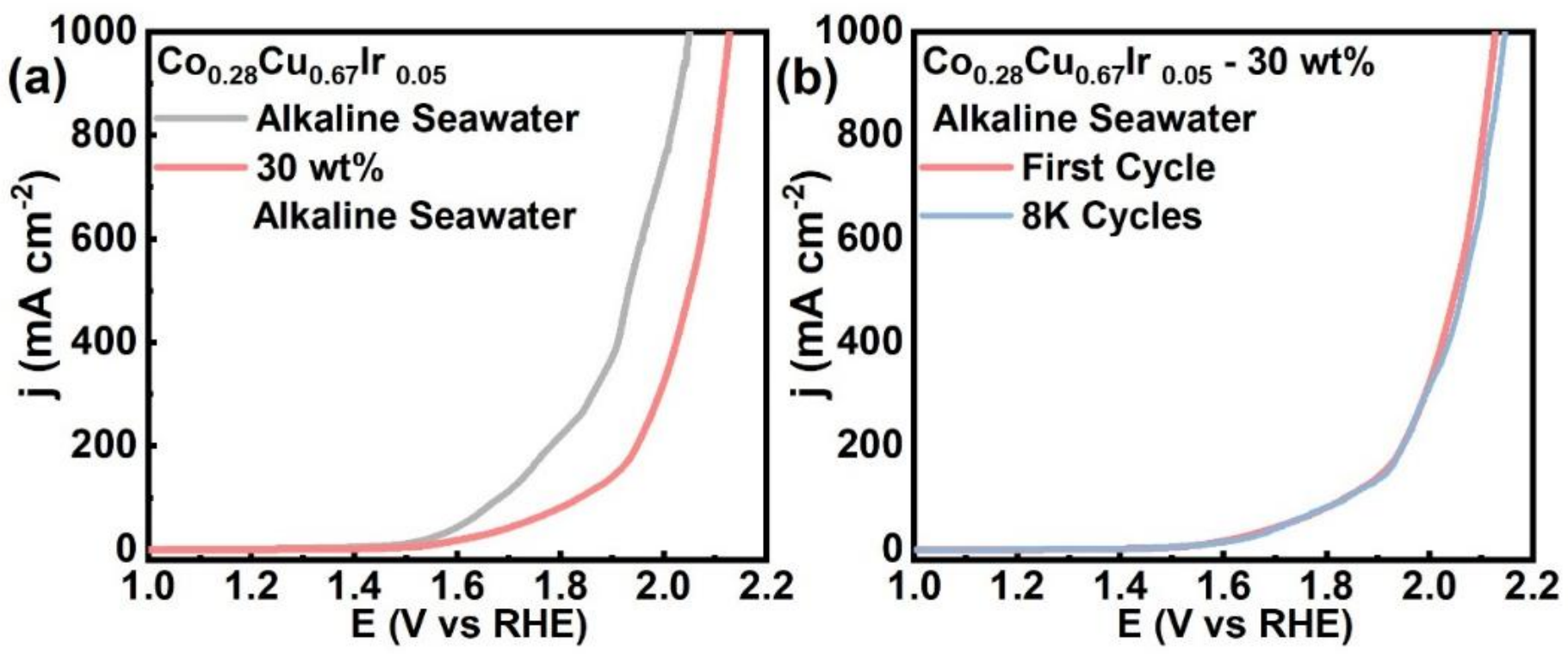

Figure S20. (a) OWS polarization curves of the $Co_{0.28}Cu_{0.67}Ir_{0.05}$ in alkaline seawater and 30 wt% alkaline seawater electrolytes, and (b) Cycling stability test of the $Co_{0.28}Cu_{0.67}Ir_{0.05}$ 30 wt% alkaline seawater medium.

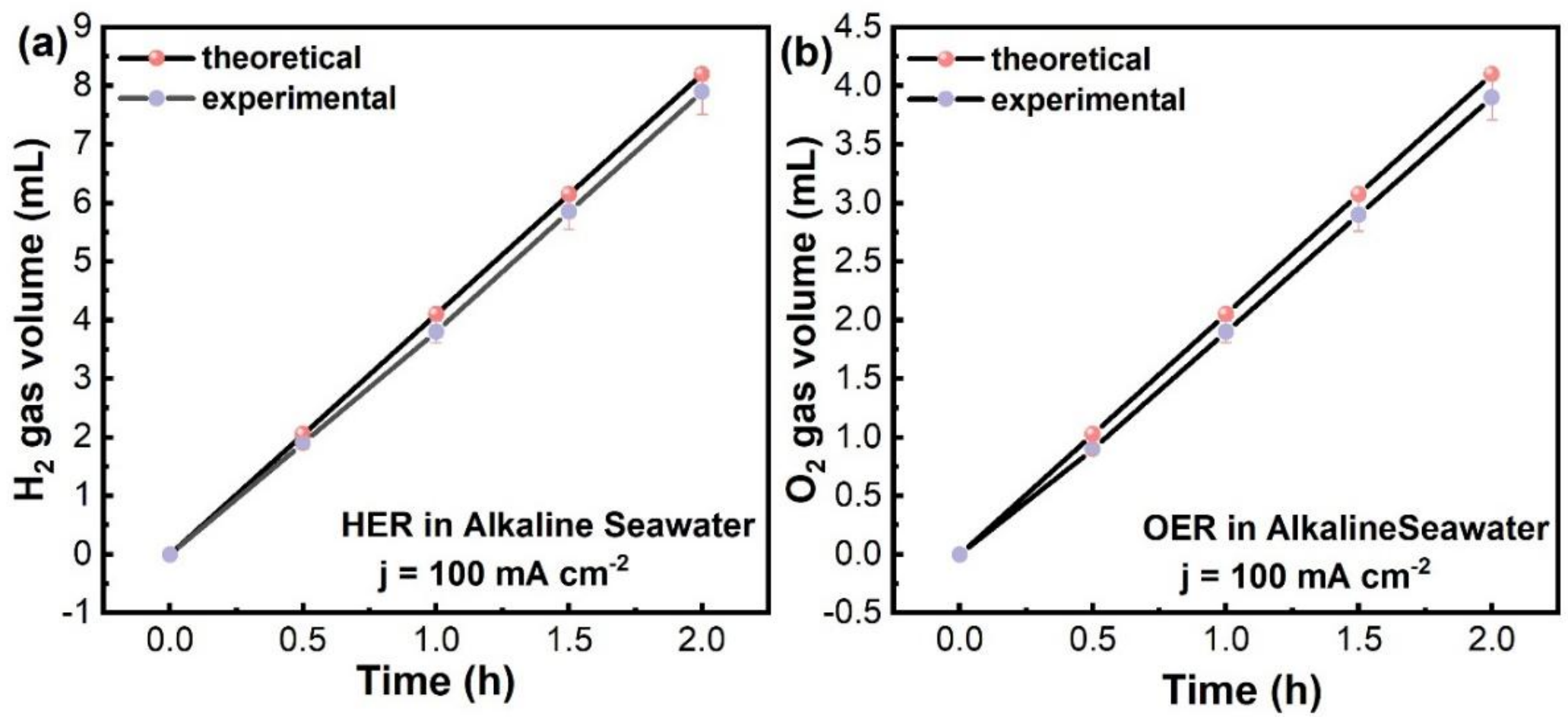

Figure S21. Faradaic efficiency evaluation of the $Co_{0.28}Cu_{0.67}Ir_{0.05}$ catalyst in alkaline seawater. Comparison of the theoretical and experimentally measured hydrogen gas volumes during (a) HER and (b) OER.

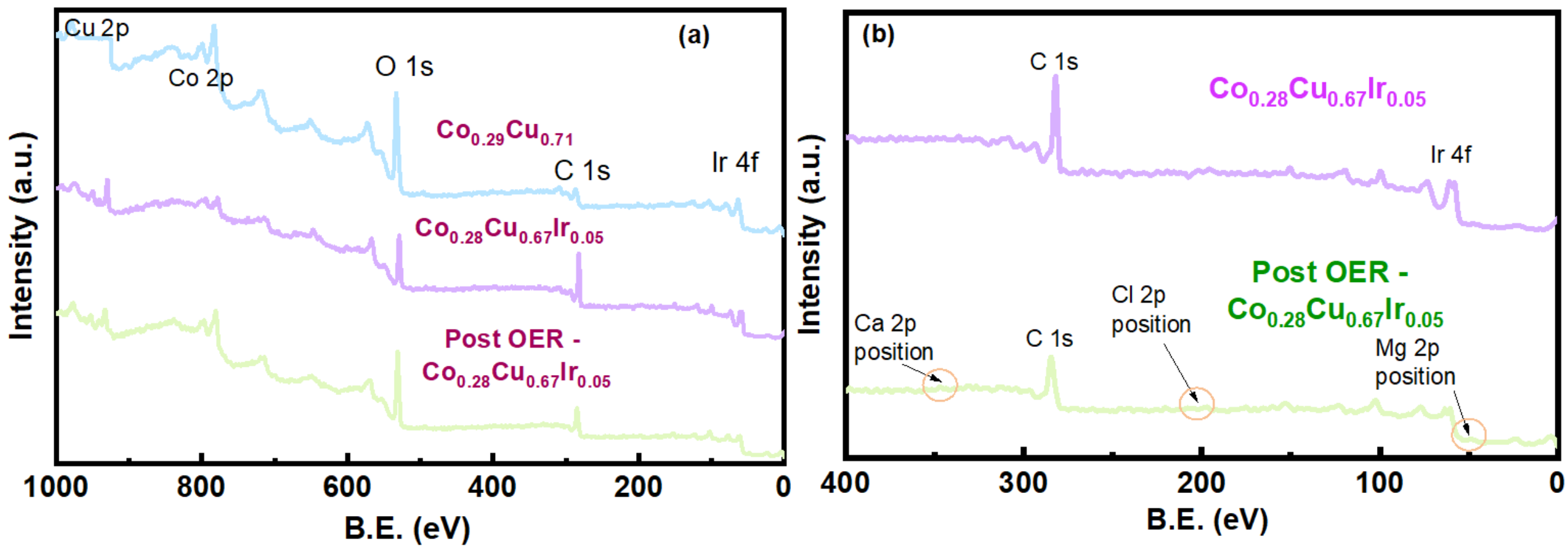

Figure S22. (a) XPS survey spectrum of $Co_{0.29}Cu_{0.71}$, $Co_{0.28}Cu_{0.67}Ir_{0.05}$, and Post-OER $Co_{0.28}Cu_{0.67}Ir_{0.05}$ after alkaline seawater electrolysis. (b) Enlarged XPS survey spectra (before and after DSE) showing the standard peak positions for Cl, Ca, and Mg species.

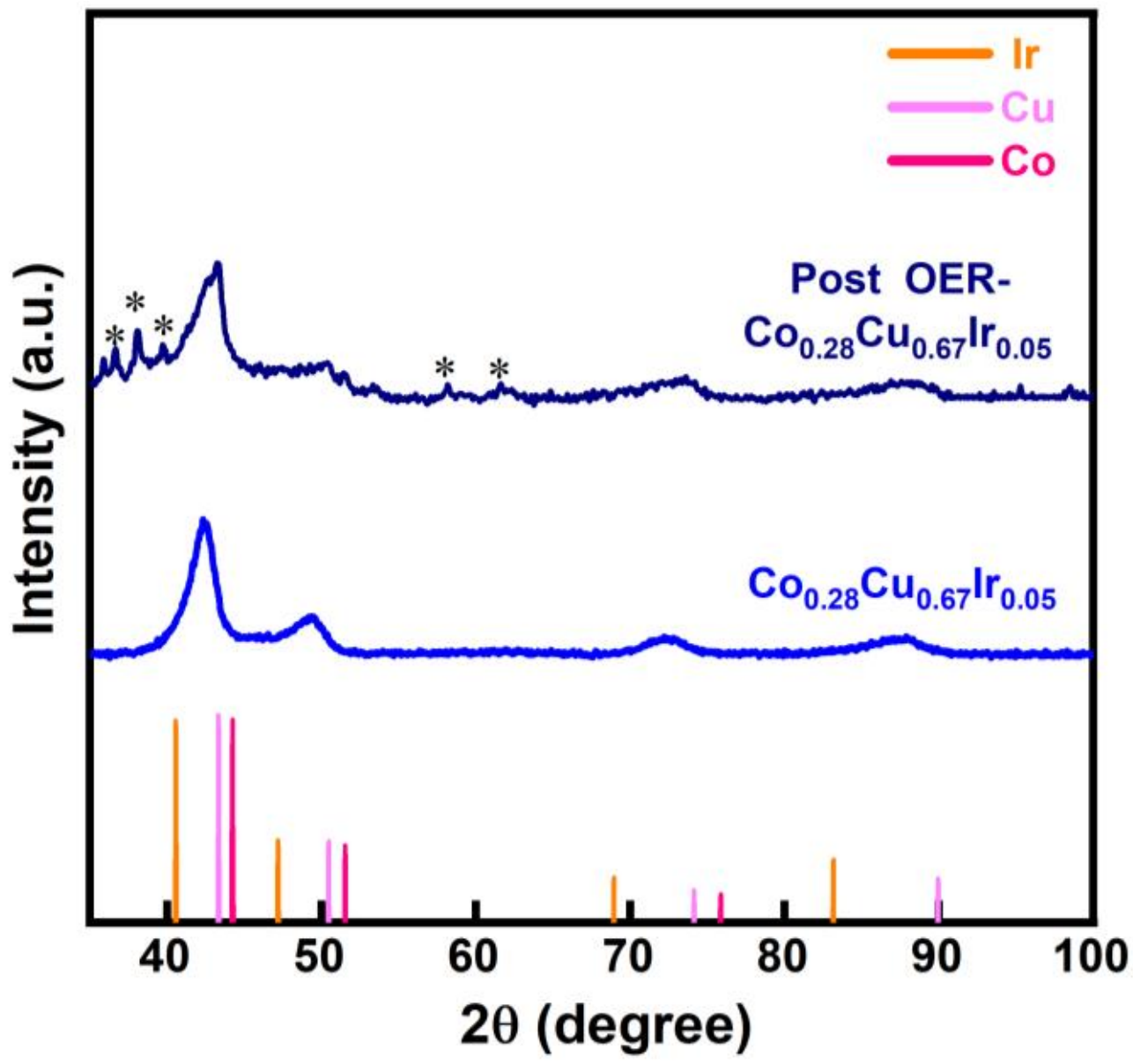

Figure S23. Powder XRD comparison of fresh $Co_{0.28}Cu_{0.67}Ir_{0.05}$ and Post-OER $Co_{0.28}Cu_{0.67}Ir_{0.05}$ after alkaline seawater electrolysis.

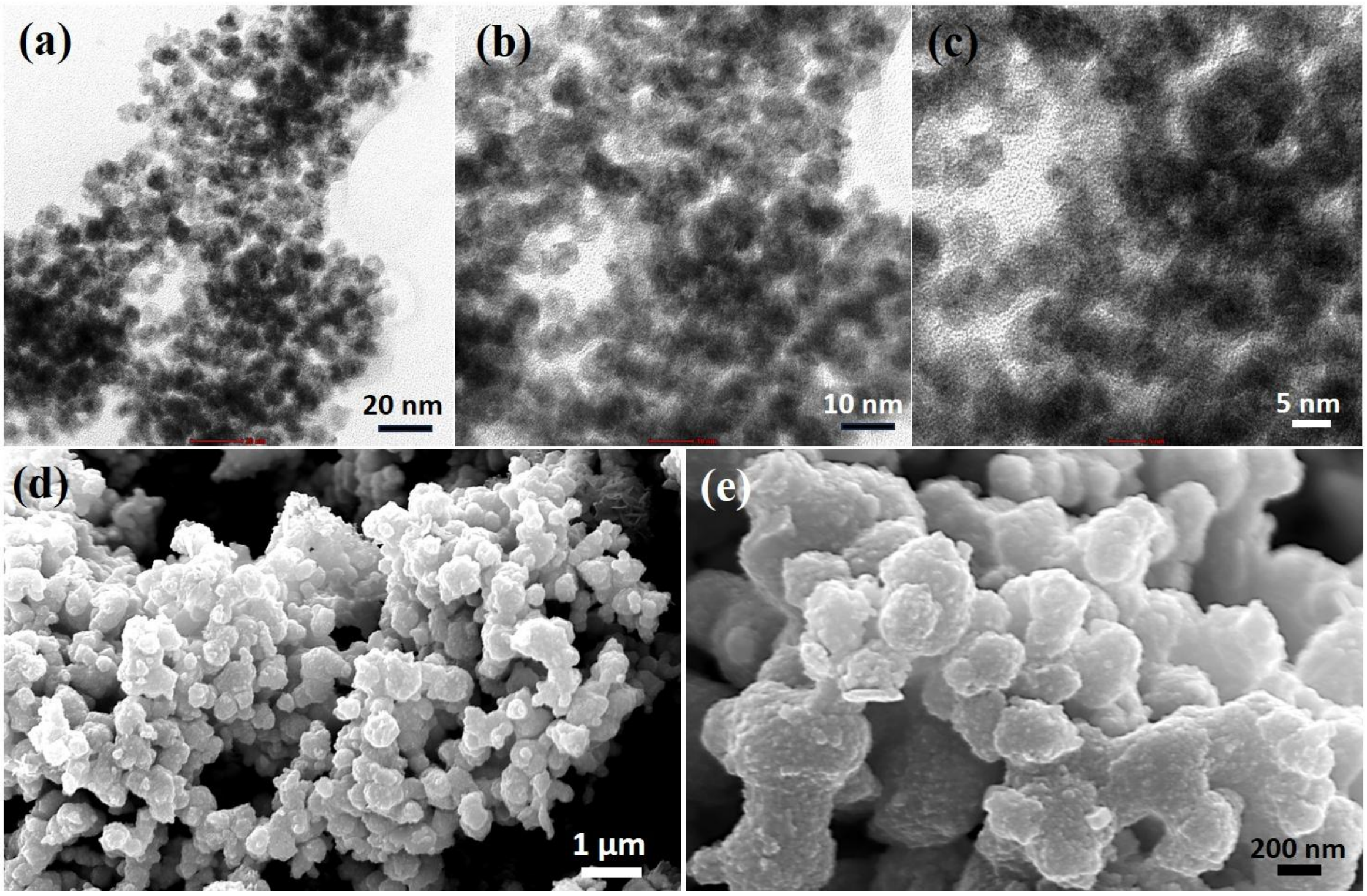

Figure S24. (a-c) TEM and (d, e) FESEM images of $Co_{0.28}Cu_{0.67}Ir_{0.05}$ Post OER catalysis after DSE.

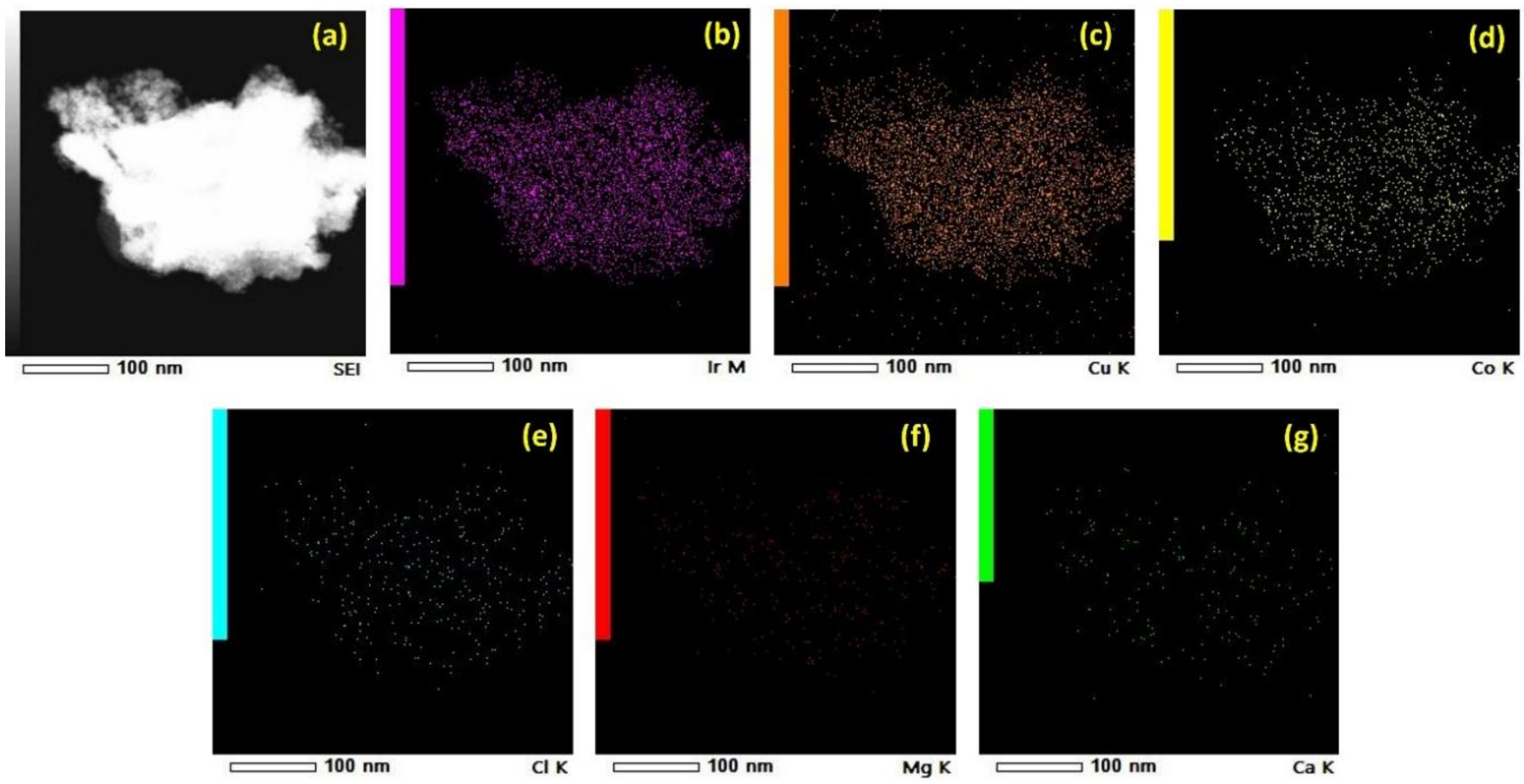

Figure S25. HRTEM image and HRTEM-EDS elemental mapping of the $Co_{0.28}Cu_{0.67}Ir_{0.05}$ anode sample after 50 h of chronopotentiometric electrolysis at 2 A cm^{-2}. (a) HRTEM image of the selected region for HRTEM-EDS analysis. Elemental maps of (b) Ir, (c) Cu, (d) Co, (e) Cl, (f) Mg, and (g) Ca.

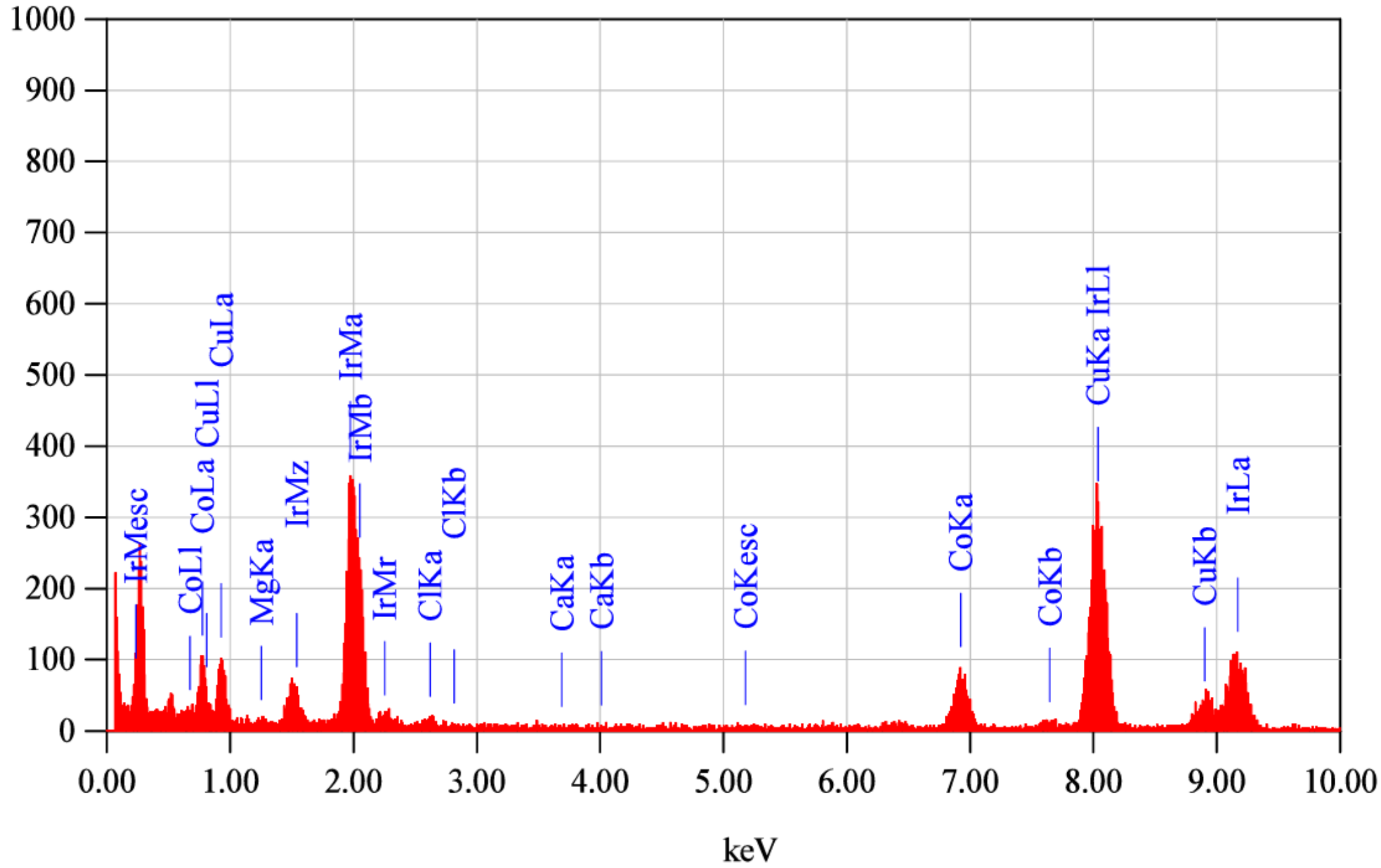

Figure S26. HRTEM-EDAX spectrum of the $Co_{0.28}Cu_{0.67}Ir_{0.05}$ anode sample after 50 h of chronopotentiometric electrolysis at 2 A cm^{-2}.

Table S22. TEM-EDAX surface elemental composition in $Co_{0.28}Cu_{0.67}Ir_{0.05}$ anode sample after 50 h of chronopotentiometric electrolysis at 2.0 A cm^{-2}.

**Element**	**X-ray Line**	**Energy (keV)**	**Weight (%)**	**Atomic (%)**	**Counts**	**Sigma**	**K-ratio**
Mg	K	1.253	0.25	0.96	57.30	0.08	0.4876
Cl	K	2.621	N.D.	N.D.	N.D.	N.D.	N.D.
Ca	K	3.690	0.40	1.05	82.31	0.10	0.5424
Co	K	6.924	7.70	12.28	3353.81	1.63	0.8475
Cu	K	8.040	41.27	61.06	4658.61	1.31	1.0000
Ir	M	1.977	50.39	24.65	1025.50	0.52	1.6959
Total			100	100			

*N.D. denotes not detected.

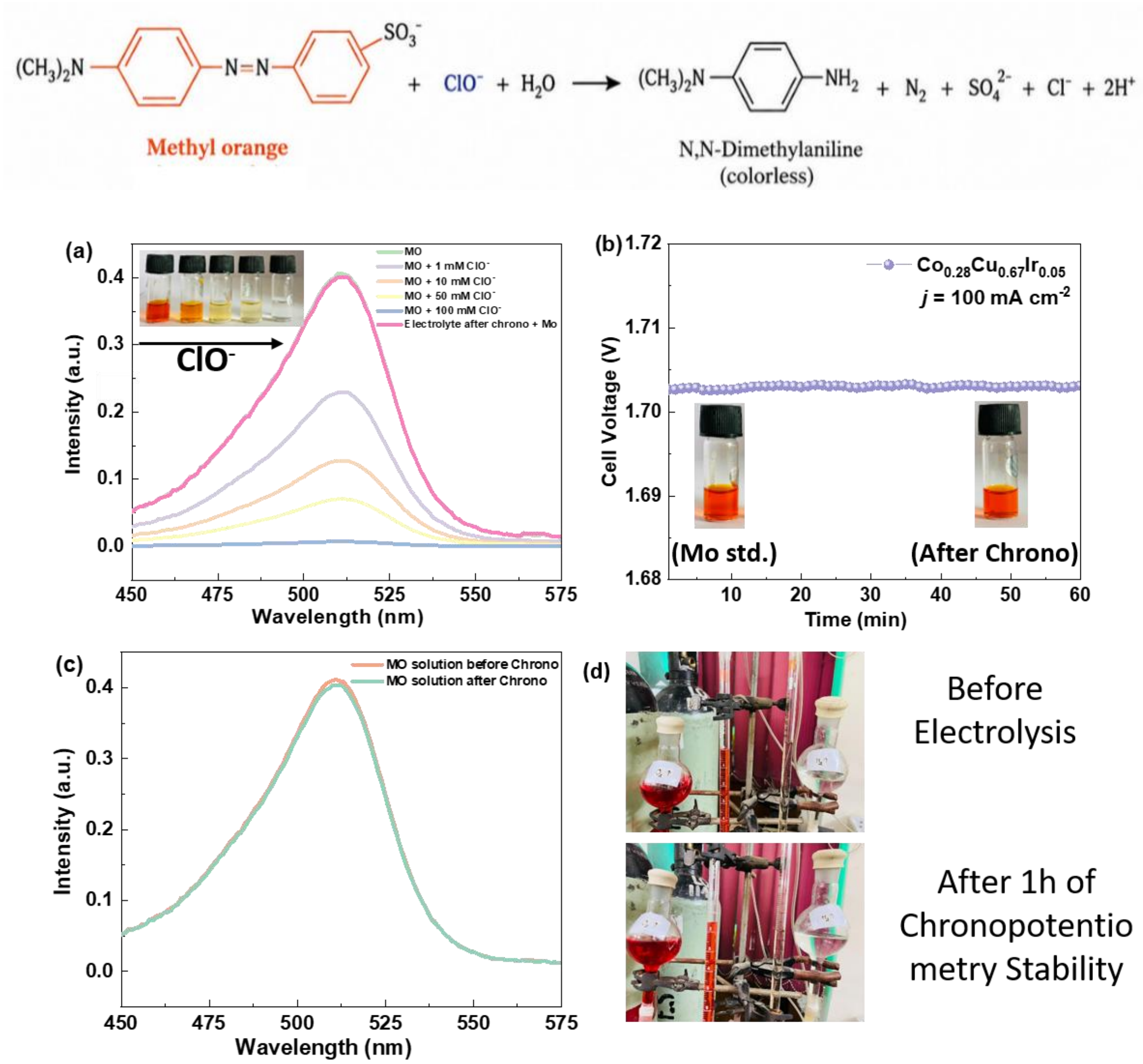

Figure S27. Assessment of chlorine-derived byproduct formation during alkaline seawater electrolysis. **(a)** UV–Vis spectra of methyl orange (MO) with increasing ClO^- concentrations, showing progressive bleaching of the characteristic absorption band. **(b)** Chronopotentiometric test of the $Co_{0.28}Cu_{0.67}Ir_{0.05}$ electrode at 100 mA cm^{-2} for 1 h, with photographs of the MO solution before and after electrolysis. **(c)** UV–Vis spectra of the MO solution before and after chronopotentiometry, exhibiting negligible spectral changes **(d)** Photographs of the electrolysis setup and collected MO solutions before and after operation, indicating suppressed chlorine/hypochlorite formation and high OER selectivity.

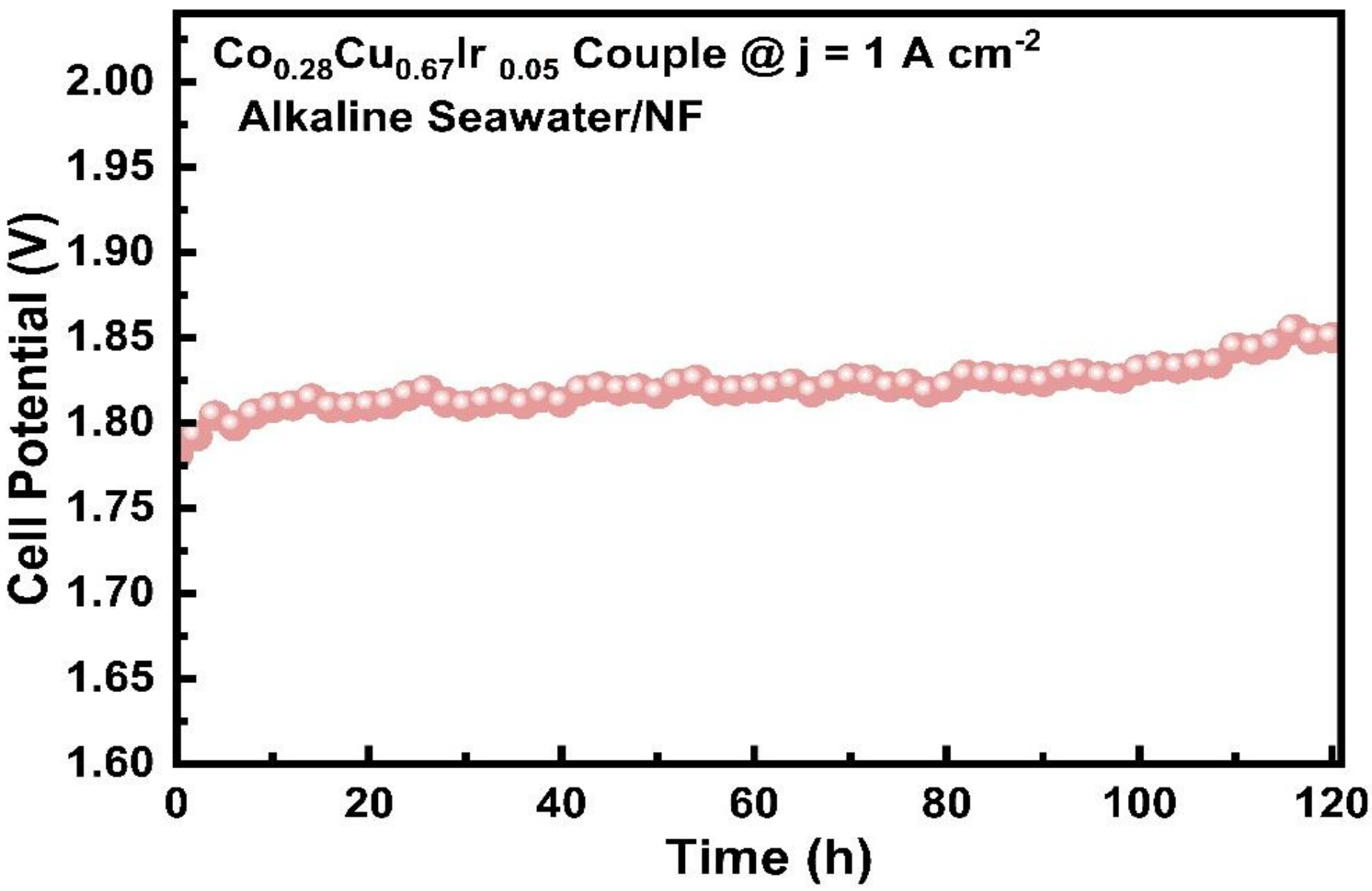

Figure S28. Chronopotentiometric stability test of the $Co_{0.28}Cu_{0.67}Ir_{0.05}$ couple at a current density of 1 A cm^{-2} in alkaline seawater electrolytes under NF substrate.

Table S23. ICP–MS analysis of metal ion concentrations in the electrolyte before and after OER stability testing.

**Sample**	**Composition (ICP-MS derived atomic ratio)**	**Elemental ratio (μg/g)**				
		**Co**	**Cu**	**Ir**	**Ca**	**Mg**
**Fresh catalyst**	**$Co_{0.28}Cu_{0.67}Ir_{0.05}$**	165822.7273	392439.3939	38142.4242	N.D.	N.D.
**Post-catalysis**	**$Co_{0.292}Cu_{0.663}Ir_{0.045}$**	156549.1542	354439.5386	23896.3845	876.12	N.D,

Note: ND = Not Detected.

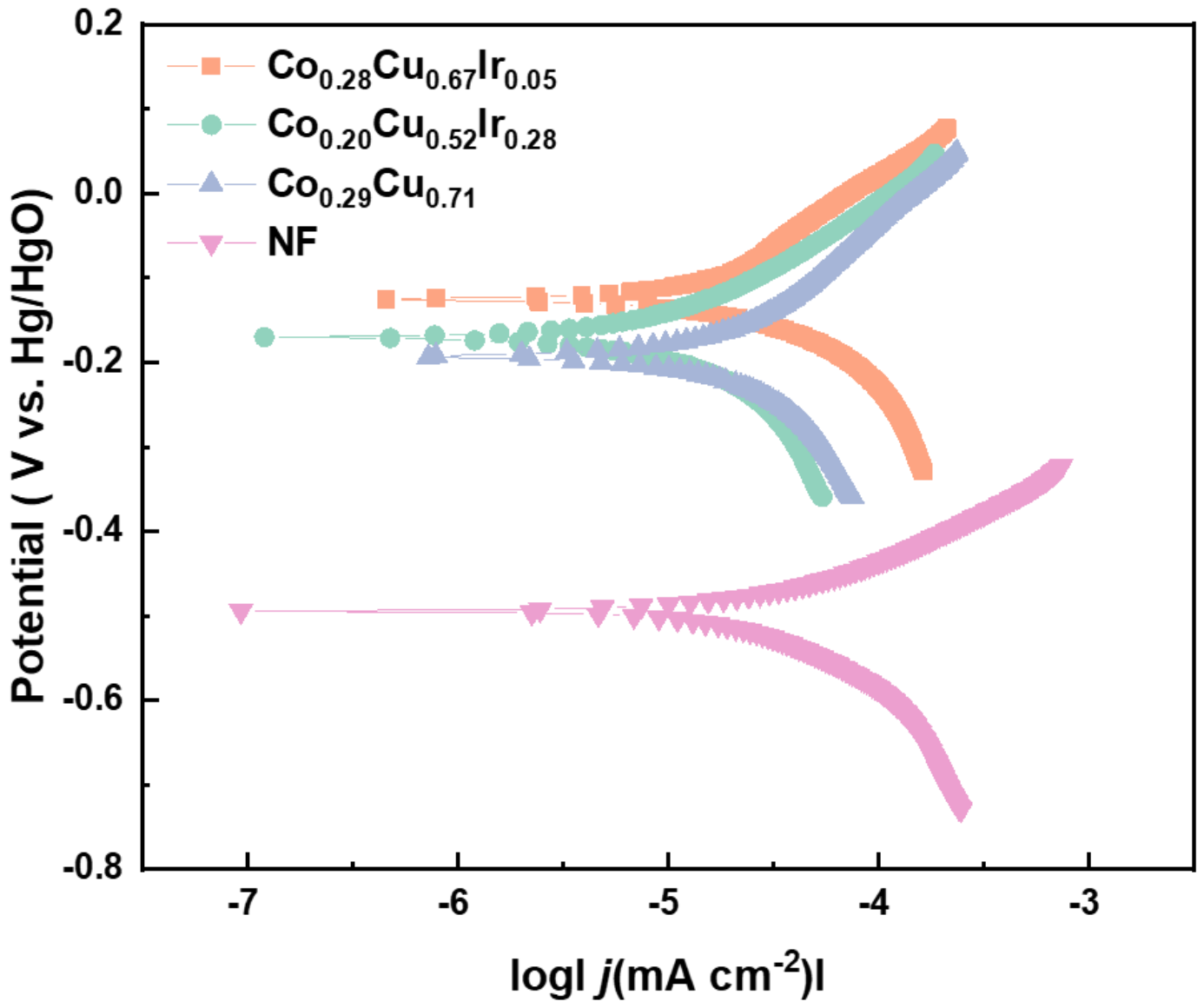

Figure S29. Chlorine corrosion polarization curves of $Co_{0.28}Cu_{0.67}Ir_{0.05}$, $Co_{0.20}Cu_{0.52}Ir_{0.28}$, $Co_{0.29}Cu_{0.71}$ and bare Ni foam (NF). $Co_{0.28}Cu_{0.67}Ir_{0.05}$ shows the most positive corrosion potential, indicating superior chlorine tolerance and corrosion resistance.

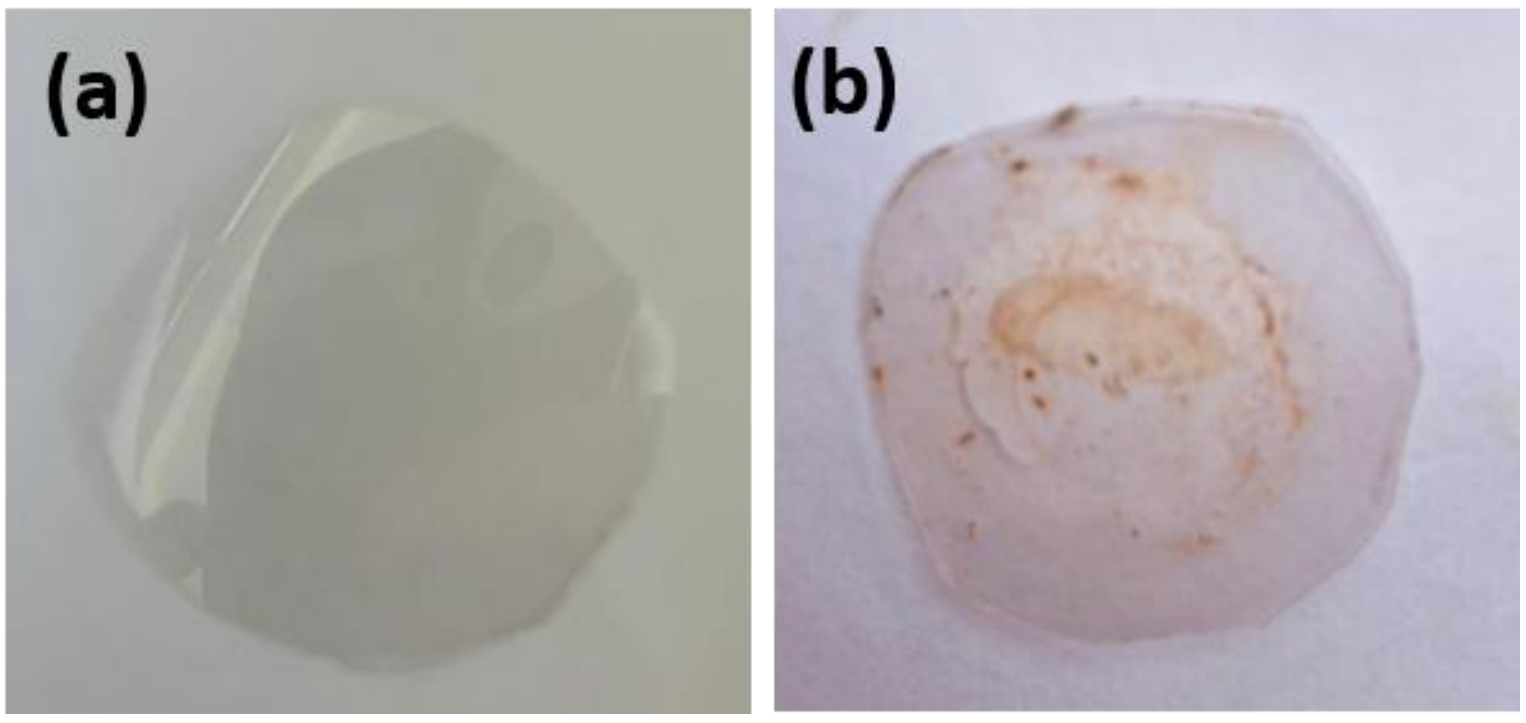

Figure S30. Photographs of the membrane (a) before and (b) after long-term alkaline seawater electrolysis, showing the preservation of membrane integrity with only minor surface deposits observed after operation.

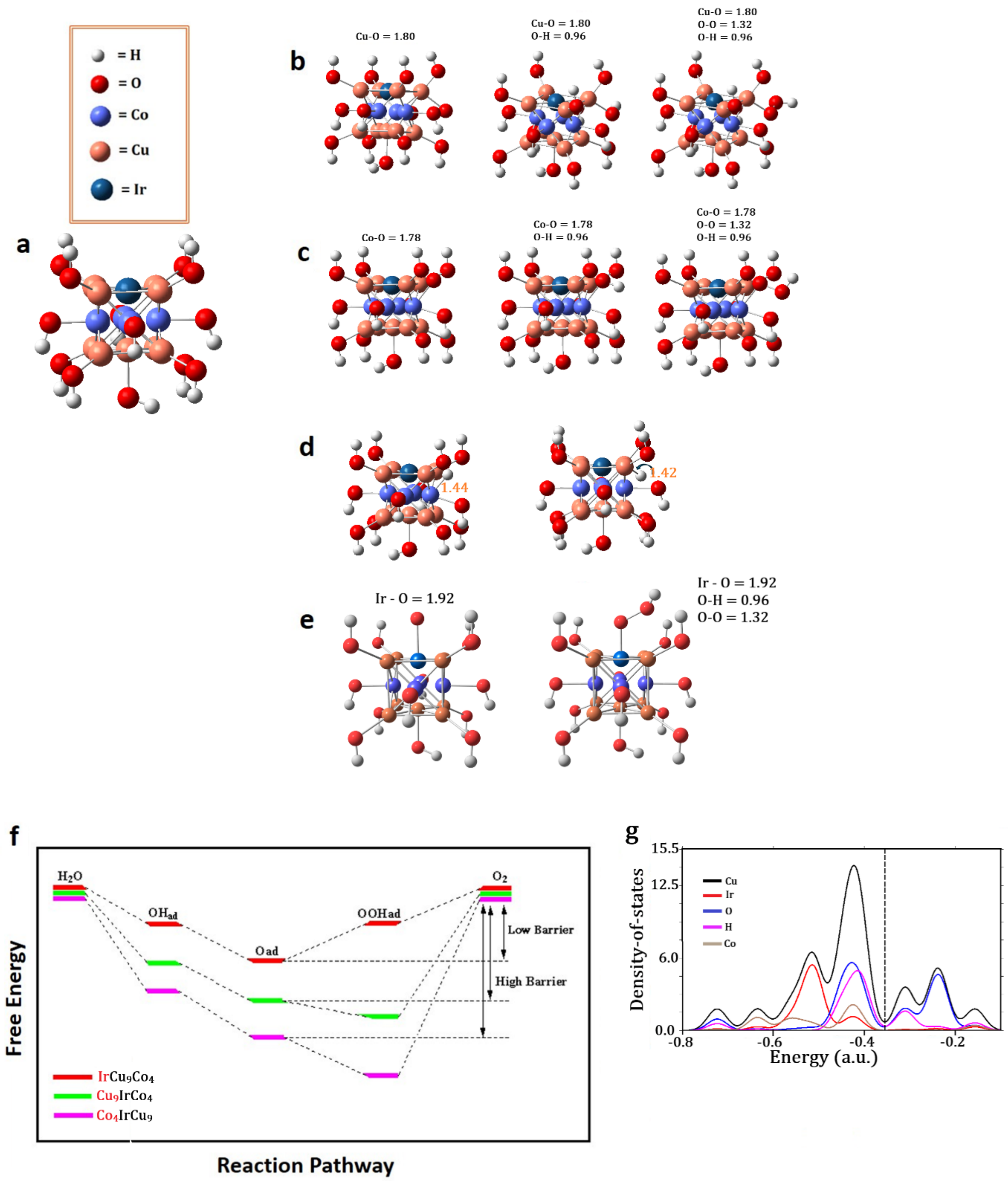

Figure S31. Hydroxide-coated $Co_{4.0}Cu_{9.0}Ir_{1.0}$ unit cell: (a) Minimum energy structures of the hydroxide-coated $Co_{4.0}Cu_{9.0}Ir_{1.0}$ unit cell. (b-e) Optimized geometries of the O-bound intermediates with possible absorption sites for OER steps on $Co_{4.0}Cu_{9.0}Ir_{1.0}$ unit cell. Bond lengths are given in Å. (f) Schematic illustration for the OER on $Co_{4.0}Cu_{9.0}Ir_{1.0}$ unit cell. (g) Partial density of states plot of hydroxide-coated $Co_{4.0}Cu_{9.0}Ir_{1.0}$ unit cell.

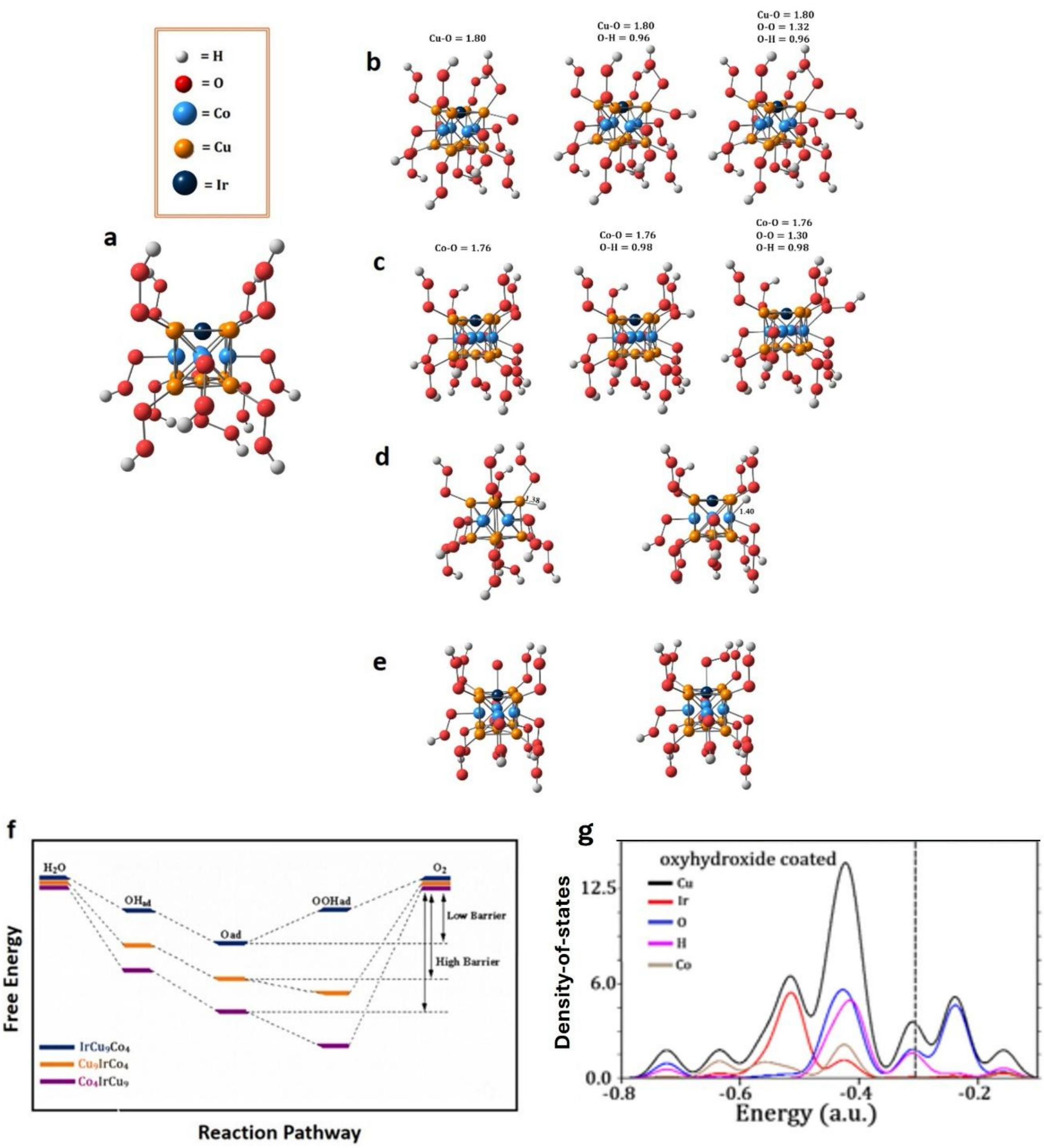

Figure S32. (a) Minimum energy structures of the oxyhydroxide-coated $Co_{4.0}Cu_{9.0}Ir_{1.0}$ unit cell. (b-e) Optimized geometries of the O-bound intermediates with possible absorption sites for OER steps on the $Co_{4.0}Cu_{9.0}Ir_{1.0}$ unit cell. Bond lengths are given in Å. (f) Schematic illustration for the OER on oxyhydroxide-coated $Co_{4.0}Cu_{9.0}Ir_{1.0}$ unit cell. (g) Partial density of states plot of oxyhydroxide-coated $Co_{4.0}Cu_{9.0}Ir_{1.0}$ unit cell.

References

1. S. Basu, et al., *J. Phys.: Conf. Ser.* **2014**, *493*, 012032.

2. M. E. Fitzpatrick, A. T. Fry, P. Holdway, F. A. Kandil, J. Shackleton, L. Suominen, *Determination of Residual Stresses by X-ray Diffraction*, National Physical Laboratory, **2002**.
3. B. D. Cullity, S. R. Stock, *Elements of X-Ray Diffraction*, Pearson Education UK, **2013**.
4. L. Cao, et al., *Phys. Chem. Chem. Phys.* **2015**, *17*, 6809-6818.
5. R. W. G. Wyckoff, *Crystal Structures*, Vol. 1, 2nd ed., Interscience Publishers, New York **1963**, *1*. 7–83.
6. E. A. Owen, E. L. Yates, *Philos. Mag.* **1933**, *15*, 472-488.
7. M. Chauhan, K. P. Reddy, C. S. Gopinath, S. Deka, *ACS Catal.* **2017**, *7*, 5871-5879.
8. H. Han, et al., *Adv. Energy Mater.* **2019**, *9*, 1803799.
9. X. Shang, et al., *Nanoscale* **2017**, *9*, 12353-12363.
10. C. C. L. McCrory, S. Jung, J. C. Peters, T. F. Jaramillo, *J. Am. Chem. Soc.* **2013**, *135*, 16977-16987.
11. X. Zhu, et al., *J. Mater. Chem. A* **2016**, *4*, 7245-7250.
12. B. H. R. Suryanto, Y. Wang, R. K. Hocking, W. Adamson, C. Zhao, *Nat. Commun.* **2019**, *10*, 5599.
13. H. Zhang, J. Zheng, Y. Chao, K. Zhang, Z. Zhu, *New J. Chem.* **2018**, *42*, 7254-7261.
14. F. Urbain, V. Smirnov, J.-P. Becker, A. Lambertz, F. Yang, J. Ziegler, B. Kaiser, W. Jaegermann, U. Rau, F. Finger, *Energy Environ. Sci.* **2016**, *9*, 145-154.
15. J. P. Perdew, K. Burke, M. Ernzerhof, *Phys. Rev. Lett.* **1996**, *77*, 3865-3868.
16. M. J. Frisch, G. W. Trucks, H. B. Schlegel, G. E. Scuseria, M. A. Robb, J. R. Cheeseman, G. Scalmani, V. Barone, G. A. Petersson, H. Nakatsuji, X. Li, M. Caricato, A. V. Marenich, J. Bloino, B. G. Janesko, R. Gomperts, B. Mennucci, H. P. Hratchian, J. V. Ortiz, A. F. Izmaylov, J. L. Sonnenberg, D. Williams-Young, F. Ding, F. Lipparini, F. Egidi, J. Goings, B. Peng, A. Petrone, T. Henderson, D. Ranasinghe, V. G. Zakrzewski, J. Gao, N. Rega, G. Zheng, W. Liang, M. Hada, M. Ehara, K. Toyota, R. Fukuda, J. Hasegawa, M. Ishida, T. Nakajima, Y. Honda, O. Kitao, H. Nakai, T. Vreven, K. Throssell, J. A. Montgomery, Jr., J. E. Peralta, F. Ogliaro, M. J. Bearpark, J. J. Heyd, E. N. Brothers, K. N. Kudin, V. N. Staroverov, T. A. Keith, R. Kobayashi, J. Normand, K. Raghavachari, A. P. Rendell, J. C. Burant, S. S. Iyengar, J. Tomasi, M. Cossi, J. M. Millam, M. Klene, C. Adamo, R. Cammi, J. W. Ochterski, R. L. Martin, K. Morokuma, O. Farkas, J. B. Foresman, D. J. Fox, *Gaussian 16, Revision B.01*, Gaussian, Inc., Wallingford CT **2016**.

17. T. Lu, F. Chen, *J. Comput. Chem.* **2012**, *33*, 580.

****
